# Intratumor heterogeneity inferred from targeted deep sequencing as a prognostic indicator

**DOI:** 10.1038/s41598-019-41098-0

**Published:** 2019-03-14

**Authors:** Bo Young Oh, Hyun-Tae Shin, Jae Won Yun, Kyu-Tae Kim, Jinho Kim, Joon Seol Bae, Yong Beom Cho, Woo Yong Lee, Seong Hyeon Yun, Yoon Ah Park, Yeon Hee Park, Young-Hyuck Im, Jeeyun Lee, Je-Gun Joung, Hee Cheol Kim, Woong-Yang Park

**Affiliations:** 1 Department of Colorectal Surgery, Hallym University Sacred Heart Hospital, Hallym University College of Medicine, Anyang, Korea; 2 Samsung Genome Institute, Samsung Medical Center, Seoul, Korea; 3 New York Genome Center, New York, NY USA; 4 Department of Surgery, Samsung Medical Center, Sungkyunkwan University School of Medicine, Seoul, Korea; 5 Department of Health Sciences and Technology, Samsung Advanced Institute of Science and Health Technology, Sungkyunkwan University, Seoul, Korea; 6 Division of Hematology and Oncology, Department of Medicine, Samsung Medical Center, Sungkyunkwan University School of Medicine, Seoul, Korea; 7 Department of Molecular Cell Biology, Sungkyunkwan University School of Medicine, Seoul, Korea; 8 GENINUS Inc., Seoul, Korea

## Abstract

Tumor genetic heterogeneity may underlie poor clinical outcomes because diverse subclones could be comprised of metastatic and drug resistant cells. Targeted deep sequencing has been used widely as a diagnostic tool to identify actionable mutations in cancer patients. In this study, we evaluated the clinical utility of estimating tumor heterogeneity using targeted panel sequencing data. We investigated the prognostic impact of a tumor heterogeneity (TH) index on clinical outcomes, using mutational profiles from targeted deep sequencing data acquired from 1,352 patients across 8 cancer types. The TH index tended to be increased in high pathological stage disease in several cancer types, indicating clonal expansion of cancer cells as tumor progression proceeds. In colorectal cancer patients, TH index values also correlated significantly with clinical prognosis. Integration of the TH index with genomic and clinical features could improve the power of risk prediction for clinical outcomes. In conclusion, deep sequencing to determine the TH index could serve as a promising prognostic indicator in cancer patients.

## Introduction

Intratumor heterogeneity (ITH) refers to the concept of a single tumor being comprised of many different subpopulations of cells^1^. These cell subpopulations can show distinct morphological and phenotypic patterns, with differences of gene expression and metastatic potential^2^. An increasing number of studies have observed ITH at distinct regions or in individual cells, and each cell subpopulation harbors a group of mutations that are likely to occur at the same stage^3^. Oncogenic mutations within a subclone are likely to play a crucial role in cancer recurrence, metastasis, and chemoresistance. ITH can be a great challenge when providing therapy. Profiling the composition of a tumor cell population could play a significant role in personalizing the therapeutic options for individual patients. With the recent advances in precision medicine using next-generation sequencing, characterization of ITH can allow for a better understanding of tumorigenesis and the development of personalized therapeutic strategies for cancer patients.

Both genotypic and phenotypic characterization of ITH could help improve personalized cancer treatment strategies^4^. On the genotypic level, the identification of somatic mutations is critical to detect the specific genomic abnormalities of cancer patients. Continuous accumulation of mutated cells during tumor progression promotes the generation of clusters or subpopulations of tumor cells. Thus, ITH, genetic diversity within individual tumors, is currently one of the challenging issues in cancer research^5^. Precise detection of actionable variants is important for molecular targeted therapeutics. ITH data are also valuable to identify factors associated with drug resistance and tumor recurrence^6^.

Genome sequencing has enabled the determination of tumor characteristics in diverse cancer types, including pancreatic cancer^7^, breast cancer^8^, renal cell carcinoma^9^, secondary acute myeloid leukemia^10^, primary glioblastoma^11^, and lung cancer^12^. High-throughput sequencing technologies, such as whole-exome sequencing (WES), have been used to measure ITH, identifying clonal architecture, evolution patterns, and drug resistance mechanisms of tumors^9,13–15^. From WES, somatic mutations are identified, and the allele frequency of each mutation is quantified. However, sequencing from bulk tissue yields only an average of the mixed subpopulations of cells^16^. Despite this limitation, subclones can be identified by diverse computational approaches using WES of bulk tissue^17^.

Recently, a cancer panel based on high-depth next-generation sequencing technology was used to accurately identify mutations in numerous oncogenes^18–20^. Its advantages include high sensitivity of detection for identifying rare mutations as well as minor alleles with lower depth. To measure the heterogeneity of mutations within a limited set of oncogenes in a clinical setting, a cancer panel is needed; however, this approach for ITH profiling has not yet been fully investigated. Therefore, it would be useful to implement and validate this method to profile heterogeneity in cancers, in order to better-inform treatment decisions.

In this study, we measured tumor heterogeneity (TH) via deep sequencing of a customized set of genes and used it to calculate a TH index. We further validated the deep sequencing results using heterogeneity measurements from WES. In addition, we tried to identify the clinical significance of the TH index for the diagnosis and treatment of cancer patients.

## Results

### Use of cancer panel sequencing data to measure tumor heterogeneity

**Figure 1:**
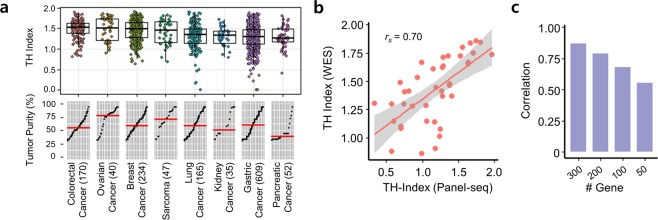
Tumor heterogeneity measurement from cancer panel sequencing. (**a**) Tumor heterogeneity (TH) indices and tumor purities for 1,352 tumors from 8 types of cancer. Each point corresponds to a sample. Tumor purities were sorted and the red horizontal line indicates the median value. (**b**) Scatter plot between TH indices measured using either cancer panel sequencing or whole-exome sequencing. (**c**) Correlation between TH indices calculated using 381 genes and those indices using a subset of those genes.

We measured the TH index of 1,352 tumor samples from 8 cancer types using targeted panel sequencing data (Fig. 1a, Table S1). The TH index was calculated using Shannon’s index^21^ with variant allele frequencies (VAFs) of mutated loci in 381 cancer-related genes. First, we checked that the TH indices from the cancer panel sequencing were consistent with those measured using WES by comparing data generated from the same 40 breast cancer patient samples. Although we performed targeted sequencing on approximately 1.8–2.0% of total genes, TH indices from the WES data correlated highly with the TH indices from the cancer panel sequencing data (Spearman *r*_*s*_ = 0.70, *p* < 0.001) (Fig. 1b).

Next, we examined whether the TH index measurement was affected by decreasing the number of genes on the panel. We down-sampled the sequenced regions by selecting a smaller number of genes to assess the minimal dimension of panel sequencing for tumor heterogeneity measurement (Table S2). When we decreased the number of gene regions for TH index estimation, the TH indices did not correlate well with the original value from the WES data (Fig. 1c and Fig. S1). The TH indices obtained from 300 genes were quite similar to those obtained using 381 genes (*r*_*s*_ = 0.87); however, the correlation decreased markedly when we used 50 genes (*r*_*s*_ = 0.50). This indicates that when fewer genes were analyzed, the TH index of tumor samples could have been calculated incorrectly.

We also investigated the landscape of TH indices across cancer types, comparing publicly available WES and targeted panel sequencing data. We measured the TH index for 8,578 tumors of 21 cancer types (Table S3) listed in The Cancer Genome Atlas (TCGA). Tumor heterogeneity implies that subclones may also exist within a tumor. The TH index correlated with the number of subclones in each sample (Fig. S2), wherein the tumors with more subclones had a higher TH index (Lower vs. higher clonal samples: *p* < 2.2 × 10^−16^). The TH indices of individual tumors in each cancer type were distributed widely (Fig. S3a), and uterine carcinosarcoma (UCS), rectal adenocarcinoma (READ), colon adenocarcinoma (COAD), and ovarian cancer (OV) were observed to be more heterogeneous than other tumor types. By contrast, kidney renal clear cell carcinoma (KIRC) and thyroid carcinoma (THCA) were relatively homogeneous. The pattern of tumor heterogeneity did not match tumor purity and the number of mutations (correlation with the medians of cancer types: *r*_*s*_ = 0.06, *p* = 0.79 and *r*_*s*_ = 0.02, *p* = 0.91, respectively) (Fig. S3b,c); that is, a cancer type with high heterogeneity was not caused by having a sample with a high percentage of tumor content or mutation number. However, within a cancer type, the TH indices were correlated positively with tumor purity, while the correlation with the number of mutations was not always positive (Table S4). This indicates that the TH index was not mainly determined by the number of mutations.

The correlation between the TH index and the overall survival of patients was significant in several cancer types, such as OV, adrenocortical carcinoma (ACC), and head and neck squamous cell carcinoma (HNSC), but not significant for READ (Fig. S3d). In addition, there was a pattern of increasing heterogeneity according to the pathological stage in ACC, HNSC, lung adenocarcinoma (LUAD), and lung squamous cell carcinoma (Fig. S3e). The degree of clonal diversity might increase with increasing tumor stage as cells become more invasive. For some types of cancer, we could not present patterns of heterogeneity since there was no information available regarding their pathological stages.

### Correlation of tumor heterogeneity with clinical outcome of refractory colorectal cancers

Colorectal cancer (CRC) tumor samples had high TH indices in the TCGA (Fig. S3a) and the in-house cancer panel sequencing data (Fig. 1a). The purity of the TCGA COAD and READ samples was higher than in our CRC dataset. CRC includes major subtypes of COAD and READ. While TCGA samples are well-curated sets with high purity for the purpose of study, in a clinical setting, quality control criteria for samples differ. Thus, it is very possible that the pattern of tumor heterogeneity did not match the tumor purity across cancer types.

To validate advanced cases, we analyzed an additional 304 formalin-fixed paraffin-embedded (FFPE) tissue samples from colorectal cancer patients treated with palliative chemotherapy (Table 1). Among these CRC cases, half of the patients were classified as stage IV, having synchronous metastases at initial diagnosis. Although the remaining patients were stage I to III at initial diagnosis (without distant metastases) and had received curative surgery, most of them eventually suffered recurrence or resistance to conventional chemotherapy.

**Table 1:** Demographic and clinical information of colorectal cancer patients.

Characteristics	Number of patients (n = 304)
Age, median (years)	54.5
Gender, n (%)	
Male	186 (61.2%)
Female	118 (38.8%)
CEA, n (%)	
<5 ng/ml	173 (56.9%)
≥5 ng/ml	120 (39.5%)
Unknown	11 (3.6%)
Location of primary tumor, n (%)	
Colon	201 (66.1%)
Rectum	103 (33.9%)
Stage, n (%)	
I	10 (3.3%)
II	30 (9.9%)
III	125 (41.1%)
IV	139 (45.7%)
Cell type, n (%)	
WD/MD	253 (83.2%)
PD/MUC/SRC	51 (16.8%)
Lymphatic invasion, n (%)	
Yes	170 (55.9%)
No	124 (40.8%)
Unknown	10 (3.3%)
Vascular invasion, n (%)	
Yes	135 (44.4%)
No	158 (52.0%)
Unknown	11 (3.6%)
Perineural invation, n (%)	
Yes	117 (38.5%)
No	171 (56.2%)
Unknown	16 (5.3%)
Tumor budding, n (%)	
Yes	189 (62.2%)
No	73 (24.0%)
Unknown	42 (13.8%)
Adjuvant treatment, n (%)	
Yes	294 (96.7%)
No	10 (3.3%)

**Figure 2:**
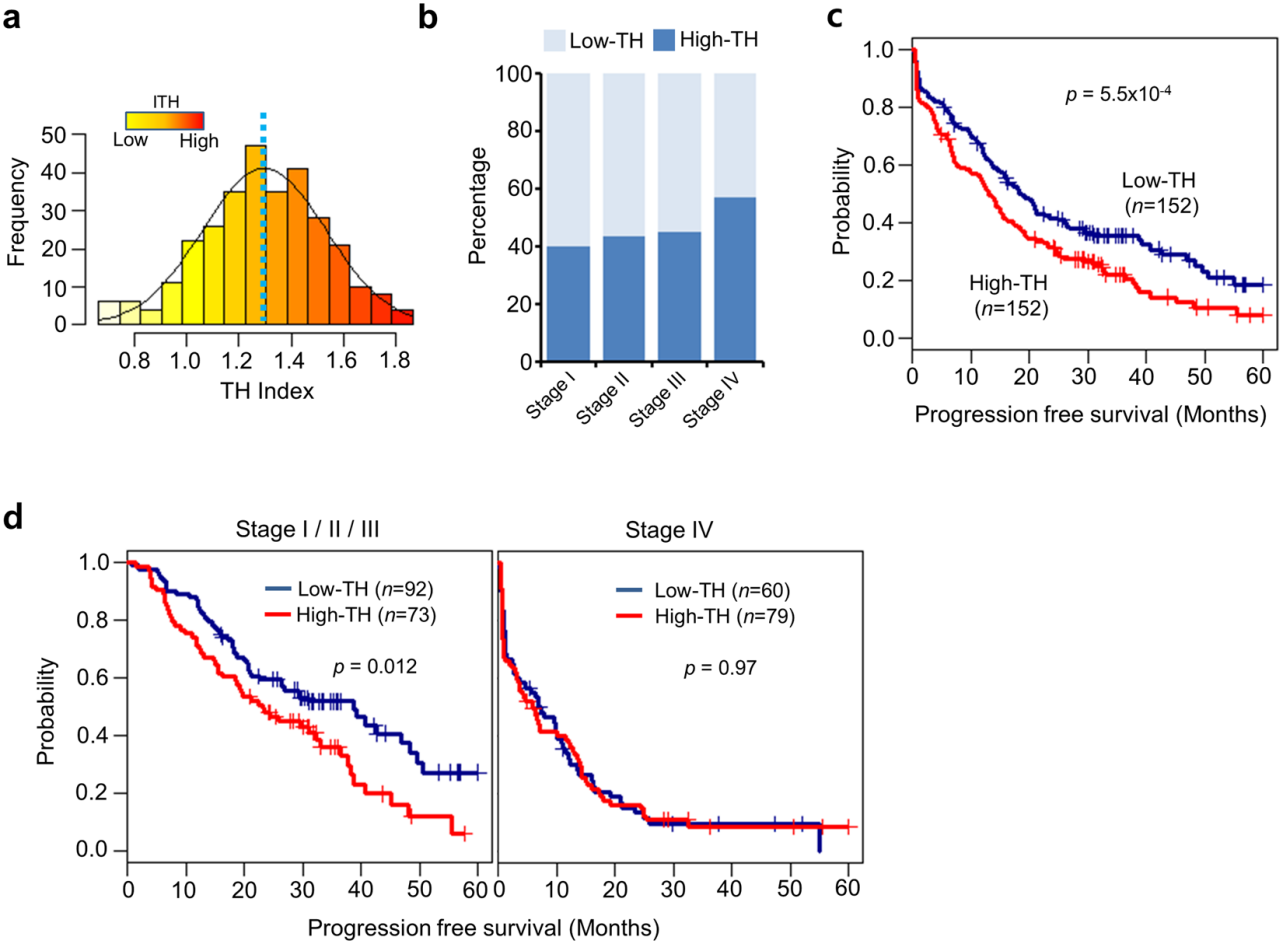
Tumor heterogeneity measurement from cancer panel sequencing of colorectal cancer. (**a**) Histogram and distribution of tumor heterogeneity (TH) indices. (**b**) Survival plot comparing patients with high and low tumor heterogeneity. (**c**) Differences in TH indices based on pathological stage (I, II, III, and IV). (**d**) Progression-free survival curves according to TH in stages I/II/III, or IV.

The shape of the TH index distribution curve (average 1.30 ± 0.24) resembled that of a normal distribution (Fig. 2a and Table S5). We determined the cutoff between high and low heterogeneity (TH index = 1.30) based on the average TH. We examined whether the heterogeneity of this cohort of CRC patients was associated with clinical outcome. There was a significantly higher proportion of high-TH cancers at more advanced stages, with 40.0% in stage I, 43.3% in stage II, 44.8% in stage III, and 56.8% in stage IV (linear-by-linear association test, *p* = 0.046) (Fig. 2b). In survival analysis for TH indices, there was a significant difference between the low-TH (*n* = 152) and high-TH (*n* = 152) groups with regard to progression-free survival (*p* = 5.5 × 10^−4^) (Fig. 2c). This suggests that higher heterogeneity could be considered a bad prognostic factor for recurrence in CRC patients. However, this result may be due to the higher number of patients with stage IV cancers in the high-TH group (Fig. 2b). Thus, subgroup analyses were performed according to the presence of metastases (Fig. 2d). In those patients without metastasis (stages I to III), we observed a significant difference in progression-free survival according to heterogeneity (*p* = 0.012), suggesting that TH might be a determining factor for recurrence or metastasis in CRC patients with curative resection. However, there was no difference in progression-free survival based on heterogeneity in patients with metastasis (stage IV) (*p* = 0.97). This may be because the tumors in stage IV patients had already metastasized, and thus many factors other than heterogeneity might affect their survival when compared with stage I to III patients. In addition, we tested the relationship between survival and TH index for patients with other cancer types, including breast and gastric cancers. We observed similar results for breast cancer: patients with high TH indices had poor progression-free survival (*p* = 0.034) (Fig. S4). However, the TH indices for gastric cancers among other available clinical data did not correlate with survival patterns.

### Improved prediction of clinical outcome using combined genetic and clinical information

**Figure 3:**
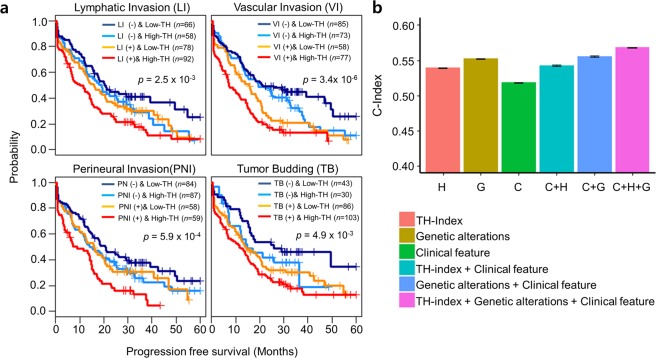
Integration of genetic and clinical information. (**a**) Survival plots combining tumor heterogeneity (TH) with clinical features, including lymphatic invasion (LI), vascular invasion (VI), perineural invasion (PNI), and tumor budding (TB). Kaplan-Meier curves with patients grouped by clinical features and TH index (−, low; −, high; +, low; +, high). The log-rank test measured between two groups (a clinical feature (−) & low TH index, and a clinical feature (+) & high TH index). (**b**) Powers of risk prediction (C-index) integrated with a genomic and/or a clinical feature. Genetic alterations include *APC*, *KRAS*, and *TP53*.

Several clinical features, including lymphatic invasion (LI), vascular invasion (VI), perineural invasion (PNI), and tumor budding (TB), correlate with poor clinical outcomes for colorectal cancer patients^22–24^. In our cohort, LI, VI, PNI, and TB also correlated significantly with poor clinical outcomes. We examined whether adding the TH index as a prognostic marker would increase the accuracy of measuring prognosis (Fig. 3a). The TH index combined with LI, VI, PNI, and TB revealed distinct survival differences among patient groups (*p* = 2.5 × 10^−3^, 3.4 × 10^−6^, 5.9 × 10^−4^, and 4.9 × 10^−3^, respectively). When the TH index was integrated with the genomic and/or clinical variables, the power of risk prediction (C-index) appeared to improve (*p* < 2.2 × 10^−16^, *t*-test between with TH index and without TH index) (Fig. 3b). The combination of TH index, genetic alterations (in the *APC*, *KRAS*, and *TP53* genes), and clinical features exhibited a higher power of risk prediction than the use of any of these factors individually.

## Discussion

In this study, using a cancer panel designed to cover the genomic regions of frequently mutated loci, we demonstrated the clinical utility of measuring TH, particularly for CRC. The strength of our study is that we proved clearly that a limited set of cancer-related genes is suitable to assess the degree of TH. We demonstrated that TH measurements from a cancer gene panel correspond well with those from WES and reflect the degree of clonality accurately. Furthermore, this measurement can help predict metastatic potential, cancer invasion, and patient outcomes in combination with other prognostic markers.

Many studies have investigated TH in several types of cancer, including LUAD, renal cell carcinoma, and chronic lymphocytic leukemia, based on observations of the molecular profiles of copy number alterations and mutations^12,13,25,26^. These studies usually measured tumor heterogeneity using WES data^16,27,28^. We addressed the challenge of using reduced genomic information to determine TH. Clinically actionable information could be identified using a handful of druggable genes that are used widely in the clinic. We could expand the utility of cancer panel sequencing to acquire additional prognostic information on patients.

The integration of TH and clinical features has useful clinical implications for treatments targeting cancer progression and drug response. The genomic heterogeneity caused by continuous mutational processes influences the clinical outcome greatly for patients. Specifically, metastatic progression is a multistep process involving phenotypic changes of primary tumor cells caused by genetic and epigenetic alterations that facilitate dissemination and tissue invasion^29,30^. Although there are many reports concerning proto-oncogenes and tumor suppressor genes, less is known about the molecular events responsible for metastatic progression. We suggest that a more thorough understanding of ITH would be helpful to develop treatment strategies using this concept as a progression marker of metastatic and recurrent tumors. In the present study, we confirmed the clinical usefulness of ITH using cancer panel sequencing and by applying our results to patients with metastatic or recurrent CRC.

In this study, we focused on a comprehensive determination of TH via targeted panel sequencing analysis in cancer patients. We demonstrated that the proposed method of measuring TH index was highly clinically relevant. In the near future, this could help clinicians determine if an adjuvant therapy could be helpful after curative resections. In conclusion, our analytical approach could help to measure the heterogeneity of each tumor and will contribute to the development of improved personalized treatments for cancer patients.

## Methods

### Patients

All methods were performed in accordance with the relevant guidelines and regulations. This study was performed by collecting retrospective data. The Institutional Review Board (IRB) of the Samsung Medical Center approved this study. Among all the patients, written informed consent was obtained unless it was practically impossible, in which case the IRB waived their consent. A total of 1,352 samples were obtained from cancer patients at the Samsung Medical Center from 2014 to 2016. All patients underwent surgical resection for a primary tumor and confirmed carcinoma from different origins, such as the colorectum, ovary, breast, lung, kidney, stomach, and pancreas. We had clinical information for 304 Korean patients diagnosed with CRC; their characteristics are summarized in Table 1.

### Isolation of genomic DNA and quality control

Patient tissue DNA was obtained by FFPE deparaffinization and a Maxwell 16 CSC DNA FFPE kit (Promega, Madison, WI, USA). The purity, amount, and median size of extracted DNA were measured using a Nanodrop 8000 UV-Vis spectrometer (Thermo Scientific Inc., Wilmington, DE, USA), Qubit 2.0 fluorometer (Life Technologies Inc., Grand Island, NY, USA), and a 2200 TapeStation Instrument (Agilent Technologies, Santa Clara, CA, USA). In addition, the ΔCt value was determined using real-time PCR (Agilent Technologies) with a Mx3005p instrument (Agilent Technologies, USA), FFPE QC kit (Illumina, cat no/ WG-321–1001), and Brilliant Ultra-Fast SYBR Green qPCR (Agilent Technologies, cat no. 600882). DNA was only sequenced if it met our quality criteria: (i) purity: absorption ratio (260 nm/280 nm) >1.8, 260 nm/230 nm >1.8; (ii) total amount >250 ng; (iii) degradation: ΔCt value <2.0, or DNA median size >0.35 kb.

### Sequencing by customized cancer panel

To obtain cancer panel sequencing data, CancerSCAN probes were designed to enrich the exons of 381 genes. In brief, the cancer panel sequencing data were generated by DNA shearing, library construction, and sequencing on a HiSeq 2500 sequencing platform (Illumina, San Diego, CA, USA) according to manufacturer’s protocol. Libraries were constructed by end repair, A-tailing, paired-end adaptor ligation, amplification, hybridization, indexing, and enrichment using a SureSelectXT reagent kit, HSQ (Agilent Technologies). The sequencing reactions were performed with the 100-bp paired-end mode of the TruSeq Rapid PE Cluster kit and TruSeq Rapid SBS kit (lllumina).

### Targeted exome-sequencing analysis

Sequence reads were mapped to the human genome (hg19; downloaded from http://genome.ucsc.edu) using the program Burrows-Wheeler Aligner^31^. Duplicate reads were removed using Picard (http://picard.sourceforge.net/) and Samtools (http://samtools.sourceforge.net/). Single nucleotide variants were identified using MuTect 1.1.44 (https://github.com/broadinstitute/mutect) and LoFreq 0.6.15^32^, with default parameters, and the results from the two callers were merged. For panel sequencing, an in-house algorithm was used to filter out likely false-positive variants^33^. Suspected germline variants based on the allele frequency of normal samples were filtered out. Public databases including dbSNP138, COSMIC, and TCGA as well as in-house single nucleotide polymorphism (SNP) databases were used for the filtering, and ANNOVAR (http://www.openbioinformatics.org/annovar/) was used to annotate the detected variants.

### Measurement of heterogeneity

Heterogeneity measurement was based on Shannon’s index, which is a popular index to measure species diversity^21,34^. For the VAFs of mutated loci in each tumor, VAFs ∈ [0, 100] were obtained, the VAFs were assigned to *i*-th of *N* bins, and then Shannon’s index (*H*’) was calculated using the probability distribution (*p*_*i*_) belonging to the bins (*i* = 1, …, *N*):

$$H^{\prime} =-\,\sum _{i=1}^{N}{p}_{i}\,\mathrm{ln}\,{p}_{i}$$

here, we set the bin size to 10, yielding enough information to represent the distribution for proportions of VAFs.

### Purity estimation

Computational estimation of tumor purity using panel sequencing data is more challenging than from exome or whole-genome sequencing data because the limited DNA regions in panel sequencing contain insufficient genomic alterations for its calculation. About half of the samples were ideal for purity estimation. To estimate tumor purity, we first identified copy-neutral regions. In those copy-neutral regions, the minor allele frequencies of known SNPs were near 0.5. Considering only those SNPs, their read densities were shown to be the most prominent peak in the read coverage distribution observed across the SNPs. Regions of copy number gain and loss were inferred based on their adjusted coverage values, considering the copy number-neutral regions. Once the copy-neutral, gain, and loss regions were clarified, the following formula could be used to compute the purity (the maximum of the purity values estimated at multiple positions was used) by measuring the alternative allele frequency (AAF):

$${\rm{AAF}}=({P}^{\ast }Y+(1-P))/({P}^{\ast }X+2(1-P)),$$

where *X* and *Y* represent the numbers of all and alternative alleles at each group of SNP clusters in the tumor, respectively. *P* is the tumor purity ranging from 0 to 1. More details are available in a separate manuscript^33^.

### TCGA data

Clonality information was obtained from previous results measured using PyClone and EXPANDS tools^28^. Purity information for each cancer was taken from a previous Pan-cancer study^35^. Somatic mutations and their VAF information were obtained from Broad Genome Data Analysis Center Firehose (https://gdac.broadinstitute.org/).

### Survival analysis

The significance of the selected genes for clinical outcome was represented using Kaplan-Meier survival analysis using the R survival package (http://CRAN.R-project.org/package=survival). The powers of risk prediction were measured using the coxph function in the survival package. We performed the analysis with 500 iterations of subsets that were selected by random sampling.

## Supplementary information

Supplementary Information

## Data Availability

The data sets generated and/or analysed during the current study are not publicly available due to medical confidentiality. They are only available from a formal data access committee at the Samsung Medical Center, upon reasonable request.
