# Supplementary material for: Intratumor heterogeneity inferred from targeted deep sequencing as a prognostic indicator
Source: Sci Rep. 2019 Mar 14;9:4542. doi: 10.1038/s41598-019-41098-0 (PMC6418103; doi:10.1038/s41598-019-41098-0)
Supplement: Supplementary file 1 — Supplementary Information [file 41598_2019_41098_MOESM1_ESM.pdf]

Intratumor heterogeneity inferred from targeted deep sequencing as a prognostic indicator

Bo Young Oh[1], Hyun-Tae Shin[2], Jae Won Yun[2], Kyu-Tae Kim[3], Jinho Kim[2], Joon Seol Bae[2], Yong Beom Cho[4,5], Woo Yong Lee[4,5], Seong Hyeon Yun[4], Yoon Ah Park[4], Yeon Hee Park[6], Young-Hyuck Im[6], Jeeyun Lee[6], Je-Gun Joung[2*], Hee Cheol Kim[4*], Woong-Yang Park[2,5,7,8*]

Author's affiliations:

[1] Department of Colorectal Surgery, Hallym University Sacred Heart Hospital, Hallym University College of Medicine, Anyang, Korea

[2]Samsung Genome Institute, Samsung Medical Center, Seoul, Korea

[3]New York Genome Center, New York, NY, USA

[4]Department of Surgery, Samsung Medical Center, Sungkyunkwan University School of Medicine, Seoul, Korea

[5]Department of Health Sciences and Technology, Samsung Advanced Institute of Science and Health Technology, Sungkyunkwan University, Seoul, Korea

[6]Division of Hematology and Oncology, Department of Medicine, Samsung Medical Center, Sungkyunkwan University School of Medicine, Seoul, Korea

[7]Department of Molecular Cell Biology, Sungkyunkwan University School of Medicine, Seoul, Korea

[8]GENINUS Inc., Seoul, Korea

Supplementary Information

Figure S1

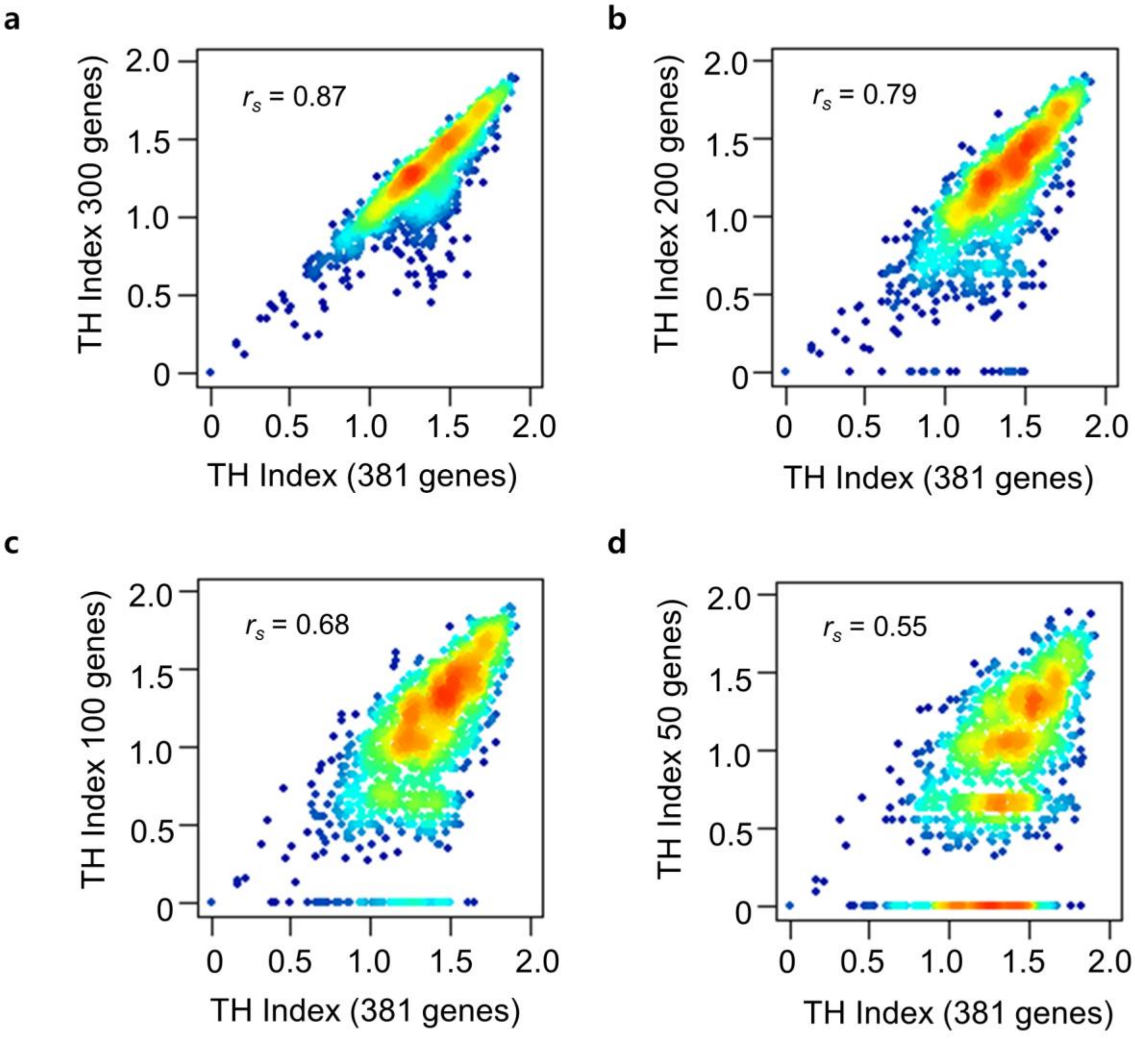

Figure S1. Bias of the tumor heterogeneity index measurement according to the decrease in number of observed panel genes. (**a**–**d**) With the decrease in the number of genes in the panel, the similarity (r_s, Spearman's rank correlation coefficient) of tumor heterogeneity indices was calculated between genes in the subset (n = 50, 100, 200, and 300) and those in the whole set (n = 381).

Figure S2

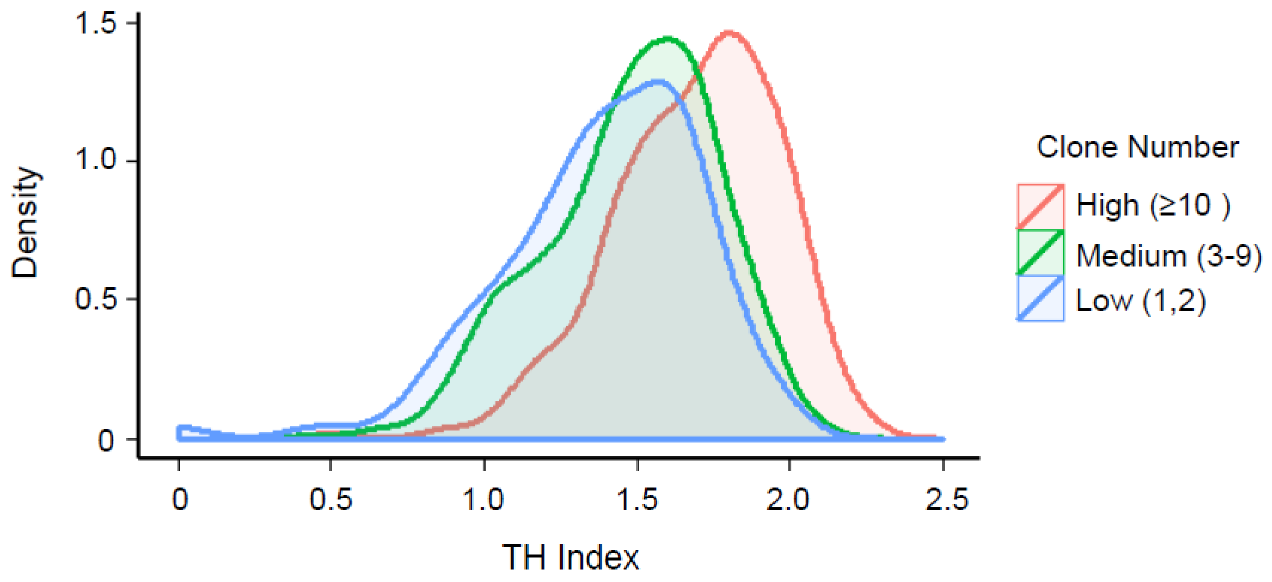

Figure S2. Correlation between the numbers of subclones and heterogeneity indices.

Distributions of tumor heterogeneity indices among three groups of samples with a high (≥10), medium (3–9), and low (1, 2) number of subclones that were significantly different from one another (Low vs. High: $p < 2.2 \times 10^{-16}$; Low vs. Medium: $p < 4.7 \times 10^{-4}$; and Medium vs. High: $p < 2.2 \times 10^{-16}$, two sample *t*-test).

Figure S3

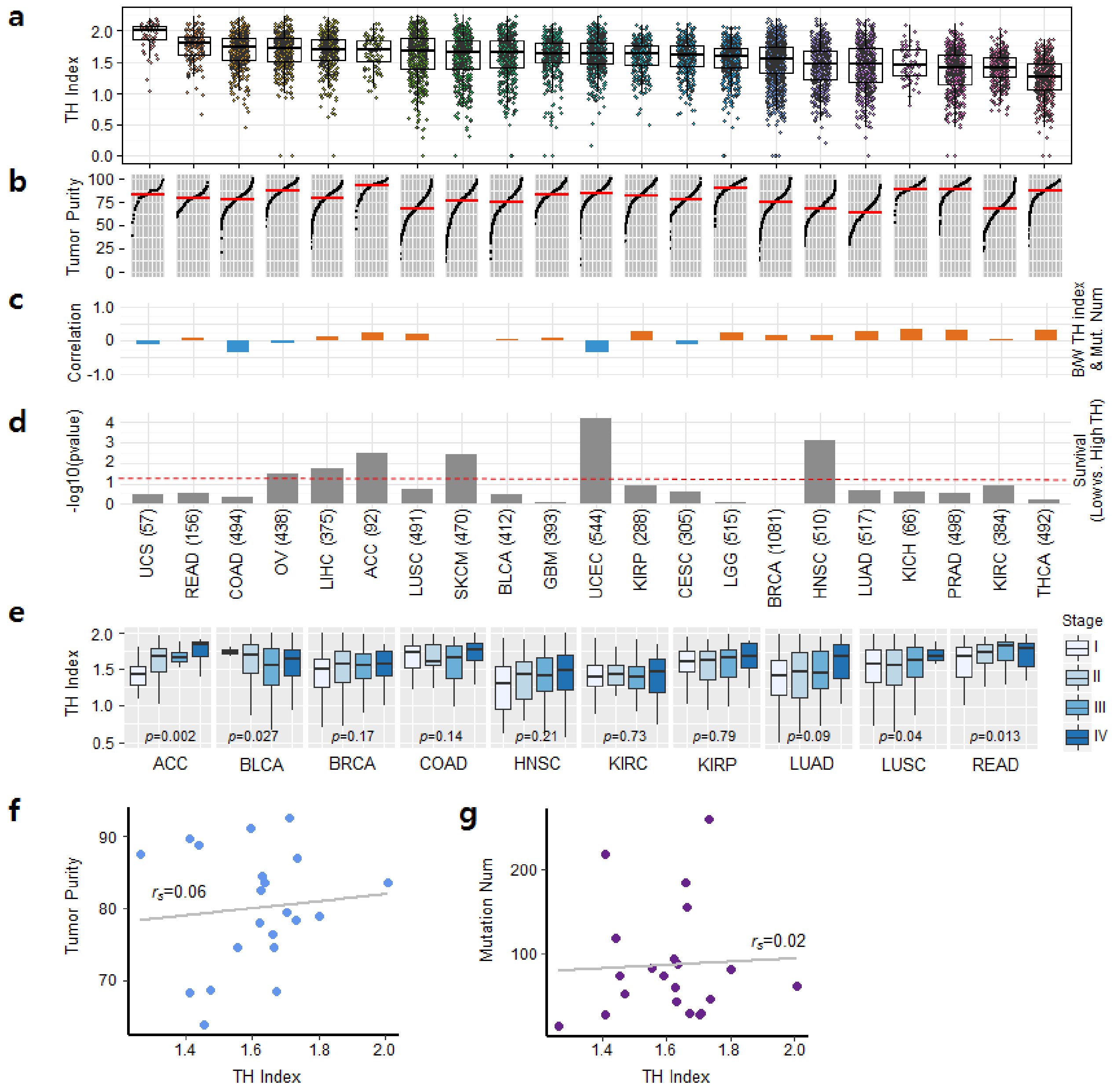

Figure S3. The landscape of tumor heterogeneity from whole-exome sequencing in the TCGA data.

(**a**) Tumor heterogeneity (TH) indices measured from 8,578 tumors of 21 types of cancer in The Cancer Genome Atlas (TCGA). Full disease names of the cohorts are shown in **Table S4**. The distributions of TH indices are shown for each type of cancer. Each TH index was calculated on the basis of the distribution of variant allele frequencies (VAFs) of mutated loci, using whole-exome sequencing data. (**b**) Tumor purities are shown for each type of cancer. (**c**) Correlations between TH indices and the number of mutations. (**d**) Survival significance between two groups with high and low

heterogeneity for each type of cancer. Each bar represents –log10 (survival p-value). Two groups were determined by the mean cut-off. (**e**) Difference in TH indices according to pathological stage. (**f**) Scatter plot of the correlation between the median TH index and the median tumor purity for each cancer type. (**g**) Scatter plot of the correlation between the median TH index and the median mutation number for each cancer type.

Figure S4

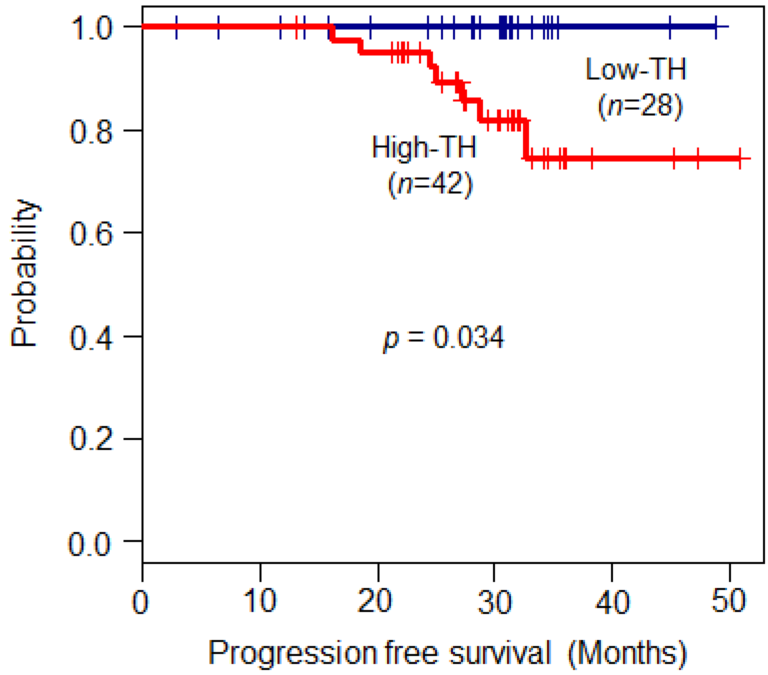

Figure S4. Survival plot for breast cancer. Plot of progression-free survival for breast cancer.

Table S1. The list of tumor heterogeneity (TH) index measured in 8 different types of cancer.

Num	ID	SNV num	TH index	Specimen	Diagnosis_confirm	GC	Q30	Dup.Rate	Coverage	OnTarget Rate100	Purity (%)
1	15_00262_DT_CS	34	1.151	Tissue DNA	Colorectal cancer	45	92.1	12.86	1138.8	0.987	NA
2	15_00263_DT_CS	69	1.246	Tissue DNA	Colorectal cancer	45	92	11.82	1174.8	0.987	NA
3	15_00264_DT_CS	17	1.122	Tissue DNA	Colorectal cancer	45	92.3	13.9	1237.4	0.988	NA
4	15_00265_DT_CS	105	1.688	Tissue DNA	Colorectal cancer	45	92	12.32	1380.1	0.988	NA
5	15_00280_DP_CS	121	1.410	FFPE DNA	Colorectal cancer	47.5	93.8	17.18	932.3	0.990	27.3
6	15_00281_DP_CS	26	1.043	FFPE DNA	Colorectal cancer	49	93.3	31.4	698	0.991	NA
7	15_00282_DP_CS	59	1.515	FFPE DNA	Colorectal cancer	49	93.4	31.66	726.3	0.991	54.0
8	15_00288_DP_CS	75	1.545	FFPE DNA	Colorectal cancer	48.5	93.1	14.3	907.5	0.990	63.0
9	15_00405_DP_CS	66	1.410	FFPE DNA	Colorectal cancer	50	93	49.39	475.4	0.989	100.0
10	15_00408_DP_CS	46	1.558	FFPE DNA	Colorectal cancer	49	92.3	18.23	937.5	0.991	100.0
11	15_00409_DP_CS	160	1.427	FFPE DNA	Colorectal cancer	51	91.9	33.41	688.3	0.991	100.0
12	15_00411_DP_CS	112	1.659	FFPE DNA	Colorectal cancer	48.5	92.5	15.99	890.6	0.989	100.0
13	15_00415_DT_CS	51	1.550	Tissue DNA	Colorectal cancer	46	93.2	16.08	934.6	0.988	NA
14	15_00416_DT_CS	46	1.614	Tissue DNA	Colorectal cancer	45.5	93.2	16.98	971.4	0.988	NA
15	15_00420_DT_CS	47	1.436	Tissue DNA	Colorectal cancer	45.5	93.2	19.19	831.1	0.986	NA
16	15_00421_DT_CS	89	1.760	Tissue DNA	Colorectal cancer	46	93.2	15.89	924.8	0.987	NA
17	15_00431_DP_CS	69	1.439	FFPE DNA	Colorectal cancer	49	92.6	31.88	876.4	0.991	100.0
18	15_00432_DP_CS	42	0.846	FFPE DNA	Colorectal cancer	48	92.7	12.9	1081.3	0.990	32.6
19	15_00437_DT_CS	49	1.659	Tissue DNA	Colorectal cancer	45	93.2	14.53	1012.8	0.987	32.7
20	15_00440_DP_CS	86	1.689	FFPE DNA	Colorectal cancer	49	92.5	12.14	1030.7	0.991	82.4
21	D_15_00797_DT_CS	92	1.394	Tissue DNA	Colorectal cancer	45	93.5	11.72	977.5	0.986	39.7
22	D_15_00798_DT_CS	72	1.518	Tissue DNA	Colorectal cancer	45	93.2	11.72	944.6	0.987	24.9
23	D_15_00805_DP_CS	37	1.335	FFPE DNA	Colorectal cancer	47	92.1	16.9	935.7	0.990	27.6
24	D_15_00812_DP_CS	99	1.740	FFPE DNA	Colorectal cancer	50	91.3	20.5	879.6	0.990	68.4
25	D_15_00817_DP_CS	166	1.540	FFPE DNA	Colorectal cancer	52	91.2	32.96	682.9	0.987	100.0
26	D_15_00869_DP_CS	176	1.826	FFPE DNA	Colorectal cancer	47	92	20.86	905.9	0.990	94.1
27	D_15_00873_DT_CS	60	1.424	Tissue DNA	Colorectal cancer	46	92.4	30.55	761.8	0.987	63.2
28	D_15_00905_TS_CS	116	1.572	Frozen Tissue	Colorectal cancer	45	91	13.26	956	0.983	63.0
29	D_15_00906_TS_CS	66	1.184	Frozen Tissue	Colorectal cancer	45	90.6	15.38	968.2	0.984	59.2
30	D_15_00907_TS_CS	80	1.351	Frozen Tissue	Colorectal cancer	45	91.3	13.68	986.8	0.984	61.1
31	D_15_00908_TS_CS	132	1.408	Frozen Tissue	Colorectal cancer	45	91	13.44	975.5	0.985	38.1
32	D_15_00909_TS_CS	82	1.681	Frozen Tissue	Colorectal cancer	45	91.3	15.53	985.3	0.984	100.0
33	D_15_00910_TS_CS	178	1.755	Frozen Tissue	Colorectal cancer	45.5	91.4	12.95	1031.6	0.985	66.7
34	D_15_00911_TS_CS	153	1.399	Frozen Tissue	Colorectal cancer	45	90.9	14.48	1034.6	0.985	70.3
35	D_15_00965_FP_CS	11	1.241	FFPE Block	Colorectal cancer	52	91.1	27.05	916	0.991	22.2
36	D_15_00966_DT_CS	64	1.548	Tissue DNA	Colorectal cancer	45	93.7	15.66	944.4	0.987	NA
37	D_15_00968_DT_CS	141	1.684	Tissue DNA	Colorectal cancer	45	93.8	14.97	1002.8	0.987	56.4
38	D_15_00969_DT_CS	92	1.482	Tissue DNA	Colorectal cancer	45.5	93.5	16.21	1044.1	0.990	NA
39	D_15_00970_DT_CS	129	1.681	Tissue DNA	Colorectal cancer	45	94.3	12.91	926.7	0.985	60.0
40	D_15_00971_DT_CS	66	1.389	Tissue DNA	Colorectal cancer	44.5	94.3	16.21	830.3	0.983	50.0
41	D_15_00972_DT_CS	63	1.406	Tissue DNA	Colorectal cancer	45	94.4	16.36	806	0.984	38.1
42	D_15_00973_DT_CS	127	1.634	Tissue DNA	Colorectal cancer	46	92.6	27.86	940.3	0.988	55.1
43	D_15_00974_DT_CS	84	1.472	Tissue DNA	Colorectal cancer	45	94.2	16.03	807.9	0.983	85.7
44	D_15_00975_DT_CS	158	1.681	Tissue DNA	Colorectal cancer	45	94.4	14.05	913.4	0.985	66.7
45	D_15_00976_DT_CS	156	1.813	Tissue DNA	Colorectal cancer	45	94.5	16.2	801.4	0.984	48.5
46	D_15_00977_DT_CS	178	1.602	Tissue DNA	Colorectal cancer	45	94.2	14.31	876.1	0.987	66.7
47	D_15_00978_DT_CS	118	1.705	Tissue DNA	Colorectal cancer	46	91.9	36.66	771.2	0.989	48.4
48	D_15_00979_DT_CS	84	1.709	Tissue DNA	Colorectal cancer	45	94.2	17.54	1045.5	0.986	55.1
49	D_15_00980_DT_CS	64	1.635	Tissue DNA	Colorectal cancer	46	92.2	37.97	771.4	0.989	56.4
50	D_15_00981_DT_CS	137	1.589	Tissue DNA	Colorectal cancer	44.5	94.2	13.31	816.2	0.984	50.0
51	D_15_00982_DT_CS	94	1.422	Tissue DNA	Colorectal cancer	46	93.9	12.16	924.2	0.987	50.0
52	D_15_00983_DT_CS	36	1.499	Tissue DNA	Colorectal cancer	46	92	24.57	869.1	0.988	22.2
53	D_15_00984_DT_CS	50	1.542	Tissue DNA	Colorectal cancer	45	94.7	10.72	946.4	0.986	32.6
54	D_15_00985_DT_CS	82	1.705	Tissue DNA	Colorectal cancer	46.5	92.6	44.84	676.4	0.984	48.4
55	D_15_00986_DT_CS	116	1.579	Tissue DNA	Colorectal cancer	46	92.6	28.17	865.4	0.988	50.5
56	D_15_00987_DT_CS	119	1.445	Tissue DNA	Colorectal cancer	46	92.2	30	814.3	0.987	63.2
57	D_15_00988_DT_CS	184	1.469	Tissue DNA	Colorectal cancer	46	91.8	35.27	993.4	0.989	52.4
58	D_15_00989_DT_CS	106	1.623	Tissue DNA	Colorectal cancer	46	92.2	25.81	861	0.988	100.0
59	D_15_00990_DT_CS	50	1.625	Tissue DNA	Colorectal cancer	45	94.6	15.66	787.8	0.984	36.0

60	D_15_00991_DT_CS	135	1.474	Tissue DNA	Colorectal cancer	45	94.3	16.24	869.3	0.984	48.5
61	D_15_00992_DT_CS	55	1.457	Tissue DNA	Colorectal cancer	46.5	92.2	21.91	1067.5	0.989	50.0
62	D_15_00993_DT_CS	81	1.418	Tissue DNA	Colorectal cancer	45	94.2	13.61	903.6	0.986	NA
63	D_15_00994_DT_CS	78	1.610	Tissue DNA	Colorectal cancer	47	93.3	34.52	883	0.979	70.3
64	D_15_00995_DT_CS	88	1.373	Tissue DNA	Colorectal cancer	44.5	94.2	14.28	816.8	0.984	36.0
65	D_15_00996_DT_CS	89	1.657	Tissue DNA	Colorectal cancer	47	93.2	39.43	750.3	0.981	77.8
66	D_15_00997_DT_CS	66	1.484	Tissue DNA	Colorectal cancer	45.5	93.3	16.31	1036.7	0.987	27.3
67	D_15_00998_DT_CS	35	1.604	Tissue DNA	Colorectal cancer	45	94	14.73	843.1	0.986	27.3
68	D_15_00999_DT_CS	107	1.576	Tissue DNA	Colorectal cancer	45	92.7	15.05	1009.1	0.987	50.5
69	D_15_01000_DT_CS	67	1.265	Tissue DNA	Colorectal cancer	45.5	93.2	16.16	1006.3	0.987	43.9
70	D_15_01001_DT_CS	175	1.661	Tissue DNA	Colorectal cancer	46	91.8	31.43	785.8	0.988	85.7
71	D_15_01002_DT_CS	158	1.639	Tissue DNA	Colorectal cancer	46	92.5	14.52	1022.3	0.988	94.1
72	D_15_01003_DT_CS	121	1.840	Tissue DNA	Colorectal cancer	45.5	93.2	14.79	977.8	0.986	64.0
73	D_15_01004_DT_CS	80	1.656	Tissue DNA	Colorectal cancer	46	91.5	26.66	802	0.989	45.0
74	D_15_01014_DP_CS	102	1.500	FFPE DNA	Colorectal cancer	50	91.2	63.87	382.9	0.989	100.0
75	D_15_01023_DT_CS	122	1.463	Tissue DNA	Colorectal cancer	46	92.7	21.65	852.8	0.989	77.8
76	D_15_01024_DT_CS	162	1.652	Tissue DNA	Colorectal cancer	46	93.1	15.34	1085.6	0.987	49.0
77	D_15_01093_DT_CS	141	1.699	Tissue DNA	Colorectal cancer	45.5	92.6	18.88	992.1	0.986	66.7
78	D_15_01152_DP_CS	132	1.813	FFPE DNA	Colorectal cancer	48	92.5	17.9	897.3	0.993	72.5
79	D_15_01158_DT_CS	45	1.559	Tissue DNA	Colorectal cancer	46	93.2	20.66	843.5	0.988	46.0
80	D_15_01160_DP_CS	97	1.607	FFPE DNA	Colorectal cancer	49	92.4	22.07	939	0.990	30.1
81	D_15_01162_DP_CS	106	1.830	FFPE DNA	Colorectal cancer	50	92.2	22.91	938.3	0.991	85.7
82	D_15_01169_DP_CS	58	1.296	FFPE DNA	Colorectal cancer	47.5	92.7	19.3	926.1	0.990	NA
83	D_15_01170_DP_CS	37	1.264	FFPE DNA	Colorectal cancer	47	91.6	23.51	1031.9	0.991	100.0
84	D_15_01171_DP_CS	43	1.417	FFPE DNA	Colorectal cancer	49	92.6	16.49	923.8	0.991	27.3
85	D_15_01176_DT_CS	143	1.055	Tissue DNA	Colorectal cancer	NA	NA	13.89	1160.5	0.986	100.0
86	D_15_01177_DT_CS	54	1.161	Tissue DNA	Colorectal cancer	NA	NA	19.73	1089.2	0.988	38.1
87	D_15_01179_DT_CS	78	1.173	Tissue DNA	Colorectal cancer	NA	NA	18.22	1132.4	0.987	68.0
88	D_15_01219_DT_CS	69	1.786	Tissue DNA	Colorectal cancer	46	91.7	15.4	964.4	0.989	40.0
89	D_15_01221_DP_CS	143	1.715	FFPE DNA	Colorectal cancer	48.5	92	21.69	1089.9	0.991	68.4
90	D_15_01222_DP_CS	166	1.750	FFPE DNA	Colorectal cancer	49	91.3	17.65	1113.3	0.991	57.2
91	D_15_01224_DP_CS	36	1.399	FFPE DNA	Colorectal cancer	51	91.4	45.89	615.4	0.990	NA
92	D_15_01227_DP_CS	121	1.697	FFPE DNA	Colorectal cancer	46.5	92.8	17.53	1098	0.987	50.5
93	D_15_01233_DT_CS	105	1.603	Tissue DNA	Colorectal cancer	46	92.3	14.02	950.8	0.989	55.1
94	D_15_01236_DT_CS	154	1.784	Tissue DNA	Colorectal cancer	46	92.2	19.23	1019.5	0.988	70.1
95	D_15_01264_DT_CS	141	1.784	Tissue DNA	Colorectal cancer	45.5	91.4	21.02	900.8	0.985	70.0
96	D_15_01265_DT_CS	126	1.354	Tissue DNA	Colorectal cancer	46	92.2	18.38	952.3	0.986	46.2
97	D_15_01419_DP_CS	101	1.534	FFPE DNA	Colorectal cancer	48	91.2	23.56	874.3	0.990	46.2
98	D_15_01421_DP_CS	64	1.542	FFPE DNA	Colorectal cancer	50	90.5	31.88	642.8	0.990	38.4
99	D_15_01427_DP_CS	60	1.713	FFPE DNA	Colorectal cancer	48.5	91.2	27.98	829.7	0.991	35.7
100	D_15_01442_DP_CS	89	1.288	FFPE DNA	Colorectal cancer	51	91.4	24.49	832.2	0.992	100.0
101	D_15_01458_DT_CS	57	1.106	Tissue DNA	Colorectal cancer	46	92.4	36.55	765.8	0.989	38.1
102	D_15_01459_DT_CS	140	1.429	Tissue DNA	Colorectal cancer	46	91.7	17.77	1050.6	0.988	52.9
103	D_15_01477_DP_CS	22	1.619	FFPE DNA	Colorectal cancer	47	92.1	15.17	860.1	0.990	27.4
104	D_15_01479_DP_CS	96	0.839	FFPE DNA	Colorectal cancer	48	92.2	19.75	838	0.991	82.0
105	D_15_01481_DP_CS	17	1.401	FFPE DNA	Colorectal cancer	48	92.2	16.8	854.2	0.991	NA
106	D_15_01483_DP_CS	27	1.491	FFPE DNA	Colorectal cancer	49	92.2	19.15	881	0.992	43.9
107	D_15_01485_DP_CS	24	1.471	FFPE DNA	Colorectal cancer	48	92.3	17.65	940.6	0.991	27.3
108	D_15_01668_TS_CS	126	1.582	Frozen Tissue	Colorectal cancer	45.5	92.5	26.54	757.3	0.986	63.0
109	D_15_01677_DT_CS	42	1.119	Tissue DNA	Colorectal cancer	45.5	93.5	14.64	1052.6	0.986	NA
110	D_15_01752_DP_CS	69	1.541	FFPE DNA	Colorectal cancer	49	92.5	13.94	878.2	0.990	32.9
111	D_15_01759_DP_CS	71	0.958	FFPE DNA	Colorectal cancer	49	91.2	17.97	989.2	0.991	24.4
112	D_15_01768_DT_CS	80	1.453	Tissue DNA	Colorectal cancer	45	92.8	20.97	978.8	0.986	50.0
113	D_15_01771_DT_CS	70	1.556	Tissue DNA	Colorectal cancer	46	92	12.25	996.5	0.985	42.0
114	D_15_01772_DT_CS	74	1.547	Tissue DNA	Colorectal cancer	45.5	92.6	10.09	1082.7	0.986	64.9
115	D_15_01786_TS_CS	24	1.376	Fresh Tissue	Colorectal cancer	46.5	91.1	11.58	1030.5	0.988	NA
116	D_15_01787_TS_CS	186	1.547	Fresh Tissue	Colorectal cancer	45	92.1	13.16	1150.4	0.988	81.0
117	D_15_01919_DT_CS	109	1.501	Tissue DNA	Colorectal cancer	45	92.2	16	1101.6	0.986	63.0
118	D_15_01920_DT_CS	48	0.904	Tissue DNA	Colorectal cancer	45	92.7	14.95	1032.5	0.984	NA
119	D_15_01962_DT_CS	148	1.719	Tissue DNA	Colorectal cancer	46	92.9	23.26	749.8	0.983	70.1
120	D_15_01971_DP_CS	84	1.631	FFPE DNA	Colorectal cancer	49.5	92.2	48.11	454.9	0.989	100.0
121	D_15_01975_DT_CS	6	1.242	Tissue DNA	Colorectal cancer	45.5	93.2	40.48	673	0.986	NA
122	D_15_01988_DP_CS	76	1.656	FFPE DNA	Colorectal cancer	50.5	93.2	32.24	699.6	0.990	52.9
123	D_15_01989_DP_CS	82	1.821	FFPE DNA	Colorectal cancer	50	93.8	17.81	786.4	0.988	66.7

124	D_15_01992_DP_CS	83	1.476	FFPE DNA	Colorectal cancer	49	93.8	27.92	748.8	0.989	76.5
125	D_15_01993_DP_CS	80	1.483	FFPE DNA	Colorectal cancer	49.5	94.2	34.12	674.9	0.989	51.5
126	D_15_02006_DT_CS	106	1.846	Tissue DNA	Colorectal cancer	45.5	95	28.26	695.4	0.981	59.6
127	D_15_02007_DT_CS	144	1.668	Tissue DNA	Colorectal cancer	46	94.5	22.48	711.3	0.985	50.7
128	D_15_02008_DT_CS	143	1.560	Tissue DNA	Colorectal cancer	46.5	94.2	28.3	734.6	0.988	77.8
129	D_15_02013_DP_CS	84	1.633	FFPE DNA	Colorectal cancer	49	89.9	14.53	907.7	0.990	54.6
130	D_15_02018_DP_CS	104	1.283	FFPE DNA	Colorectal cancer	49	91	12.79	1064.6	0.991	77.8
131	D_15_02019_DP_CS	108	1.564	FFPE DNA	Colorectal cancer	50	91.5	10.51	976.8	0.989	100.0
132	D_15_02043_DT_CS	74	1.495	Tissue DNA	Colorectal cancer	45	91.7	21.42	902.5	0.985	36.1
133	D_15_02044_DT_CS	25	1.200	Tissue DNA	Colorectal cancer	45	92.8	11.72	1062.1	0.985	38.1
134	D_15_02047_DT_CS	41	1.580	Tissue DNA	Colorectal cancer	45	92.8	8.86	1075.4	0.986	32.6
135	D_15_02050_DT_CS	73	1.385	Tissue DNA	Colorectal cancer	45.5	92.7	9.91	1057.3	0.986	32.6
136	D_15_02096_DP_CS	74	1.518	FFPE DNA	Colorectal cancer	48.5	91.7	15.78	1061.4	0.991	85.7
137	D_15_02101_DT_CS	10	1.055	Tissue DNA	Colorectal cancer	45	92.9	13.11	885.6	0.983	NA
138	D_15_02102_DT_CS	576	0.823	Tissue DNA	Colorectal cancer	45	92.8	12.96	1098.8	0.987	32.6
139	D_15_02103_DT_CS	141	1.804	Tissue DNA	Colorectal cancer	45	92.7	12.69	1172.1	0.986	100.0
140	D_15_02104_DT_CS	158	1.340	Tissue DNA	Colorectal cancer	47	93	34.86	677.3	0.987	40.0
141	D_15_02132_DP_CS	50	1.527	FFPE DNA	Colorectal cancer	50	92.5	56.93	346.9	0.977	54.6
142	D_15_02135_DP_CS	155	1.223	FFPE DNA	Colorectal cancer	50	91.5	32.87	783.2	0.992	100.0
143	D_15_02149_DP_CS	94	1.593	FFPE DNA	Colorectal cancer	NA	NA	45.37	740.6	NA	100.0
144	D_15_02154_DP_CS	75	1.475	FFPE DNA	Colorectal cancer	NA	NA	48.97	510	NA	100.0
145	D_15_02157_DP_CS	74	1.421	FFPE DNA	Colorectal cancer	NA	NA	53.89	541.8	NA	94.1
146	D_15_02158_DP_CS	60	1.134	FFPE DNA	Colorectal cancer	NA	NA	60.64	368.5	NA	26.4
147	D_15_02159_DP_CS	79	1.614	FFPE DNA	Colorectal cancer	NA	NA	63.4	366.6	NA	41.3
148	D_15_02367_DT_CS	47	1.697	Tissue DNA	Colorectal cancer	46	91.8	31.86	761.1	0.987	43.9
149	D_15_02396_DP_CS	65	1.311	FFPE DNA	Colorectal cancer	47.5	92.8	43.83	593.5	0.989	100.0
150	D_15_02411_DT_CS	78	1.474	Tissue DNA	Colorectal cancer	46	91.1	36.92	640	0.985	50.0
151	D_15_02594_DP_CS	39	1.284	FFPE DNA	Colorectal cancer	47.5	91.2	62.11	324.1	0.979	77.8
152	D_15_02597_DT_CS	90	1.416	Tissue DNA	Colorectal cancer	46	91.9	40.2	505	0.982	100.0
153	D_15_02605_DT_CS	96	1.215	Tissue DNA	Colorectal cancer	45.5	93.3	10.4	1223.1	0.981	94.1
154	D_15_02895_DT_CS	281	0.873	Tissue DNA	Colorectal cancer	46	91.6	31.29	615.3	0.986	22.2
155	D_15_02937_DT_CS	101	1.609	Tissue DNA	Colorectal cancer	46	91.5	32.93	712.3	0.986	100.0
156	D_15_04031_DT_CS	88	1.675	Tissue DNA	Colorectal cancer	46.5	94.5	37.74	601.6	0.985	66.2
157	D_15_04035_DP_CS	94	1.541	FFPE DNA	Colorectal cancer	49	93.9	30.35	685.6	0.989	50.0
158	D_15_04133_DP_CS	187	1.576	FFPE DNA	Colorectal cancer	48	94.2	34.18	597.1	0.986	88.9
159	D_15_04135_DP_CS	15	0.953	FFPE DNA	Colorectal cancer	49	94	40.31	537.6	0.988	NA
160	D_15_04136_DP_CS	76	1.301	FFPE DNA	Colorectal cancer	50	93.3	58.98	334	0.986	100.0
161	D_15_04146_DT_CS	18	1.040	Tissue DNA	Colorectal cancer	46	92.2	25.64	707.8	0.985	32.6
162	D_15_04472_DT_CS	89	1.500	Tissue DNA	Colorectal cancer	NA	NA	26.56	876	0.987	57.1
163	D_15_04484_DP_CS	471	0.812	FFPE DNA	Colorectal cancer	47.5	93.7	37.59	709.5	0.990	NA
164	D_15_04485_DP_CS	64	1.649	FFPE DNA	Colorectal cancer	49.5	92.5	27.86	816.2	0.991	55.1
165	D_15_04530_DT_CS	171	1.881	Tissue DNA	Colorectal cancer	46	92.8	14.46	1053.5	0.986	75.0
166	D_15_04838_DT_CS	156	0.957	Tissue DNA	Colorectal cancer	46	92.3	11.65	1079.6	0.988	NA
167	D_15_04886_DP_CS	88	1.430	FFPE DNA	Colorectal cancer	52	90	15.1	1105.5	0.991	100.0
168	D_15_04891_DP_CS	41	1.634	FFPE DNA	Colorectal cancer	49	92.2	36.52	724.7	0.990	31.5
169	D_15_04892_DP_CS	104	1.143	FFPE DNA	Colorectal cancer	49	92.8	15.87	1188.4	0.991	85.7
170	D_15_05059_DP_CS	110	1.410	FFPE DNA	Colorectal cancer	NA	NA	42.19	648.8	0.991	100.0
171	D_15_00924_TS_CS	19	1.441	Frozen Tissue	Ovary cancer	45	90.8	15.74	984.6	0.986	32.6
172	D_15_00925_TS_CS	72	1.040	Frozen Tissue	Ovary cancer	45	90.8	17.28	954.3	0.986	31.5
173	D_15_01284_TS_CS	84	1.764	Frozen Tissue	Ovary cancer	46	82.8	11.73	684.9	0.977	83.2
174	D_15_01285_TS_CS	125	1.715	Frozen Tissue	Ovary cancer	46	82.9	11.6	778.2	0.979	82.4
175	D_15_01286_TS_CS	96	1.802	Frozen Tissue	Ovary cancer	46	84.2	13.35	709.2	0.979	75.0
176	D_15_01287_TS_CS	138	1.820	Frozen Tissue	Ovary cancer	45.5	92.5	16.34	922.3	0.984	81.0
177	D_15_01288_TS_CS	138	1.467	Frozen Tissue	Ovary cancer	46.5	92.3	17.88	944.7	0.983	86.7
178	D_15_01289_TS_CS	124	1.735	Frozen Tissue	Ovary cancer	45	92.5	14.97	951.6	0.981	74.3
179	D_15_01290_TS_CS	181	1.733	Frozen Tissue	Ovary cancer	45.5	92.7	16.83	1089.2	0.986	85.1
180	D_15_01291_TS_CS	88	1.684	Frozen Tissue	Ovary cancer	45	92.7	10.76	1035.9	0.981	83.7
181	D_15_01292_TS_CS	85	1.420	Frozen Tissue	Ovary cancer	45.5	92.3	14.24	941.2	0.982	89.2
182	D_15_01293_TS_CS	77	1.120	Frozen Tissue	Ovary cancer	46	91.9	11.61	847.7	0.983	70.3
183	D_15_01294_TS_CS	111	1.585	Frozen Tissue	Ovary cancer	45	92.5	11.88	912.3	0.982	100.0
184	D_15_01295_TS_CS	110	1.748	Frozen Tissue	Ovary cancer	45.5	92.6	11.96	936.7	0.983	74.9
185	D_15_01296_TS_CS	85	1.189	Frozen Tissue	Ovary cancer	45.5	92.6	11.47	944	0.984	28.3
186	D_15_01297_TS_CS	142	1.517	Frozen Tissue	Ovary cancer	45.5	92.3	12.47	1006.8	0.982	80.0
187	D_15_01298_TS_CS	136	1.853	Frozen Tissue	Ovary cancer	45.5	92.3	14.41	1015.7	0.985	82.4

188	D_15_01299_TS_CS	116	1.704	Frozen Tissue	Ovary cancer	45.5	91.8	22.35	820.2	0.985	77.8
189	D_15_01300_TS_CS	40	1.296	Frozen Tissue	Ovary cancer	45	92.3	19.58	872.8	0.987	NA
190	D_15_01301_TS_CS	273	1.647	Frozen Tissue	Ovary cancer	46	91.9	27.71	901.9	0.986	78.0
191	D_15_01302_TS_CS	95	1.187	Frozen Tissue	Ovary cancer	45	92.2	24.18	853.9	0.984	93.6
192	D_15_01303_TS_CS	110	1.222	Frozen Tissue	Ovary cancer	45	92.3	21.13	790.1	0.982	90.2
193	D_15_01304_TS_CS	102	1.509	Frozen Tissue	Ovary cancer	46	92.2	21.26	860.5	0.985	79.5
194	D_15_01305_TS_CS	27	1.209	Frozen Tissue	Ovary cancer	45.5	91.8	21.51	1131.5	0.990	38.1
195	D_15_01306_TS_CS	36	1.105	Frozen Tissue	Ovary cancer	45.5	91.8	22.32	885.1	0.987	36.1
196	D_15_01307_TS_CS	136	1.606	Frozen Tissue	Ovary cancer	45	92.2	19.45	1047.4	0.987	57.1
197	D_15_01308_TS_CS	138	1.843	Frozen Tissue	Ovary cancer	45	92.5	24.16	933.9	0.985	82.0
198	D_15_01309_TS_CS	194	1.819	Frozen Tissue	Ovary cancer	46	92.3	17.84	987.4	0.988	100.0
199	D_15_01310_TS_CS	72	0.971	Frozen Tissue	Ovary cancer	46	92	18.99	966	0.987	31.5
200	D_15_01311_TS_CS	124	1.748	Frozen Tissue	Ovary cancer	45	92.6	15.29	1015.8	0.985	82.4
201	D_15_01312_TS_CS	14	0.898	Frozen Tissue	Ovary cancer	45	92.2	14.9	780.3	0.985	NA
202	D_15_01313_TS_CS	15	0.970	Frozen Tissue	Ovary cancer	45.5	91.8	18.63	907	0.987	43.9
203	D_15_01663_TS_CS	107	1.344	Frozen Tissue	Ovary cancer	46	92.3	19.82	908.3	0.986	50.7
204	D_15_02887_TS_CS	71	1.148	Fresh Tissue	Ovary cancer	46	92.7	36.23	794.9	0.982	43.8
205	D_15_02888_TS_CS	70	1.131	Fresh Tissue	Ovary cancer	46	92.2	12.36	895.5	0.984	43.9
206	D_15_02889_TS_CS	126	1.478	Fresh Tissue	Ovary cancer	45	92.8	18.23	875.4	0.981	93.9
207	D_15_04819_TS_CS	83	1.685	Frozen Tissue	Ovary cancer	46	91.4	16.91	904.4	0.984	75.0
208	D_15_04820_TS_CS	119	1.536	Frozen Tissue	Ovary cancer	47	91.3	13.24	972.3	0.986	61.1
209	D_15_04865_TS_CS	78	1.375	Frozen Tissue	Ovary cancer	46	92.7	13	981.5	0.984	92.5
210	D_15_04999_TS_CS	147	1.764	Frozen Tissue	Ovary cancer	NA	NA	11.23	1053.8	0.984	100.0
211	CS11_14_02903	32	1.395	Frozen Tissue	Breast cancer	46	93	22.71	1038.2	0.990	22.7
212	CS11_14_02904	31	1.548	Frozen Tissue	Breast cancer	46	93	24.21	906.7	0.988	NA
213	CS11_14_02905	51	1.359	Frozen Tissue	Breast cancer	46	93.2	20.14	963.8	0.987	27.4
214	CS11_14_02906	67	1.714	Frozen Tissue	Breast cancer	46.5	92.3	26.44	799	0.988	40.9
215	CS11_14_02907	143	1.548	Frozen Tissue	Breast cancer	46	92.7	20.24	886.7	0.984	87.6
216	CS11_14_02908	102	1.244	Frozen Tissue	Breast cancer	46	92.4	15.19	1044.3	0.987	35.6
217	CS11_14_02909	74	1.629	Frozen Tissue	Breast cancer	46	92.6	23.73	855.5	0.987	48.1
218	CS11_14_02910	39	1.538	Frozen Tissue	Breast cancer	46	92.2	35.95	717.9	0.989	38.1
219	CS11_14_02911	55	1.390	Frozen Tissue	Breast cancer	46.5	92.2	31.02	756.4	0.989	27.3
220	CS11_14_02912	51	1.291	Frozen Tissue	Breast cancer	46.5	92.2	27.18	802.8	0.988	25.9
221	CS11_14_02914	147	1.686	Frozen Tissue	Breast cancer	46	92.3	18.09	927.9	0.988	50.7
222	CS11_14_02915	74	1.764	Frozen Tissue	Breast cancer	46	92.7	24.82	958.3	0.989	52.9
223	CS11_14_02916	47	1.641	Frozen Tissue	Breast cancer	46	92.1	18.59	934.7	0.989	22.2
224	CS11_14_02917	26	1.309	Frozen Tissue	Breast cancer	46	92.3	24.3	887.6	0.989	NA
225	CS11_14_02918	132	1.749	Frozen Tissue	Breast cancer	46.5	90.6	35.43	714.8	0.988	61.1
226	CS11_14_02919	124	1.543	Frozen Tissue	Breast cancer	46	93.2	9.63	1069.3	0.983	63.2
227	CS11_14_02920	31	1.627	Frozen Tissue	Breast cancer	46	92.2	28.91	822.6	0.988	32.6
228	CS11_14_02921	150	1.824	Frozen Tissue	Breast cancer	47	91.8	27.24	815.2	0.988	80.8
229	CS11_14_02922	73	1.374	Frozen Tissue	Breast cancer	46	92.2	23.73	865.1	0.988	22.7
230	CS11_14_02923	125	1.256	Frozen Tissue	Breast cancer	48	91.7	25.05	797	0.989	90.7
231	D_15_00791_TS_CS	66	1.533	Fresh Tissue	Breast cancer	46.5	92.5	29.42	758.7	0.988	32.6
232	D_15_00792_TS_CS	155	1.770	Fresh Tissue	Breast cancer	46.5	91.5	19.08	971.9	0.987	100.0
233	D_15_00793_TS_CS	73	1.724	Fresh Tissue	Breast cancer	46	92.2	20.22	1020.6	0.988	83.7
234	D_15_00794_TS_CS	126	1.855	Fresh Tissue	Breast cancer	46	92.7	30.71	821.1	0.987	70.1
235	D_15_00795_TS_CS	24	1.484	Fresh Tissue	Breast cancer	46	92.3	21.68	953	0.989	38.1
236	D_15_00796_TS_CS	147	1.798	Fresh Tissue	Breast cancer	46	92.7	25.05	794.5	0.986	70.1
237	D_15_00809_DP_CS	62	1.402	FFPE DNA	Breast cancer	47	92.5	13.79	1000	0.984	88.0
238	D_15_00903_TS_CS	26	1.207	Frozen Tissue	Breast cancer	45	90.7	14.52	1016.8	0.986	NA
239	D_15_01246_TS_CS	40	1.645	Frozen Tissue	Breast cancer	45.5	92	24.96	866.7	0.988	43.9
240	D_15_01248_TS_CS	68	1.428	Frozen Tissue	Breast cancer	45.5	92.3	22.82	777.3	0.986	41.3
241	D_15_01250_TS_CS	143	1.607	Frozen Tissue	Breast cancer	45.5	92.5	19.96	839	0.986	47.0
242	D_15_01252_TS_CS	64	1.271	Frozen Tissue	Breast cancer	46	91.8	20.15	787.7	0.986	47.3
243	D_15_01254_TS_CS	106	1.584	Frozen Tissue	Breast cancer	45.5	92.3	19.58	876.2	0.985	47.2
244	D_15_01256_TS_CS	123	1.669	Frozen Tissue	Breast cancer	44.5	92.2	17.01	675.9	0.983	68.4
245	D_15_01657_TS_CS	176	1.796	Frozen Tissue	Breast cancer	46	92.3	16.39	1009.1	0.987	77.5
246	D_15_02089_FP_CS	77	1.709	FFPE Slide	Breast cancer	51	84.7	34.68	445.6	0.937	27.3
247	D_15_02114_TS_CS	24	0.710	Frozen Tissue	Breast cancer	45.5	92.9	14.61	1020.8	0.986	63.2
248	D_15_02239_DP_CS	83	1.377	FFPE DNA	Breast cancer	50	89.2	45.48	395.5	0.985	NA
249	D_15_02400_FP_CS	172	1.542	FFPE Slide	Breast cancer	50	84.7	38.89	345.9	0.973	NA
250	D_15_02606_FP_CS	36	1.508	FFPE Slide	Breast cancer	52	85.5	23.06	684.5	0.988	21.8
251	D_15_02617_DP_CS	73	1.007	FFPE DNA	Breast cancer	48	90.7	39.67	530.1	0.987	43.9

252	D_15_02901_FP_CS	99	1.245	FFPE Section(tube)	Breast cancer	51	92.3	32.48	502.9	0.920	94.1
253	D_15_02902_DP_CS	34	1.251	FFPE DNA	Breast cancer	45	88.1	15.34	865.7	0.982	NA
254	D_15_02903_DP_CS	132	1.629	FFPE DNA	Breast cancer	46	88.8	40.46	502.1	0.982	57.1
255	D_15_02904_DP_CS	39	1.591	FFPE DNA	Breast cancer	48	87	26.84	735	0.989	NA
256	D_15_02905_DT_CS	118	1.440	Tissue DNA	Breast cancer	45.5	88.3	14.22	833.1	0.982	43.8
257	D_15_02906_DT_CS	34	1.495	Tissue DNA	Breast cancer	45.5	87.8	24.19	720.9	0.985	NA
258	D_15_02907_DT_CS	30	1.385	Tissue DNA	Breast cancer	45	88.4	20.78	730.1	0.983	NA
259	D_15_02908_DT_CS	41	1.587	Tissue DNA	Breast cancer	45	88.1	20.74	671.3	0.980	NA
260	D_15_02909_DT_CS	47	1.394	Tissue DNA	Breast cancer	45	88.6	19.24	745.5	0.980	25.9
261	D_15_02910_DT_CS	137	1.790	Tissue DNA	Breast cancer	45	87.7	19.46	707.4	0.980	67.1
262	D_15_02911_DT_CS	33	1.450	Tissue DNA	Breast cancer	45	92.2	13.49	955.5	0.984	38.1
263	D_15_02912_DT_CS	28	1.405	Tissue DNA	Breast cancer	45.5	90.8	16.89	775.9	0.984	27.3
264	D_15_02913_DT_CS	46	1.315	Tissue DNA	Breast cancer	45.5	92.4	14.78	911.5	0.986	NA
265	D_15_02914_DT_CS	139	1.668	Tissue DNA	Breast cancer	45.5	91.8	18.38	994.6	0.986	47.3
266	D_15_02915_DT_CS	164	1.769	Tissue DNA	Breast cancer	45.5	91.8	16.36	983	0.984	63.0
267	D_15_02916_DT_CS	55	1.730	Tissue DNA	Breast cancer	45	91.9	20.48	831.8	0.985	78.0
268	D_15_02917_DT_CS	159	1.650	Tissue DNA	Breast cancer	46	91.6	21.59	819.5	0.984	100.0
269	D_15_02918_DT_CS	50	1.514	Tissue DNA	Breast cancer	45	92.2	22.17	807.8	0.983	56.4
270	D_15_02919_DT_CS	54	1.419	Tissue DNA	Breast cancer	45	92.2	16.67	956.9	0.985	28.6
271	D_15_02920_DT_CS	59	1.605	Tissue DNA	Breast cancer	45	92.3	16.53	851	0.984	22.7
272	D_15_02921_DT_CS	19	0.943	Tissue DNA	Breast cancer	45	92.1	13	971.5	0.983	20.2
273	D_15_02922_DT_CS	34	1.104	Tissue DNA	Breast cancer	44.5	92.1	21.82	830.4	0.982	28.9
274	D_15_02923_DT_CS	88	1.421	Tissue DNA	Breast cancer	44.5	92	21.34	865.2	0.981	70.3
275	D_15_02924_DT_CS	71	1.004	Tissue DNA	Breast cancer	44.5	92.4	10.95	941.6	0.977	24.4
276	D_15_02925_DT_CS	65	1.236	Tissue DNA	Breast cancer	46	91.8	41.86	565.4	0.985	30.3
277	D_15_02969_DT_CS	94	1.071	Tissue DNA	Breast cancer	45	91.5	25.42	779.6	0.985	34.3
278	D_15_02970_DT_CS	85	1.192	Tissue DNA	Breast cancer	45	92	25.21	838.8	0.984	94.1
279	D_15_02971_DT_CS	142	1.055	Tissue DNA	Breast cancer	45	91.7	16.9	957	0.982	43.9
280	D_15_02972_DT_CS	91	1.359	Tissue DNA	Breast cancer	45	92	14.45	1107.9	0.986	85.7
281	D_15_04091_DT_CS	167	1.611	Tissue DNA	Breast cancer	46	92.8	25.64	802.4	0.987	55.4
282	D_15_04092_DP_CS	57	1.428	FFPE DNA	Breast cancer	51	91.2	60.45	311.7	0.981	22.2
283	D_15_04106_TS_CS	185	1.887	Fresh Tissue	Breast cancer	47	94.3	28.6	729.3	0.986	79.5
284	D_15_04121_DT_CS	188	1.794	Tissue DNA	Breast cancer	46	93.2	27.81	878.7	0.986	70.1
285	D_15_04122_DT_CS	55	1.421	Tissue DNA	Breast cancer	45	93.2	34.73	735.8	0.982	100.0
286	D_15_04123_DT_CS	105	1.349	Tissue DNA	Breast cancer	45.5	93.2	35.5	809.6	0.985	57.1
287	D_15_04124_DT_CS	167	1.520	Tissue DNA	Breast cancer	46	93	30.9	808.5	0.986	77.8
288	D_15_04125_DT_CS	192	1.685	Tissue DNA	Breast cancer	45	93.1	36.31	688.9	0.982	70.1
289	D_15_04126_DT_CS	57	1.313	Tissue DNA	Breast cancer	45	93.1	28.1	733	0.981	43.9
290	D_15_04127_DT_CS	78	1.509	Tissue DNA	Breast cancer	45	93.3	28.64	755.5	0.981	77.8
291	D_15_04128_DT_CS	152	1.277	Tissue DNA	Breast cancer	45.5	93.3	34.72	585.4	0.980	100.0
292	D_15_04185_DT_CS	12	1.314	Tissue DNA	Breast cancer	46	93.3	29.31	697	0.985	27.3
293	D_15_04186_DT_CS	32	1.112	Tissue DNA	Breast cancer	46	93.2	29.62	605.7	0.982	NA
294	D_15_04187_DT_CS	160	1.644	Tissue DNA	Breast cancer	46	93	24.68	684.4	0.985	56.5
295	D_15_04188_DT_CS	74	0.961	Tissue DNA	Breast cancer	45	93	27.78	648.1	0.980	38.1
296	D_15_04189_DT_CS	129	1.736	Tissue DNA	Breast cancer	45	93.2	27.28	685.6	0.983	68.4
297	D_15_04219_DT_CS	133	1.655	Tissue DNA	Breast cancer	46	92.9	11.58	1067.1	0.983	53.5
298	D_15_04262_DT_CS	77	1.499	Tissue DNA	Breast cancer	46	92.7	14.63	979.4	0.983	49.2
299	D_15_04263_DT_CS	115	1.607	Tissue DNA	Breast cancer	45.5	92.8	24.16	875.8	0.985	59.2
300	D_15_04265_DT_CS	18	1.301	Tissue DNA	Breast cancer	45.5	93.2	18.73	935.1	0.985	22.2
301	D_15_04266_DT_CS	103	1.472	Tissue DNA	Breast cancer	45.5	93	22.87	955.3	0.985	100.0
302	D_15_04267_DT_CS	99	1.654	Tissue DNA	Breast cancer	45.5	93.2	18.76	863.2	0.982	60.6
303	D_15_04268_DT_CS	122	1.839	Tissue DNA	Breast cancer	46	93.5	16.7	937.5	0.984	79.5
304	D_15_04269_DT_CS	93	1.666	Tissue DNA	Breast cancer	46	93.6	15.84	999	0.986	77.8
305	D_15_04270_DT_CS	34	0.856	Tissue DNA	Breast cancer	45	93.7	21.05	912.8	0.984	21.0
306	D_15_04271_DT_CS	86	1.142	Tissue DNA	Breast cancer	45	93.2	21.95	936.4	0.986	94.1
307	D_15_04272_DT_CS	55	1.012	Tissue DNA	Breast cancer	46	93.4	25.11	826.8	0.986	28.9
308	D_15_04274_DT_CS	60	1.633	Tissue DNA	Breast cancer	46	93.2	23.74	860.9	0.985	48.5
309	D_15_04275_DT_CS	31	1.708	Tissue DNA	Breast cancer	46	93.6	21.43	844.3	0.985	NA
310	D_15_04276_DT_CS	84	1.096	Tissue DNA	Breast cancer	NA	NA	17.27	1047.4	0.986	100.0
311	D_15_04277_DT_CS	16	1.143	Tissue DNA	Breast cancer	NA	NA	11.33	1301.1	0.985	22.2
312	D_15_04278_DT_CS	84	1.620	Tissue DNA	Breast cancer	NA	NA	20.14	985.6	0.983	59.2
313	D_15_04279_DT_CS	57	1.403	Tissue DNA	Breast cancer	46	95	10.56	900.2	0.981	27.3
314	D_15_04282_DT_CS	84	1.767	Tissue DNA	Breast cancer	46	94.6	13.08	856	0.983	61.1

315	D_15_04283_DT_CS	11	1.295	Tissue DNA	Breast cancer	46	94.8	13.69	913.6	0.983	50.0
316	D_15_04284_DT_CS	49	1.275	Tissue DNA	Breast cancer	45.5	93.6	16.83	1023.7	0.984	20.7
317	D_15_04285_DT_CS	48	1.284	Tissue DNA	Breast cancer	45.5	92.8	16.78	685.8	0.980	32.6
318	D_15_04286_DT_CS	109	1.499	Tissue DNA	Breast cancer	NA	NA	20.71	1148.9	0.987	85.7
319	D_15_04287_DT_CS	170	1.785	Tissue DNA	Breast cancer	NA	NA	17.87	1089.3	0.982	66.6
320	D_15_04288_DT_CS	113	1.750	Tissue DNA	Breast cancer	NA	NA	19.2	1182.9	0.985	78.0
321	D_15_04289_DT_CS	103	1.462	Tissue DNA	Breast cancer	NA	NA	20.72	1134.3	0.985	70.3
322	D_15_04290_DT_CS	48	1.077	Tissue DNA	Breast cancer	NA	NA	19.24	1139.3	0.985	27.6
323	D_15_04291_DT_CS	50	1.487	Tissue DNA	Breast cancer	NA	NA	13.78	1130.6	0.983	66.7
324	D_15_04292_DT_CS	45	1.296	Tissue DNA	Breast cancer	NA	NA	16.1	1068.9	0.985	21.4
325	D_15_04294_DT_CS	66	1.692	Tissue DNA	Breast cancer	46	94.7	15.07	847.1	0.980	68.4
326	D_15_04295_DT_CS	76	1.440	Tissue DNA	Breast cancer	46	93.8	13	1000.5	0.984	31.5
327	D_15_04296_DT_CS	106	0.903	Tissue DNA	Breast cancer	46	93.6	11.65	1116.8	0.985	85.7
328	D_15_04297_DT_CS	41	1.411	Tissue DNA	Breast cancer	46	92.8	16.99	979	0.985	43.9
329	D_15_04298_DT_CS	143	1.733	Tissue DNA	Breast cancer	46	93.3	17.94	982.8	0.986	79.2
330	D_15_04300_DT_CS	63	1.721	Tissue DNA	Breast cancer	NA	NA	15.34	1192.3	0.987	43.8
331	D_15_04301_DT_CS	33	0.874	Tissue DNA	Breast cancer	NA	NA	15.6	1074.6	0.983	33.3
332	D_15_04302_DT_CS	95	1.072	Tissue DNA	Breast cancer	NA	NA	20.25	1073	0.984	100.0
333	D_15_04306_DT_CS	125	1.840	Tissue DNA	Breast cancer	NA	NA	21.27	1027	0.987	78.0
334	D_15_04307_DT_CS	137	1.399	Tissue DNA	Breast cancer	NA	NA	18.87	1042.8	0.986	70.3
335	D_15_04308_DT_CS	107	1.664	Tissue DNA	Breast cancer	NA	NA	18.01	1097.2	0.981	54.0
336	D_15_04310_DT_CS	35	1.412	Tissue DNA	Breast cancer	NA	NA	15.06	1019.1	0.984	70.3
337	D_15_04314_DT_CS	30	0.865	Tissue DNA	Breast cancer	NA	NA	16.42	1259.9	0.987	27.3
338	D_15_04320_DT_CS	134	1.121	Tissue DNA	Breast cancer	NA	NA	11.28	1137.8	0.984	63.2
339	D_15_04322_DT_CS	80	1.399	Tissue DNA	Breast cancer	NA	NA	14.81	1083.5	NA	50.0
340	D_15_04323_DT_CS	34	1.280	Tissue DNA	Breast cancer	NA	NA	12.25	1156.2	0.986	NA
341	D_15_04328_DT_CS	164	1.738	Tissue DNA	Breast cancer	NA	NA	13.66	820.2	0.983	71.8
342	D_15_04329_DT_CS	32	1.645	Tissue DNA	Breast cancer	NA	NA	19.07	1010.5	0.983	32.6
343	D_15_04339_DT_CS	125	1.451	Tissue DNA	Breast cancer	NA	NA	20.77	1133.4	0.987	94.1
344	D_15_04341_DT_CS	50	1.247	Tissue DNA	Breast cancer	NA	NA	16.5	1053.3	0.983	NA
345	D_15_04346_DT_CS	98	1.041	Tissue DNA	Breast cancer	NA	NA	18.56	1120.8	0.985	56.4
346	D_15_04348_DT_CS	47	1.442	Tissue DNA	Breast cancer	NA	NA	11.65	1139.7	0.983	27.4
347	D_15_04350_DT_CS	103	1.744	Tissue DNA	Breast cancer	NA	NA	15.04	1148.5	0.985	100.0
348	D_15_04354_DT_CS	74	1.178	Tissue DNA	Breast cancer	NA	NA	24.23	976.8	NA	32.9
349	D_15_04359_DT_CS	189	1.661	Tissue DNA	Breast cancer	NA	NA	16.09	1292.5	0.986	100.0
350	D_15_04363_DT_CS	68	1.437	Tissue DNA	Breast cancer	NA	NA	19.97	1093.7	0.983	57.1
351	D_15_04364_DT_CS	53	1.219	Tissue DNA	Breast cancer	NA	NA	20.69	1022.7	0.985	NA
352	D_15_04365_DT_CS	76	1.461	Tissue DNA	Breast cancer	NA	NA	17.61	1122.5	0.984	100.0
353	D_15_04368_DT_CS	81	1.530	Tissue DNA	Breast cancer	NA	NA	21.48	1004.2	0.983	100.0
354	D_15_04370_DT_CS	170	1.548	Tissue DNA	Breast cancer	NA	NA	17.26	1068.4	0.985	71.8
355	D_15_04371_DT_CS	80	1.588	Tissue DNA	Breast cancer	NA	NA	18.17	1051.7	0.985	100.0
356	D_15_04376_DT_CS	20	0.687	Tissue DNA	Breast cancer	NA	NA	13.72	1269.6	0.985	NA
357	D_15_04380_DT_CS	74	1.068	Tissue DNA	Breast cancer	NA	NA	18.95	1006	0.982	63.2
358	D_15_04384_DT_CS	64	1.007	Tissue DNA	Breast cancer	NA	NA	15.47	1192.2	0.984	63.2
359	D_15_04385_DT_CS	39	1.170	Tissue DNA	Breast cancer	NA	NA	20.32	1190.3	0.987	27.6
360	D_15_04388_DT_CS	98	1.310	Tissue DNA	Breast cancer	NA	NA	22.93	1127.2	0.979	56.4
361	D_15_04390_DT_CS	28	0.808	Tissue DNA	Breast cancer	NA	NA	12.49	1210.5	0.982	NA
362	D_15_04454_DT_CS	113	1.697	Tissue DNA	Breast cancer	46	93	21.6	886.2	0.987	77.9
363	D_15_04455_DT_CS	41	1.482	Tissue DNA	Breast cancer	46	93.1	17.79	818.9	0.987	53.6
364	D_15_04456_DT_CS	164	1.577	Tissue DNA	Breast cancer	46	93	23.05	825.1	0.987	73.4
365	D_15_04457_DT_CS	74	1.100	Tissue DNA	Breast cancer	46	92.7	20.64	934.9	0.988	37.1
366	D_15_04458_DT_CS	157	1.168	Tissue DNA	Breast cancer	45	93	19.5	855.1	0.986	100.0
367	D_15_04459_DT_CS	33	1.427	Tissue DNA	Breast cancer	45	92.1	21.14	955.4	0.988	32.6
368	D_15_04460_DT_CS	111	0.633	Tissue DNA	Breast cancer	45	92.9	26.34	831.4	0.987	38.1
369	D_15_04461_DT_CS	164	1.436	Tissue DNA	Breast cancer	NA	NA	18.32	1166	0.986	63.2
370	D_15_04506_DT_CS	180	1.710	Tissue DNA	Breast cancer	45	91.8	19.3	1038.2	0.986	100.0
371	D_15_04507_DT_CS	127	1.593	Tissue DNA	Breast cancer	46	92.4	13.85	1104.9	0.986	57.5
372	D_15_04508_DT_CS	44	1.583	Tissue DNA	Breast cancer	46	92.1	17.96	1041.9	0.985	32.6
373	D_15_04509_DP_CS	36	1.111	FFPE DNA	Breast cancer	49.5	91.5	40.49	609.4	0.990	24.9
374	D_15_04510_DT_CS	16	1.630	Tissue DNA	Breast cancer	46	92.9	12.72	1168.8	0.985	NA
375	D_15_04511_DT_CS	164	1.622	Tissue DNA	Breast cancer	46	92.4	17.14	986.1	0.987	94.1
376	D_15_04801_DT_CS	88	1.476	Tissue DNA	Breast cancer	45	91.5	29.27	768.5	0.982	55.1
377	D_15_04802_DT_CS	124	1.849	Tissue DNA	Breast cancer	45.5	90.3	19.18	996.7	0.985	63.0
378	D_15_04803_DT_CS	31	0.756	Tissue DNA	Breast cancer	45.5	90.4	18.96	1037.5	0.988	NA

379	D_15_04804_DT_CS	131	1.564	Tissue DNA	Breast cancer	46	91.2	12.97	1053.4	0.986	48.1
380	D_15_04805_DP_CS	149	1.720	FFPE DNA	Breast cancer	48	90.2	41.59	705.1	0.990	48.4
381	D_15_04806_DP_CS	30	1.090	FFPE DNA	Breast cancer	53	89.3	18.77	960.2	0.991	22.2
382	D_15_04807_TS_CS	44	1.097	Frozen Tissue	Breast cancer	46	91	18.07	952.1	0.987	70.3
383	D_15_04855_DT_CS	83	1.572	Tissue DNA	Breast cancer	45	91.7	13.09	1131.7	0.985	70.3
384	D_15_04856_DT_CS	9	1.215	Tissue DNA	Breast cancer	45	91.8	14.74	1178.8	0.987	NA
385	D_15_04857_DT_CS	32	1.086	Tissue DNA	Breast cancer	45	91.8	14.2	1183.9	0.987	24.6
386	D_15_04858_DT_CS	72	1.531	Tissue DNA	Breast cancer	46	91.3	14.82	1160.9	0.986	48.1
387	D_15_04859_DT_CS	120	1.502	Tissue DNA	Breast cancer	45.5	91.8	14.94	1266.4	0.989	40.9
388	D_15_04860_DT_CS	65	1.225	Tissue DNA	Breast cancer	45	91.6	13.92	1162.8	0.986	27.3
389	D_15_04861_DT_CS	43	1.300	Tissue DNA	Breast cancer	45	91.4	13.3	1229.8	0.985	38.7
390	D_15_04862_DT_CS	16	1.474	Tissue DNA	Breast cancer	45	91.3	10.66	1031.3	0.984	NA
391	D_15_04863_DT_CS	122	1.464	Tissue DNA	Breast cancer	45.5	91.7	11.64	1072.4	0.986	50.7
392	D_15_04864_DT_CS	31	1.028	Tissue DNA	Breast cancer	45	91.7	13.08	1076.1	0.985	33.3
393	D_15_04927_DP_CS	70	1.502	FFPE DNA	Breast cancer	47.5	92.6	18.01	1056.7	0.989	43.9
394	D_15_04928_DP_CS	189	1.596	FFPE DNA	Breast cancer	49	91.6	23.9	907.4	0.991	55.2
395	D_15_04929_DP_CS	166	1.577	FFPE DNA	Breast cancer	54	90.2	14.64	946.3	0.990	100.0
396	D_15_04939_TS_CS	95	1.711	Frozen Tissue	Breast cancer	45.5	93.3	18.39	1147.2	0.988	77.8
397	D_15_04980_DT_CS	16	1.034	Tissue DNA	Breast cancer	NA	NA	20.4	940.8	0.988	43.9
398	D_15_04981_DT_CS	16	1.474	Tissue DNA	Breast cancer	NA	NA	15.58	841.1	0.985	22.2
399	D_15_04987_DT_CS	208	1.915	Tissue DNA	Breast cancer	NA	NA	16.54	920.9	0.988	72.9
400	D_15_04988_DT_CS	122	1.632	Tissue DNA	Breast cancer	NA	NA	26.49	874.8	0.986	82.2
401	D_15_04989_DT_CS	150	1.726	Tissue DNA	Breast cancer	NA	NA	23.97	1037.4	0.987	66.7
402	D_15_04990_DT_CS	190	1.336	Tissue DNA	Breast cancer	NA	NA	24.12	930	0.988	68.4
403	D_15_04991_DT_CS	65	1.405	Tissue DNA	Breast cancer	NA	NA	22.48	995.1	0.989	66.7
404	D_15_04992_DT_CS	120	1.733	Tissue DNA	Breast cancer	NA	NA	17.82	876.4	0.982	100.0
405	D_15_04993_DT_CS	188	1.728	Tissue DNA	Breast cancer	NA	NA	14.2	739.1	0.983	73.4
406	D_15_04994_DT_CS	26	1.116	Tissue DNA	Breast cancer	NA	NA	9.93	1098.7	0.988	94.1
407	D_15_04995_DT_CS	99	1.621	Tissue DNA	Breast cancer	NA	NA	22.71	1075.6	0.990	48.4
408	D_15_04996_DT_CS	85	0.979	Tissue DNA	Breast cancer	NA	NA	10.38	1146.4	0.985	56.4
409	D_15_05037_DT_CS	104	1.519	Tissue DNA	Breast cancer	NA	NA	12.1	1131.8	0.985	56.4
410	D_15_05038_DT_CS	92	1.842	Tissue DNA	Breast cancer	NA	NA	13.49	957.1	0.983	73.4
411	D_15_05039_DT_CS	161	1.802	Tissue DNA	Breast cancer	NA	NA	9.52	1061.9	0.985	73.4
412	D_15_05040_DT_CS	197	1.833	Tissue DNA	Breast cancer	NA	NA	10.17	1047.2	0.983	77.8
413	D_15_05041_DT_CS	143	1.727	Tissue DNA	Breast cancer	NA	NA	13.34	1116.1	0.986	85.4
414	D_15_05042_DT_CS	61	1.482	Tissue DNA	Breast cancer	NA	NA	9.61	1062.6	0.983	52.4
415	D_15_05043_DT_CS	63	1.020	Tissue DNA	Breast cancer	NA	NA	11.27	1092.4	0.985	25.9
416	D_15_05044_DT_CS	11	1.264	Tissue DNA	Breast cancer	NA	NA	13.87	1244.4	0.986	22.2
417	D_15_05045_DT_CS	83	1.652	Tissue DNA	Breast cancer	NA	NA	14.76	1068.2	0.986	52.5
418	D_15_05046_DT_CS	96	1.710	Tissue DNA	Breast cancer	NA	NA	11.59	1028.3	0.983	70.1
419	D_15_05047_DT_CS	28	1.177	Tissue DNA	Breast cancer	NA	NA	13.88	1103.1	0.984	27.3
420	D_15_05048_DT_CS	8	1.255	Tissue DNA	Breast cancer	NA	NA	12.13	1081.1	0.983	21.4
421	D_15_05049_DT_CS	58	1.245	Tissue DNA	Breast cancer	NA	NA	11.61	1123.5	0.985	38.1
422	D_15_05061_DT_CS	136	1.458	Tissue DNA	Breast cancer	NA	NA	29.12	1039.9	0.987	70.3
423	D_15_05062_DT_CS	19	1.357	Tissue DNA	Breast cancer	NA	NA	12.71	976.8	0.986	32.6
424	D_15_05063_DT_CS	80	1.682	Tissue DNA	Breast cancer	NA	NA	13.67	1163.3	0.987	56.4
425	D_15_05064_DT_CS	125	1.658	Tissue DNA	Breast cancer	NA	NA	11.47	942.8	0.985	100.0
426	D_15_05065_DT_CS	112	1.580	Tissue DNA	Breast cancer	NA	NA	12.48	1118	0.985	100.0
427	D_15_05066_DT_CS	131	1.600	Tissue DNA	Breast cancer	NA	NA	15.06	1438.3	0.987	49.5
428	D_15_05067_DT_CS	190	1.766	Tissue DNA	Breast cancer	NA	NA	12.97	1069.3	0.985	74.9
429	D_15_05068_DT_CS	111	1.433	Tissue DNA	Breast cancer	NA	NA	10.48	1070.2	0.983	50.7
430	D_15_05069_DT_CS	98	1.640	Tissue DNA	Breast cancer	NA	NA	11.11	1153	0.985	56.4
431	D_15_05070_DT_CS	109	1.641	Tissue DNA	Breast cancer	NA	NA	9.83	1164.7	0.984	71.8
432	D_15_05201_DT_CS	78	1.320	Tissue DNA	Breast cancer	NA	NA	12.07	1268.8	NA	46.9
433	D_15_05202_DT_CS	69	1.585	Tissue DNA	Breast cancer	NA	NA	10.8	1399.2	NA	63.2
434	D_15_05203_DT_CS	163	1.737	Tissue DNA	Breast cancer	NA	NA	11.84	1154.4	NA	100.0
435	D_15_05204_DT_CS	150	1.710	Tissue DNA	Breast cancer	NA	NA	11.24	1203.3	NA	57.4
436	D_15_05205_DT_CS	34	0.705	Tissue DNA	Breast cancer	NA	NA	13.41	1377.4	NA	32.6
437	D_15_05206_DT_CS	21	1.087	Tissue DNA	Breast cancer	NA	NA	15.47	1004	NA	38.1
438	D_15_05207_DT_CS	88	1.800	Tissue DNA	Breast cancer	NA	NA	13.22	1185.8	NA	75.0
439	D_15_05209_DT_CS	64	1.367	Tissue DNA	Breast cancer	NA	NA	15.15	1153.8	NA	100.0
440	D_15_05210_DT_CS	186	1.748	Tissue DNA	Breast cancer	NA	NA	15.72	983.8	NA	73.4
441	D_15_05211_DT_CS	58	1.441	Tissue DNA	Breast cancer	NA	NA	12.71	1008	NA	31.5
442	D_15_05212_DT_CS	171	1.706	Tissue DNA	Breast cancer	NA	NA	11.44	1036.6	NA	68.4

443	D_15_05213_DT_CS	67	1.490	Tissue DNA	Breast cancer	NA	NA	11.01	1098.2	NA	94.1
444	D_15_05214_DT_CS	99	1.580	Tissue DNA	Breast cancer	NA	NA	14.18	1033.3	NA	27.3
445	15_00232_TS_CS	16	1.371	Frozen Tissue	Sarcoma	46	92.1	12.92	1111.6	0.990	27.3
446	15_00430_DP_CS	39	1.676	FFPE DNA	Sarcoma	50	92.2	38.81	688.7	0.991	100.0
447	D_15_00808_DP_CS	20	1.121	FFPE DNA	Sarcoma	48	92.5	26.5	894	0.991	24.6
448	D_15_00811_DP_CS	117	1.400	FFPE DNA	Sarcoma	51	90.8	46.94	466	0.987	77.8
449	D_15_00859_DP_CS	57	1.710	FFPE DNA	Sarcoma	48	91.8	18.77	939.1	0.989	22.2
450	D_15_00862_DP_CS	53	1.672	FFPE DNA	Sarcoma	49	90.8	35.19	691.3	0.990	70.3
451	D_15_00929_TS_CS	163	1.746	Frozen Tissue	Sarcoma	45.5	92.5	16.24	936.4	0.983	85.7
452	D_15_00930_TS_CS	19	1.247	Frozen Tissue	Sarcoma	45	92.5	17.53	880.8	0.985	32.6
453	D_15_00931_TS_CS	5	1.332	Frozen Tissue	Sarcoma	45.5	93	12.51	955.2	0.984	NA
454	D_15_00932_TS_CS	145	1.552	Frozen Tissue	Sarcoma	46	92.4	15.71	1093.6	0.988	100.0
455	D_15_01220_DP_CS	120	1.667	FFPE DNA	Sarcoma	49.5	91.7	23.91	902	0.989	64.2
456	D_15_01226_DP_CS	118	1.818	FFPE DNA	Sarcoma	50	91.2	34.59	897.6	0.992	71.8
457	D_15_01239_TS_CS	16	1.041	Frozen Tissue	Sarcoma	46	92.9	33.15	680	0.985	88.9
458	D_15_01241_TS_CS	10	1.168	Frozen Tissue	Sarcoma	46	93.1	29.47	795.1	0.987	NA
459	D_15_01244_TS_CS	25	1.155	Frozen Tissue	Sarcoma	46	92.7	27.28	815.6	0.986	38.1
460	D_15_01426_DP_CS	92	1.662	FFPE DNA	Sarcoma	47	91.5	33.4	744.6	0.989	55.1
461	D_15_01664_TS_CS	33	1.464	Frozen Tissue	Sarcoma	45.5	92	19.38	788.6	0.984	93.6
462	D_15_01665_TS_CS	106	1.682	Frozen Tissue	Sarcoma	46	92.8	19	968.7	0.985	73.4
463	D_15_01926_DP_CS	144	1.783	FFPE DNA	Sarcoma	48	92.2	46.13	546.8	0.990	64.6
464	D_15_02011_DP_CS	224	1.321	FFPE DNA	Sarcoma	47	89.6	13.19	995.4	0.989	61.1
465	D_15_02012_DP_CS	37	1.619	FFPE DNA	Sarcoma	47.5	90.4	10.18	993.1	0.988	38.1
466	D_15_02240_DP_CS	37	1.585	FFPE DNA	Sarcoma	52.5	89.2	34.98	832.6	0.986	22.2
467	D_15_02591_DP_CS	117	1.253	FFPE DNA	Sarcoma	49	90.9	47.63	493.6	0.986	84.4
468	D_15_02595_DP_CS	28	1.166	FFPE DNA	Sarcoma	50	89.9	31.39	710.2	0.990	24.4
469	D_15_02614_DP_CS	35	1.508	FFPE DNA	Sarcoma	52	89.9	22.3	995.1	0.987	32.6
470	D_15_02618_DP_CS	30	1.580	FFPE DNA	Sarcoma	54	90.1	39.7	705.5	0.975	NA
471	D_15_02890_TS_CS	11	1.162	Fresh Tissue	Sarcoma	46.5	92.7	18.89	886.5	0.987	NA
472	D_15_02891_TS_CS	9	1.427	Fresh Tissue	Sarcoma	45.5	92.9	16.5	865.9	0.984	27.3
473	D_15_02973_DT_CS	80	1.458	Tissue DNA	Sarcoma	47	90	31.95	655	0.986	63.0
474	D_15_02974_DT_CS	25	0.959	Tissue DNA	Sarcoma	46.5	90.6	35.87	616.8	0.987	NA
475	D_15_02975_DT_CS	16	1.244	Tissue DNA	Sarcoma	46	90.8	32.56	712.8	0.986	NA
476	D_15_02976_DT_CS	13	1.157	Tissue DNA	Sarcoma	45.5	91.3	28.71	706.4	0.987	22.2
477	D_15_02977_DT_CS	19	1.589	Tissue DNA	Sarcoma	46.5	91.5	28.38	746.4	0.987	27.3
478	D_15_02978_DT_CS	20	1.597	Tissue DNA	Sarcoma	47	90.7	28.76	667.4	0.987	NA
479	D_15_04036_DP_CS	13	1.119	FFPE DNA	Sarcoma	50	93.9	48.25	450.8	0.987	27.3
480	D_15_04137_DP_CS	13	0.790	FFPE DNA	Sarcoma	49	94.2	49.1	366.3	0.984	22.2
481	D_15_04139_DP_CS	65	1.377	FFPE DNA	Sarcoma	50	92.5	63.74	310.3	0.973	94.1
482	D_15_04148_DT_CS	13	0.687	Tissue DNA	Sarcoma	46	92.7	34.08	618.9	0.985	NA
483	D_15_04261_DP_CS	74	1.519	FFPE DNA	Sarcoma	48	92.3	15.07	1067.7	0.990	100.0
484	D_15_04481_DP_CS	12	1.589	FFPE DNA	Sarcoma	49	92.7	34.82	811.4	0.991	NA
485	D_15_04503_DP_CS	12	1.075	FFPE DNA	Sarcoma	47	91.9	20.43	1075.5	0.990	NA
486	D_15_04816_TS_CS	18	1.087	Frozen Tissue	Sarcoma	46	90.4	18.1	831.7	0.986	56.4
487	D_15_04817_TS_CS	163	1.711	Frozen Tissue	Sarcoma	46	91.3	15.54	1005.6	0.985	100.0
488	D_15_04850_DP_CS	11	1.121	FFPE DNA	Sarcoma	50	90.3	34.77	835	0.992	22.2
489	D_15_04885_DP_CS	69	1.799	FFPE DNA	Sarcoma	51	90.8	39.66	583.4	0.989	86.4
490	D_15_04941_FP_CS	188	1.681	FFPE Section(tube)	Sarcoma	57	89.2	20.05	844.9	0.923	84.9
491	D_15_05075_TS_CS	108	1.694	Fresh Tissue	Sarcoma	NA	NA	10.9	1076.3	0.981	93.6
492	15_00045_TS_CS	133	1.857	Frozen Tissue	Lung cancer	45	92.3	14.06	934.1	0.986	81.0
493	15_00046_TS_CS	84	1.505	Frozen Tissue	Lung cancer	46	92.3	11.6	1002.3	0.988	50.7
494	15_00047_CP_CS	9	1.273	Cultured cell pellet	Lung cancer	46	92	12.13	753.5	0.990	32.6
495	15_00048_CP_CS	13	1.479	Cultured cell pellet	Lung cancer	46	91.7	16.65	816.7	0.993	38.1
496	15_00260_DT_CS	87	1.344	Tissue DNA	Lung cancer	45	92.2	12.94	1200.7	0.988	NA
497	15_00261_DT_CS	9	1.149	Tissue DNA	Lung cancer	45	92.2	11.28	1026	0.985	NA
498	15_00413_DP_CS	50	1.530	FFPE DNA	Lung cancer	49.5	92.4	39.37	625	0.990	100.0
499	15_00414_DP_CS	50	1.179	FFPE DNA	Lung cancer	47	93.2	16.02	1218	0.990	NA
500	15_00422_DT_CS	53	1.252	Tissue DNA	Lung cancer	45.5	93.2	16.01	897.7	0.987	NA
501	15_00436_DT_CS	27	0.874	Tissue DNA	Lung cancer	45.5	93.6	13.06	1040.5	0.989	27.3
502	15_00438_DT_CS	180	1.772	Tissue DNA	Lung cancer	45	93.2	13.12	1048.4	0.987	73.4
503	D_15_00801_DP_CS	106	1.259	FFPE DNA	Lung cancer	49	92.4	46.4	673.2	0.991	50.0
504	D_15_00802_DP_CS	10	1.089	FFPE DNA	Lung cancer	47	92.1	14.41	1098.7	0.990	NA

505	D_15_00815_DP_CS	46	1.100	FFPE DNA	Lung cancer	53	90.3	27.03	758.6	0.992	27.4
506	D_15_00816_DP_CS	32	1.192	FFPE DNA	Lung cancer	47	92.6	12.65	921.6	0.988	22.2
507	D_15_00870_DT_CS	76	1.419	Tissue DNA	Lung cancer	45	93.8	13.87	877.1	0.985	NA
508	D_15_00871_DT_CS	188	1.858	Tissue DNA	Lung cancer	45	93.6	14.87	868.2	0.983	27.3
509	D_15_01021_DT_CS	33	1.571	Tissue DNA	Lung cancer	45.5	93.1	17.81	915.5	0.988	27.3
510	D_15_01089_DT_CS	38	1.460	Tissue DNA	Lung cancer	45.5	93.5	18.54	1083.6	0.988	22.2
511	D_15_01090_DT_CS	70	1.626	Tissue DNA	Lung cancer	45	93.2	20.42	1002.7	0.987	NA
512	D_15_01091_DT_CS	88	1.715	Tissue DNA	Lung cancer	45	93.4	22.96	1062.3	0.987	64.9
513	D_15_01107_DT_CS	40	1.327	Tissue DNA	Lung cancer	45	92.4	15.4	1029.6	0.986	31.7
514	D_15_01108_DT_CS	153	1.821	Tissue DNA	Lung cancer	46	92.4	16.81	1007.7	0.986	73.7
515	D_15_01153_DT_CS	106	1.395	Tissue DNA	Lung cancer	46	92.3	13.14	1307.5	0.987	46.2
516	D_15_01154_DT_CS	103	1.496	Tissue DNA	Lung cancer	46	93.2	18.29	936.8	0.988	35.6
517	D_15_01156_DT_CS	92	1.417	Tissue DNA	Lung cancer	46	93.5	12.53	995.9	0.987	32.6
518	D_15_01157_DT_CS	105	1.306	Tissue DNA	Lung cancer	46	93.2	16.98	936.3	0.987	63.2
519	D_15_01184_DE_CS	6	1.242	DNA ??	Lung cancer	NA	NA	24.84	792.1	0.987	22.2
520	D_15_01188_DE_CS	10	1.366	DNA ??	Lung cancer	NA	NA	14.24	1016.2	0.986	NA
521	D_15_01189_DE_CS	14	1.433	DNA ??	Lung cancer	NA	NA	15.19	1094.1	0.986	38.1
522	D_15_01193_TS_CS	11	1.169	Frozen Tissue	Lung cancer	NA	NA	13.85	1022.9	0.986	NA
523	D_15_01194_TS_CS	4	1.040	Frozen Tissue	Lung cancer	NA	NA	19.16	1044.5	0.987	NA
524	D_15_01231_DT_CS	154	1.605	Tissue DNA	Lung cancer	45	92.9	15.76	917.6	0.987	100.0
525	D_15_01262_DT_CS	26	1.284	Tissue DNA	Lung cancer	45	92.8	16.59	942.8	0.988	27.3
526	D_15_01428_DP_CS	115	1.351	FFPE DNA	Lung cancer	48	91.1	19.84	883.9	0.990	77.8
527	D_15_01438_DT_CS	76	1.741	Tissue DNA	Lung cancer	46	93.6	13.81	841.2	0.984	43.9
528	D_15_01452_TS_CS	54	1.142	Frozen Tissue	Lung cancer	NA	NA	15.41	1149.2	0.987	93.6
529	D_15_01457_DT_CS	28	1.496	Tissue DNA	Lung cancer	45.5	94.3	19.24	744.5	0.985	NA
530	D_15_01461_DT_CS	9	0.937	Tissue DNA	Lung cancer	46	92.6	20.89	923.6	0.988	32.6
531	D_15_01561_DT_CS	102	1.438	Tissue DNA	Lung cancer	46	93.7	16.5	845	0.987	63.0
532	D_15_01650_TS_CS	137	1.437	Frozen Tissue	Lung cancer	46	92.2	10.55	1055.5	0.986	46.9
533	D_15_01651_TS_CS	30	1.376	Frozen Tissue	Lung cancer	46	92	10.22	884.1	0.984	24.6
534	D_15_01653_TS_CS	74	1.802	Frozen Tissue	Lung cancer	45.5	92.6	9.91	1105.8	0.985	68.4
535	D_15_01660_TS_CS	176	1.647	Frozen Tissue	Lung cancer	45	93.8	17.33	877.5	0.984	81.0
536	D_15_01674_DT_CS	41	1.364	Tissue DNA	Lung cancer	46	93.2	12.2	986.8	0.985	20.2
537	D_15_01675_DT_CS	54	1.459	Tissue DNA	Lung cancer	45.5	93.5	13.72	907	0.985	46.2
538	D_15_01676_DT_CS	291	1.724	Tissue DNA	Lung cancer	45	93.4	16.72	1092.7	0.981	48.5
539	D_15_01678_DT_CS	127	1.496	Tissue DNA	Lung cancer	46	93.3	11.39	1008.4	0.986	62.1
540	D_15_01680_DC_CS	13	1.439	Cell DNA	Lung cancer	NA	NA	21.68	1035.9	0.986	NA
541	D_15_01681_DC_CS	15	0.803	Cell DNA	Lung cancer	NA	NA	26.38	1104.8	0.987	NA
542	D_15_01682_DC_CS	4	1.386	Cell DNA	Lung cancer	46	93.3	26.67	834	0.988	NA
543	D_15_01683_DC_CS	13	1.072	Cell DNA	Lung cancer	NA	NA	21.57	1081.9	0.987	38.1
544	D_15_01684_DC_CS	63	1.235	Cell DNA	Lung cancer	NA	NA	29.18	1014.7	0.987	27.4
545	D_15_01685_DC_CS	12	0.960	Cell DNA	Lung cancer	NA	NA	22.26	1003.2	0.987	27.3
546	D_15_01686_DC_CS	101	1.582	Cell DNA	Lung cancer	NA	NA	21.06	1059.2	0.983	46.2
547	D_15_01687_DC_CS	13	0.937	Cell DNA	Lung cancer	NA	NA	21.29	1102.8	0.988	NA
548	D_15_01688_DC_CS	18	1.059	Cell DNA	Lung cancer	NA	NA	20.27	1060.2	0.988	21.4
549	D_15_01689_DC_CS	16	0.822	Cell DNA	Lung cancer	NA	NA	43.12	759.6	0.965	20.2
550	D_15_01690_DC_CS	25	1.111	Cell DNA	Lung cancer	46	93.1	18.38	915.3	0.987	27.3
551	D_15_01691_DC_CS	10	1.168	Cell DNA	Lung cancer	NA	NA	18.55	1042.9	0.988	32.6
552	D_15_01692_DC_CS	12	1.234	Cell DNA	Lung cancer	NA	NA	17.61	918.7	0.987	32.6
553	D_15_01693_DC_CS	13	1.179	Cell DNA	Lung cancer	NA	NA	19.36	985.2	0.988	27.3
554	D_15_01767_DT_CS	68	1.525	Tissue DNA	Lung cancer	46	92.8	26.39	954.2	0.987	48.5
555	D_15_01799_DT_CS	36	1.336	Tissue DNA	Lung cancer	45	92.5	11.5	975.7	0.984	32.6
556	D_15_01800_DT_CS	139	1.531	Tissue DNA	Lung cancer	45.5	92.5	13.59	956.4	0.984	82.9
557	D_15_01801_DT_CS	63	0.491	Tissue DNA	Lung cancer	46	93.2	15.11	926.4	0.987	24.4
558	D_15_01802_DT_CS	99	1.483	Tissue DNA	Lung cancer	45.5	93.1	16.08	773.7	0.981	87.9
559	D_15_01918_DT_CS	133	1.643	Tissue DNA	Lung cancer	45.5	92	14.89	990	0.987	43.9
560	D_15_01921_DT_CS	150	1.643	Tissue DNA	Lung cancer	45	92.4	20.13	989.4	0.982	64.5
561	D_15_01925_DP_CS	198	1.092	FFPE DNA	Lung cancer	48	92.3	51.79	487.7	0.987	82.0
562	D_15_01939_DT_CS	19	1.339	Tissue DNA	Lung cancer	45	92.7	11.49	1023.6	0.983	NA
563	D_15_01940_DT_CS	23	1.291	Tissue DNA	Lung cancer	44.5	93.3	13.32	974.6	0.979	NA
564	D_15_01941_DT_CS	17	1.320	Tissue DNA	Lung cancer	45	93	13.9	924.7	0.982	NA
565	D_15_01942_DT_CS	66	1.467	Tissue DNA	Lung cancer	46	92.6	12.62	982.2	0.984	56.4
566	D_15_01956_DT_CS	127	1.721	Tissue DNA	Lung cancer	46	92.8	23.54	785.8	0.986	50.0
567	D_15_01957_DT_CS	24	1.152	Tissue DNA	Lung cancer	45.5	92.9	23.51	750.9	0.983	NA
568	D_15_01958_DT_CS	147	1.646	Tissue DNA	Lung cancer	46	92.2	23.47	780	0.987	85.7

569	D_15_01969_DT_CS	26	1.433	Tissue DNA	Lung cancer	46	93.3	24.78	751.7	0.985	38.1
570	D_15_01970_DT_CS	12	1.199	Tissue DNA	Lung cancer	45.5	93.3	30.69	704.1	0.985	22.6
571	D_15_01973_DP_CS	17	1.006	FFPE DNA	Lung cancer	47.5	92.4	24.96	793.2	0.989	27.3
572	D_15_01980_DT_CS	15	1.265	Tissue DNA	Lung cancer	46.5	93.1	22.74	783.3	0.987	NA
573	D_15_01982_DT_CS	134	1.886	Tissue DNA	Lung cancer	46	93	20.18	887.9	0.986	100.0
574	D_15_01984_DT_CS	88	1.217	Tissue DNA	Lung cancer	45	92.5	12.85	1291.8	0.987	56.4
575	D_15_01985_DT_CS	33	1.286	Tissue DNA	Lung cancer	45	92.9	9.59	1032.4	0.984	43.9
576	D_15_01995_DP_CS	149	1.244	FFPE DNA	Lung cancer	46.5	94.2	32.43	669.1	0.985	77.8
577	D_15_01998_DT_CS	58	1.365	Tissue DNA	Lung cancer	45	94.8	34.91	625.2	0.979	30.3
578	D_15_01999_DT_CS	7	0.956	Tissue DNA	Lung cancer	45	94.5	22.91	635.9	0.981	43.9
579	D_15_02042_DP_CS	176	1.886	FFPE DNA	Lung cancer	48.5	94.5	36.62	623.1	0.985	60.6
580	D_15_02045_DT_CS	31	1.117	Tissue DNA	Lung cancer	45	92.3	11.97	992.1	0.984	NA
581	D_15_02046_DT_CS	46	1.444	Tissue DNA	Lung cancer	45	92.8	11.93	992.4	0.985	38.1
582	D_15_02048_DT_CS	102	1.379	Tissue DNA	Lung cancer	45	92.8	12.05	1013.7	0.984	35.3
583	D_15_02049_DT_CS	84	1.475	Tissue DNA	Lung cancer	45	92.7	11.87	1017.4	0.985	54.6
584	D_15_02051_DT_CS	33	1.432	Tissue DNA	Lung cancer	45	93	10.45	1237.4	0.986	24.4
585	D_15_02079_CP_CS	337	0.173	Cultured cell pellet	Lung cancer	NA	NA	29.79	2510.5	NA	38.1
586	D_15_02080_CP_CS	33	1.058	Cultured cell pellet	Lung cancer	NA	NA	12.79	1074.9	NA	NA
587	D_15_02081_CP_CS	414	0.166	Cultured cell pellet	Lung cancer	NA	NA	16.57	1038.5	NA	22.2
588	D_15_02099_DT_CS	17	1.187	Tissue DNA	Lung cancer	45	92.8	12.21	808.4	0.983	NA
589	D_15_02100_DT_CS	70	1.623	Tissue DNA	Lung cancer	45.5	91.5	15.13	1093	0.987	76.5
590	D_15_02123_TS_CS	145	1.635	Frozen Tissue	Lung cancer	46.5	93.5	29.22	833.7	0.964	100.0
591	D_15_02237_DP_CS	70	1.423	FFPE DNA	Lung cancer	55	88.8	30.4	992.5	0.990	85.7
592	D_15_02238_DP_CS	35	1.391	FFPE DNA	Lung cancer	50	89.5	32.46	768.6	0.991	NA
593	D_15_02241_DP_CS	71	1.340	FFPE DNA	Lung cancer	52.5	89.4	39.77	749.3	0.989	94.1
594	D_15_02242_DP_CS	41	1.316	FFPE DNA	Lung cancer	51.5	89.4	48.01	457.5	0.989	21.8
595	D_15_02245_DP_CS	76	1.276	FFPE DNA	Lung cancer	54	88.8	50.72	452.6	0.967	85.7
596	D_15_02365_DT_CS	34	1.320	Tissue DNA	Lung cancer	47	92.2	32.92	708.8	0.989	38.1
597	D_15_02368_DT_CS	41	1.404	Tissue DNA	Lung cancer	46	93.1	37.99	649.3	0.986	85.7
598	D_15_02406_DT_CS	12	1.199	Tissue DNA	Lung cancer	46	91.4	32.64	601.3	0.984	NA
599	D_15_02408_DT_CS	26	1.003	Tissue DNA	Lung cancer	46	91.1	40	585.3	0.985	43.9
600	D_15_02410_DT_CS	52	0.907	Tissue DNA	Lung cancer	46.5	90.8	36.57	565.8	0.983	91.3
601	D_15_02615_DP_CS	65	1.143	FFPE DNA	Lung cancer	52	90.4	25.79	930.5	0.990	94.1
602	D_15_02616_DP_CS	23	0.861	FFPE DNA	Lung cancer	51	89.8	35.62	860.5	0.992	NA
603	D_15_02619_DP_CS	67	1.046	FFPE DNA	Lung cancer	54	89.8	44.16	640.9	0.986	94.1
604	D_15_02620_DP_CS	31	1.000	FFPE DNA	Lung cancer	53	89.7	56.71	413.1	0.986	20.2
605	D_15_02623_DP_CS	72	1.060	FFPE DNA	Lung cancer	54	88.7	58.42	423.1	0.964	94.1
606	D_15_02626_DC_CS	75	1.278	Cell DNA	Lung cancer	46	92	27.01	700.2	0.982	100.0
607	D_15_02892_TS_CS	158	1.678	Fresh Tissue	Lung cancer	46	92.6	14.45	1086.8	0.985	100.0
608	D_15_02893_TS_CS	66	1.290	Fresh Tissue	Lung cancer	45.5	93.1	22.38	808.2	0.985	63.2
609	D_15_02897_DT_CS	51	1.761	Tissue DNA	Lung cancer	46	92	36.1	701.3	0.987	94.1
610	D_15_02898_DT_CS	31	1.262	Tissue DNA	Lung cancer	46.5	91.8	36.96	557.6	0.985	NA
611	D_15_02936_DT_CS	21	1.489	Tissue DNA	Lung cancer	45	92.5	22.09	775.7	0.984	22.2
612	D_15_04028_DT_CS	95	1.449	Tissue DNA	Lung cancer	46	92.4	34.44	637.8	0.984	100.0
613	D_15_04029_DT_CS	152	1.435	Tissue DNA	Lung cancer	46	91.5	36.81	594.9	0.985	77.8
614	D_15_04032_DT_CS	102	1.168	Tissue DNA	Lung cancer	46.5	94.5	32.13	537.3	0.984	70.3
615	D_15_04034_DT_CS	35	1.494	Tissue DNA	Lung cancer	45	94.7	29.59	648.5	0.983	70.3
616	D_15_04107_TS_CS	113	1.842	Fresh Tissue	Lung cancer	45.5	94	18.04	800.6	0.983	100.0
617	D_15_04132_DT_CS	16	0.602	Tissue DNA	Lung cancer	46	94.9	37.03	552	0.982	23.4
618	D_15_04138_DP_CS	19	1.307	FFPE DNA	Lung cancer	48	91.7	26.31	745.6	0.989	23.4
619	D_15_04226_DT_CS	144	1.672	Tissue DNA	Lung cancer	45	94.8	24.32	634.5	0.977	67.6
620	D_15_04227_DT_CS	65	1.143	Tissue DNA	Lung cancer	44	92.3	33.08	582.2	0.980	32.6
621	D_15_04254_DT_CS	87	1.757	Tissue DNA	Lung cancer	45.5	91.9	31.34	649.8	0.984	100.0
622	D_15_04255_DT_CS	14	1.171	Tissue DNA	Lung cancer	46	92.1	26.58	704.9	0.985	27.3
623	D_15_04257_DT_CS	37	1.458	Tissue DNA	Lung cancer	45.5	92	31.56	716.6	0.985	50.7
624	D_15_04258_DP_CS	16	0.951	FFPE DNA	Lung cancer	49	91.6	50.96	469.7	0.989	32.6
625	D_15_04259_DP_CS	131	1.518	FFPE DNA	Lung cancer	47.5	91.8	34.55	687.2	0.988	61.1
626	D_15_04260_DP_CS	143	1.525	FFPE DNA	Lung cancer	51	91	50.79	501.6	0.987	NA
627	D_15_04444_TS_CS	19	0.943	Frozen Tissue	Lung cancer	NA	NA	20.41	1197.9	0.988	25.9
628	D_15_04445_TS_CS	109	1.248	Frozen Tissue	Lung cancer	NA	NA	21.13	1133.4	0.987	85.7
629	D_15_04446_TS_CS	88	1.590	Frozen Tissue	Lung cancer	NA	NA	19.95	1030.7	0.986	29.6
630	D_15_04447_TS_CS	48	1.313	Frozen Tissue	Lung cancer	NA	NA	20.24	1100.2	0.987	38.7

631	D_15_04449_TS_CS	222	1.694	Frozen Tissue	Lung cancer	NA	NA	18.71	1088.8	0.984	51.6
632	D_15_04450_TS_CS	44	1.040	Frozen Tissue	Lung cancer	NA	NA	18.8	1176.7	0.987	38.1
633	D_15_04468_DT_CS	55	0.856	Tissue DNA	Lung cancer	46	91.8	31.65	706.8	0.986	25.9
634	D_15_04470_DT_CS	18	1.461	Tissue DNA	Lung cancer	NA	NA	41.95	681.2	0.986	NA
635	D_15_04471_DT_CS	93	1.201	Tissue DNA	Lung cancer	NA	NA	36.25	824.6	0.987	84.0
636	D_15_04473_DT_CS	125	1.593	Tissue DNA	Lung cancer	NA	NA	39.24	745.7	0.984	90.1
637	D_15_04474_DP_CS	121	1.476	FFPE DNA	Lung cancer	NA	NA	30.49	752.1	0.987	100.0
638	D_15_04478_DP_CS	11	0.860	FFPE DNA	Lung cancer	49	92.5	19.24	955.9	0.991	NA
639	D_15_04486_DP_CS	0	0.000	FFPE DNA	Lung cancer	48	92.2	34.54	579.3	0.988	NA
640	D_15_04499_DT_CS	54	0.463	Tissue DNA	Lung cancer	NA	NA	20.54	1074.8	0.986	26.4
641	D_15_04500_DT_CS	142	1.729	Tissue DNA	Lung cancer	NA	NA	21.11	1024.6	0.981	82.4
642	D_15_04501_DT_CS	86	1.689	Tissue DNA	Lung cancer	45	91.9	16.54	1068.7	0.988	66.7
643	D_15_04502_DT_CS	164	1.866	Tissue DNA	Lung cancer	45	91.8	20.73	955.5	0.986	63.0
644	D_15_04529_DT_CS	27	0.985	Tissue DNA	Lung cancer	46	92.2	17.28	842.7	0.985	NA
645	D_15_04531_DT_CS	130	1.417	Tissue DNA	Lung cancer	46	92	13.43	992.9	0.986	48.5
646	D_15_04532_DP_CS	101	1.418	FFPE DNA	Lung cancer	48	91.6	18.26	1088.8	0.989	44.1
647	D_15_04545_DT_CS	117	1.694	Tissue DNA	Lung cancer	46	92.1	17.95	955.3	0.983	85.1
648	D_15_04797_DP_CS	13	0.859	FFPE DNA	Lung cancer	47	92.1	16.7	912.4	0.987	NA
649	D_15_04811_TS_CS	23	1.366	Frozen Tissue	Lung cancer	46	91.5	13.52	1081	0.986	22.2
650	D_15_04812_TS_CS	97	1.014	Frozen Tissue	Lung cancer	46	91.2	15.88	995	0.986	29.3
651	D_15_04813_TS_CS	27	1.111	Frozen Tissue	Lung cancer	46	91.4	18.69	1241.1	0.990	77.8
652	D_15_04832_DP_CS	38	1.052	FFPE DNA	Lung cancer	47	92	23.44	1032.2	0.989	32.6
653	D_15_04884_DP_CS	140	1.513	FFPE DNA	Lung cancer	47	91.5	11.47	1056	0.989	40.4
654	D_15_04887_DP_CS	37	1.390	FFPE DNA	Lung cancer	47	93.5	48.74	542.4	0.990	94.1
655	D_15_05036_DP_CS	32	1.087	FFPE DNA	Lung cancer	NA	NA	17.57	1074.5	0.990	24.4
656	D_15_05218_DT_CS	110	1.385	Tissue DNA	Lung cancer	NA	NA	14.99	1120.4	NA	85.4
657	15_00274_DP_CS	30	1.205	FFPE DNA	Kidney cancer	48	91.8	22.05	1034.3	0.991	27.3
658	15_00275_DP_CS	87	1.109	FFPE DNA	Kidney cancer	48	91	29.15	919.8	0.992	94.1
659	15_00276_DP_CS	41	1.223	FFPE DNA	Kidney cancer	48	91.2	22.49	1093	0.991	24.4
660	15_00277_DP_CS	18	0.855	FFPE DNA	Kidney cancer	48	91.1	25.1	1057.1	0.992	100.0
661	15_00278_DP_CS	50	1.079	FFPE DNA	Kidney cancer	49	90.7	32.97	793.8	0.991	22.6
662	15_00286_DP_CS	45	1.708	FFPE DNA	Kidney cancer	51	92.5	30.39	677	0.991	50.7
663	15_00287_DP_CS	50	0.905	FFPE DNA	Kidney cancer	49.5	93.3	20.06	834.8	0.990	50.0
664	15_00412_DP_CS	27	1.433	FFPE DNA	Kidney cancer	49.5	92.5	28.29	722.8	0.990	100.0
665	D_15_00804_DP_CS	39	1.296	FFPE DNA	Kidney cancer	50	91.2	14.75	1009.6	0.992	NA
666	D_15_00857_DP_CS	74	1.381	FFPE DNA	Kidney cancer	50	90.8	24.52	901.9	0.991	43.9
667	D_15_00858_DP_CS	32	1.352	FFPE DNA	Kidney cancer	50.5	91	33.69	721.2	0.991	NA
668	D_15_01017_DP_CS	66	1.558	FFPE DNA	Kidney cancer	49	92.2	37.53	727.8	0.991	21.0
669	D_15_01161_DP_CS	47	1.653	FFPE DNA	Kidney cancer	49	92.7	16.55	953.9	0.991	21.2
670	D_15_01163_DP_CS	38	1.534	FFPE DNA	Kidney cancer	50	92	13.3	965.8	0.991	43.8
671	D_15_01164_DP_CS	76	1.240	FFPE DNA	Kidney cancer	48	92.6	18.62	966.2	0.990	70.3
672	D_15_01174_DP_CS	39	1.382	FFPE DNA	Kidney cancer	47	92.2	25.55	949.8	0.991	NA
673	D_15_01272_DP_CS	35	1.623	FFPE DNA	Kidney cancer	49	91.7	18.74	964.6	0.992	33.3
674	D_15_01420_DP_CS	45	1.278	FFPE DNA	Kidney cancer	49	91	35.88	678	0.990	28.6
675	D_15_01422_DP_CS	72	1.279	FFPE DNA	Kidney cancer	53	87.2	46.96	348.6	0.971	21.8
676	D_15_01425_DP_CS	31	1.564	FFPE DNA	Kidney cancer	49	90.6	28.17	820.6	0.991	NA
677	D_15_01429_DP_CS	30	1.438	FFPE DNA	Kidney cancer	48	91.2	28.43	819.8	0.991	22.2
678	D_15_01430_DP_CS	43	1.391	FFPE DNA	Kidney cancer	51	90.3	46.34	524.6	0.990	32.6
679	D_15_01444_DP_CS	31	1.451	FFPE DNA	Kidney cancer	50	90.5	23.73	890.8	0.991	22.2
680	D_15_01445_DP_CS	49	1.523	FFPE DNA	Kidney cancer	49.5	91.9	18.04	899.3	0.991	NA
681	D_15_01446_DP_CS	30	1.400	FFPE DNA	Kidney cancer	49.5	91.5	15.47	966.8	0.991	39.7
682	D_15_01757_DP_CS	33	1.334	FFPE DNA	Kidney cancer	49	91.6	15.17	1043	0.991	27.6
683	D_15_01797_DP_CS	91	1.413	FFPE DNA	Kidney cancer	49	91.7	22.1	978.7	0.990	57.1
684	D_15_02894_TS_CS	17	1.181	Fresh Tissue	Kidney cancer	45.5	93.2	22.68	739.6	0.983	23.4
685	D_15_04108_TS_CS	54	1.220	Fresh Tissue	Kidney cancer	46	94.2	24.44	748.1	0.985	50.0
686	D_15_04451_TS_CS	89	1.070	Frozen Tissue	Kidney cancer	NA	NA	20.71	1058	0.986	93.6
687	D_15_04452_TS_CS	58	1.066	Frozen Tissue	Kidney cancer	NA	NA	15.92	1150.7	0.985	92.5
688	D_15_04814_TS_CS	87	0.795	Frozen Tissue	Kidney cancer	46	91	20.6	1126.2	0.989	83.8
689	D_15_04815_TS_CS	50	1.331	Frozen Tissue	Kidney cancer	46	90.9	18.19	1070.9	0.988	43.9
690	D_15_04997_TS_CS	87	1.044	Frozen Tissue	Kidney cancer	NA	NA	9.18	1130.2	0.984	59.2
691	D_15_04998_TS_CS	42	0.855	Frozen Tissue	Kidney cancer	NA	NA	12.31	1159	0.986	50.0
692	15_00001_DP_CS	116	1.331	FFPE DNA	Gastric cancer	48	90.7	48.31	552.5	0.985	100.0
693	15_00002_DP_CS	8	1.082	FFPE DNA	Gastric cancer	47	90.8	19.45	1029.3	0.989	NA
694	15_00003_DP_CS	62	1.376	FFPE DNA	Gastric cancer	48	90.1	32.23	726.9	0.989	56.4

695	15_00004_DP_CS	122	0.855	FFPE DNA	Gastric cancer	48	89.7	30.4	756.7	0.990	94.1
696	15_00005_DP_CS	109	1.228	FFPE DNA	Gastric cancer	48	90.8	21.09	992.6	0.989	77.8
697	15_00006_DP_CS	65	1.366	FFPE DNA	Gastric cancer	48	90.4	29.85	948.3	0.990	85.7
698	15_00007_DP_CS	66	1.144	FFPE DNA	Gastric cancer	47	90.4	24.43	1014.2	0.989	21.2
699	15_00008_DP_CS	60	1.311	FFPE DNA	Gastric cancer	48.5	93.1	55.48	515.8	0.989	85.7
700	15_00009_DP_CS	159	1.812	FFPE DNA	Gastric cancer	50	92.8	48.52	595.2	0.985	74.8
701	15_00010_DP_CS	47	1.120	FFPE DNA	Gastric cancer	48	93.2	28.13	930.4	0.991	32.6
702	15_00011_DP_CS	70	1.091	FFPE DNA	Gastric cancer	48	93.3	28.22	960.7	0.989	56.4
703	15_00012_DP_CS	93	1.132	FFPE DNA	Gastric cancer	49	92.4	40.8	592.2	0.989	94.1
704	15_00013_DP_CS	95	1.430	FFPE DNA	Gastric cancer	50	92.7	57.18	516	0.986	94.1
705	15_00014_DP_CS	37	0.985	FFPE DNA	Gastric cancer	48	93.3	41.5	811.5	0.987	22.2
706	15_00015_DP_CS	66	1.296	FFPE DNA	Gastric cancer	47	93.4	21.82	1036.7	0.989	32.6
707	15_00016_DP_CS	47	0.941	FFPE DNA	Gastric cancer	48	92.9	21.96	969.3	0.989	27.3
708	15_00017_DP_CS	11	0.760	FFPE DNA	Gastric cancer	49	93	39.47	571.4	0.989	27.3
709	15_00018_DP_CS	103	1.756	FFPE DNA	Gastric cancer	48	93.2	20.53	913.7	0.988	58.4
710	15_00019_DP_CS	28	1.225	FFPE DNA	Gastric cancer	48	92.8	23.2	1019	0.990	NA
711	15_00020_DP_CS	114	1.259	FFPE DNA	Gastric cancer	48	92.6	20.26	1112.3	0.989	85.7
712	15_00021_DP_CS	28	1.018	FFPE DNA	Gastric cancer	48	94.4	22.28	883.2	0.990	27.3
713	15_00022_DP_CS	16	1.247	FFPE DNA	Gastric cancer	48	94	26.41	855	0.990	NA
714	15_00024_DP_CS	19	0.633	FFPE DNA	Gastric cancer	49	93.9	39.16	601.7	0.989	NA
715	15_00025_DP_CS	125	1.575	FFPE DNA	Gastric cancer	48.5	94.1	27.51	804.6	0.990	85.7
716	15_00027_DP_CS	25	0.939	FFPE DNA	Gastric cancer	49.5	93.5	37.41	626.8	0.989	22.2
717	15_00028_DP_CS	0	0.000	FFPE DNA	Gastric cancer	48	94.2	17.54	826.5	0.988	32.9
718	15_00029_DP_CS	142	1.504	FFPE DNA	Gastric cancer	48	93.8	22.41	856	0.989	77.8
719	15_00030_DP_CS	15	1.171	FFPE DNA	Gastric cancer	48	92	25.99	940.3	0.990	27.3
720	15_00069_DP_CS	159	1.531	FFPE DNA	Gastric cancer	49	93.4	33.12	764	0.990	77.8
721	15_00070_DP_CS	34	1.022	FFPE DNA	Gastric cancer	48	93.6	25.9	863.5	0.991	22.2
722	15_00071_DP_CS	24	1.399	FFPE DNA	Gastric cancer	48	93.8	24.74	851.1	0.990	22.2
723	15_00072_DP_CS	80	0.385	FFPE DNA	Gastric cancer	48	94.2	20.96	800.5	0.988	NA
724	15_00073_DP_CS	19	1.237	FFPE DNA	Gastric cancer	53	92	50.82	395.8	0.982	22.2
725	15_00074_DP_CS	112	1.248	FFPE DNA	Gastric cancer	49	93.3	23.94	814.6	0.990	77.8
726	15_00075_DP_CS	30	1.066	FFPE DNA	Gastric cancer	48	93.7	18.69	823.4	0.989	NA
727	15_00076_DP_CS	55	1.102	FFPE DNA	Gastric cancer	48	92.8	21.54	820.4	0.989	38.1
728	15_00077_DP_CS	46	1.245	FFPE DNA	Gastric cancer	48	94.1	22.07	860.9	0.989	56.4
729	15_00078_DP_CS	89	1.124	FFPE DNA	Gastric cancer	50	93	40.67	693.3	0.991	63.2
730	15_00079_DP_CS	25	1.265	FFPE DNA	Gastric cancer	48	93.6	27.97	929.4	0.990	NA
731	15_00080_DP_CS	27	1.414	FFPE DNA	Gastric cancer	49	93.3	22.17	989.7	0.990	22.2
732	15_00081_DP_CS	43	1.035	FFPE DNA	Gastric cancer	48	90.7	20.83	912	0.989	27.3
733	15_00083_DP_CS	16	1.332	FFPE DNA	Gastric cancer	49	90.5	26.08	973.4	0.991	22.2
734	15_00084_DP_CS	26	1.433	FFPE DNA	Gastric cancer	49	90.5	26.16	866.9	0.990	77.8
735	15_00085_DP_CS	160	1.530	FFPE DNA	Gastric cancer	49	91	24.45	971.3	0.990	100.0
736	15_00086_DP_CS	9	1.061	FFPE DNA	Gastric cancer	49	90.4	34.3	714.2	0.990	27.3
737	15_00087_DP_CS	28	1.049	FFPE DNA	Gastric cancer	47.5	90.8	21.4	1049.5	0.990	NA
738	15_00088_DP_CS	12	1.078	FFPE DNA	Gastric cancer	48.5	90.5	18.43	991.8	0.990	22.2
739	15_00093_DP_CS	28	1.330	FFPE DNA	Gastric cancer	49	90	30.7	824.3	0.991	27.4
740	15_00094_DP_CS	16	1.157	FFPE DNA	Gastric cancer	48	90.8	18.15	1023.8	0.990	NA
741	15_00095_DP_CS	40	1.116	FFPE DNA	Gastric cancer	47	91.1	22.17	950.3	0.989	22.7
742	15_00096_DP_CS	28	1.295	FFPE DNA	Gastric cancer	48	90.1	30.87	932.4	0.991	22.2
743	15_00098_DP_CS	12	1.011	FFPE DNA	Gastric cancer	47.5	90.8	22.75	851.8	0.989	21.4
744	15_00099_DP_CS	21	0.977	FFPE DNA	Gastric cancer	49	90.4	32.74	757	0.991	31.5
745	15_00100_DP_CS	12	1.127	FFPE DNA	Gastric cancer	47.5	90.8	27.36	759.2	0.990	NA
746	15_00101_DP_CS	137	1.535	FFPE DNA	Gastric cancer	49	89.5	45.85	591.7	0.990	100.0
747	15_00102_DP_CS	18	1.276	FFPE DNA	Gastric cancer	48	90.8	28.66	861.3	0.990	NA
748	15_00103_DP_CS	39	1.087	FFPE DNA	Gastric cancer	49	90.2	53.02	611	0.990	26.4
749	15_00104_DP_CS	149	1.758	FFPE DNA	Gastric cancer	48	89.4	29.53	936.3	0.990	66.7
750	15_00105_DP_CS	93	1.494	FFPE DNA	Gastric cancer	47.5	90	34.75	939.6	0.991	77.8
751	15_00106_DP_CS	77	1.313	FFPE DNA	Gastric cancer	48	90.7	24.07	926.8	0.990	20.2
752	15_00107_DP_CS	98	1.446	FFPE DNA	Gastric cancer	48	89.8	30.31	906.8	0.991	52.9
753	15_00108_DP_CS	21	0.945	FFPE DNA	Gastric cancer	48	90.8	35.62	754.6	0.991	NA
754	15_00109_DP_CS	47	1.097	FFPE DNA	Gastric cancer	48	90.9	21.14	931.3	0.990	21.8
755	15_00110_DP_CS	18	1.191	FFPE DNA	Gastric cancer	47.5	91	28.06	842.8	0.990	27.3
756	15_00111_DP_CS	124	0.925	FFPE DNA	Gastric cancer	49	90.3	31.7	931.7	0.991	94.1
757	15_00112_DP_CS	103	1.210	FFPE DNA	Gastric cancer	49	89.9	28.54	943.6	0.991	37.1
758	15_00113_DP_CS	50	1.341	FFPE DNA	Gastric cancer	48	90.7	37.27	685.8	0.990	24.9

759	15_00114_DP_CS	7	1.154	FFPE DNA	Gastric cancer	49	89.8	37.37	721.8	0.991	NA
760	15_00115_DP_CS	12	1.445	FFPE DNA	Gastric cancer	47	91.4	29.13	824.9	0.989	NA
761	15_00116_DP_CS	67	1.008	FFPE DNA	Gastric cancer	48	90.4	34.44	757.6	0.991	23.4
762	15_00118_DP_CS	29	1.047	FFPE DNA	Gastric cancer	48	90	24.16	839.6	0.990	27.6
763	15_00119_DP_CS	112	1.381	FFPE DNA	Gastric cancer	49	89.8	23.09	923.5	0.990	63.2
764	15_00120_DP_CS	30	0.703	FFPE DNA	Gastric cancer	49	89.7	55.09	358.7	0.987	25.9
765	15_00124_DP_CS	31	1.265	FFPE DNA	Gastric cancer	50	90.6	63.21	311.3	0.984	NA
766	15_00125_DP_CS	70	0.853	FFPE DNA	Gastric cancer	48	91.4	49.57	489	0.987	70.3
767	15_00126_DP_CS	42	1.228	FFPE DNA	Gastric cancer	50	90.9	58.58	350.7	0.985	77.8
768	15_00128_DP_CS	16	1.143	FFPE DNA	Gastric cancer	49	90.9	57.82	325.9	0.983	22.7
769	15_00129_DP_CS	76	1.195	FFPE DNA	Gastric cancer	48	91.5	58.8	421.6	0.983	85.7
770	15_00130_DP_CS	127	1.308	FFPE DNA	Gastric cancer	49	90.8	61.71	365.3	0.987	100.0
771	15_00131_DP_CS	69	1.399	FFPE DNA	Gastric cancer	49	90.9	45.06	566.6	0.990	100.0
772	15_00134_DP_CS	89	1.080	FFPE DNA	Gastric cancer	48	91.3	51.06	536	0.990	38.1
773	15_00135_DP_CS	30	1.682	FFPE DNA	Gastric cancer	48	91.7	40.37	621.7	0.990	24.4
774	15_00137_DP_CS	55	1.024	FFPE DNA	Gastric cancer	49	91.1	44.06	586.1	0.990	50.0
775	15_00138_DP_CS	30	0.766	FFPE DNA	Gastric cancer	48	90.7	32.29	956.5	0.992	24.6
776	15_00139_DP_CS	101	0.888	FFPE DNA	Gastric cancer	48.5	91	25.51	947.1	0.991	32.6
777	15_00140_DP_CS	25	1.012	FFPE DNA	Gastric cancer	49	90.8	22.99	1024.3	0.991	22.2
778	15_00171_DT_CS	161	1.630	Tissue DNA	Gastric cancer	45	92.3	12.59	1111.1	0.986	NA
779	15_00194_DP_CS	61	1.203	FFPE DNA	Gastric cancer	48	91.5	26.8	933.4	0.990	100.0
780	15_00266_DP_CS	7	1.475	FFPE DNA	Gastric cancer	48	90.3	28.42	851.3	0.992	100.0
781	15_00267_DP_CS	25	0.944	FFPE DNA	Gastric cancer	49	90.5	44.14	583.4	0.991	100.0
782	15_00268_DP_CS	66	1.251	FFPE DNA	Gastric cancer	49.5	90.7	37.9	732.5	0.991	100.0
783	15_00269_DP_CS	99	1.246	FFPE DNA	Gastric cancer	48	91	27.04	884.4	0.990	100.0
784	15_00270_DP_CS	19	1.490	FFPE DNA	Gastric cancer	46	92.1	22.06	1002.8	0.989	NA
785	15_00271_DP_CS	18	0.974	FFPE DNA	Gastric cancer	52	89.1	41.44	609.4	0.991	100.0
786	15_00273_DP_CS	38	1.158	FFPE DNA	Gastric cancer	48	90.3	22.64	990.1	0.991	32.6
787	15_00383_DT_CS	56	1.313	Tissue DNA	Gastric cancer	45	93.9	13.64	894.4	0.985	48.1
788	15_00384_DT_CS	45	1.073	Tissue DNA	Gastric cancer	45.5	94.2	15.06	916.6	0.986	NA
789	15_00385_DT_CS	67	1.540	Tissue DNA	Gastric cancer	45.5	94.2	14.93	845.3	0.985	100.0
790	15_00386_DP_CS	77	1.251	FFPE DNA	Gastric cancer	48	93.7	15.62	965.7	0.988	26.4
791	15_00403_DP_CS	97	1.249	FFPE DNA	Gastric cancer	49	93.4	16.81	901.9	0.990	100.0
792	15_00410_DP_CS	92	1.127	FFPE DNA	Gastric cancer	48	92.6	17.83	874.5	0.989	28.3
793	15_00417_DT_CS	29	1.396	Tissue DNA	Gastric cancer	45.5	93.8	13.91	944	0.987	NA
794	15_00418_DT_CS	25	1.385	Tissue DNA	Gastric cancer	45.5	93.7	14.79	891.9	0.987	NA
795	15_00419_DT_CS	195	1.759	Tissue DNA	Gastric cancer	45	93.5	16.11	905.1	0.986	52.9
796	15_00424_DP_CS	137	1.265	FFPE DNA	Gastric cancer	48	92.2	22.89	963.6	0.989	32.6
797	15_00425_DP_CS	46	1.313	FFPE DNA	Gastric cancer	49	91.2	42	644.9	0.991	100.0
798	15_00426_DP_CS	8	1.213	FFPE DNA	Gastric cancer	49	91	35.74	755.8	0.991	32.6
799	15_00427_DP_CS	67	1.158	FFPE DNA	Gastric cancer	49.5	90.5	29.88	848.8	0.992	94.1
800	15_00428_DP_CS	9	1.215	FFPE DNA	Gastric cancer	47.5	91.9	12.79	975.4	0.990	22.2
801	15_00433_DP_CS	97	1.432	FFPE DNA	Gastric cancer	49	92.6	13.6	1023.3	0.990	59.2
802	15_00434_DP_CS	72	1.407	FFPE DNA	Gastric cancer	51	91.1	16.52	911.2	0.991	50.0
803	15_00435_DP_CS	12	1.119	FFPE DNA	Gastric cancer	51	91.7	13.11	922.9	0.992	32.6
804	15_00439_DP_CS	72	1.312	FFPE DNA	Gastric cancer	52	91.8	44.61	652.5	0.990	100.0
805	CS11_14_02725	21	0.945	FFPE DNA	Gastric cancer	51	92.5	48.13	472.1	0.990	22.7
806	CS11_14_02726	76	1.266	FFPE DNA	Gastric cancer	50	93.8	53.71	305.3	0.939	94.1
807	CS11_14_02727	409	1.761	FFPE DNA	Gastric cancer	51	92.7	31.65	758.2	0.990	100.0
808	CS11_14_02728	54	1.246	FFPE DNA	Gastric cancer	50.5	92.8	33.08	786	0.990	56.4
809	CS11_14_02729	466	0.216	FFPE DNA	Gastric cancer	49.5	92.8	30.53	883.1	0.990	100.0
810	CS11_14_02730	91	1.287	FFPE DNA	Gastric cancer	50	91.7	49.38	504.2	0.989	38.1
811	CS11_14_02734	90	1.278	FFPE DNA	Gastric cancer	49	93.2	32.25	705	0.989	77.8
812	CS11_14_02735	59	1.246	FFPE DNA	Gastric cancer	50	93	58.13	359.8	0.986	77.8
813	CS11_14_02736	11	0.995	FFPE DNA	Gastric cancer	49	92.8	51.79	358	0.988	NA
814	CS11_14_02737	77	1.382	FFPE DNA	Gastric cancer	51	91.8	15.24	937.6	0.991	43.9
815	CS11_14_02738	30	1.394	FFPE DNA	Gastric cancer	49	91.8	26.54	960.1	0.991	22.2
816	CS11_14_02742	93	1.388	FFPE DNA	Gastric cancer	50.5	92.4	49.07	346.7	0.982	88.7
817	CS11_14_02745	142	1.388	FFPE DNA	Gastric cancer	46	94.3	25.57	1055.9	0.980	70.3
818	CS11_14_02750	26	1.012	FFPE DNA	Gastric cancer	50	92.7	55.03	320	0.983	22.2
819	CS11_14_02752	118	1.420	FFPE DNA	Gastric cancer	46	94.6	30.01	1048.8	0.980	77.8
820	CS11_14_02754	103	1.159	FFPE DNA	Gastric cancer	46	94.8	63.46	372.8	0.965	25.0
821	CS11_14_02755	183	1.513	FFPE DNA	Gastric cancer	44.5	94.3	26.68	1165.5	0.979	94.1
822	CS11_14_02757	53	1.429	FFPE DNA	Gastric cancer	48	92.6	49.2	533.5	0.991	43.9

823	CS11_14_02764	62	1.395	FFPE DNA	Gastric cancer	51	92.2	56.51	402.1	0.987	100.0
824	CS11_14_02765	69	1.339	FFPE DNA	Gastric cancer	51	91.6	59.52	320.1	0.980	100.0
825	CS11_14_02766	31	1.554	FFPE DNA	Gastric cancer	44.5	94.1	33.25	976.7	0.978	27.3
826	CS11_14_02767	153	1.480	FFPE DNA	Gastric cancer	52	90.8	23.56	910.1	0.990	100.0
827	CS11_14_02772	145	1.564	FFPE DNA	Gastric cancer	50	92.6	38.98	591.4	0.990	100.0
828	CS11_14_02773	36	0.678	FFPE DNA	Gastric cancer	49	93.2	41.78	662.7	0.991	NA
829	CS11_14_02775	104	1.700	FFPE DNA	Gastric cancer	51	92.1	47.85	484.1	0.982	100.0
830	CS11_14_02777	48	1.537	FFPE DNA	Gastric cancer	51	91.6	33.33	713	0.990	100.0
831	CS11_14_02783	44	1.268	FFPE DNA	Gastric cancer	50	91.8	27.62	843.8	0.991	32.6
832	CS11_14_02784	30	1.460	FFPE DNA	Gastric cancer	51	91.8	29.86	801.2	0.991	22.2
833	CS11_14_02835	38	1.438	DNA ??	Gastric cancer	45	91.8	32.14	834.6	0.987	27.3
834	CS11_14_02844	76	1.370	FFPE DNA	Gastric cancer	51	91.8	27.84	846.2	0.991	24.9
835	CS11_14_02845	209	1.529	FFPE DNA	Gastric cancer	51	91.3	31.69	794.6	0.991	100.0
836	CS11_14_02846	31	1.238	FFPE DNA	Gastric cancer	50	92.5	55.58	448.1	0.987	100.0
837	CS11_14_02847	24	0.815	FFPE DNA	Gastric cancer	51	90.2	48.42	435.8	0.987	NA
838	CS11_14_02848	60	1.390	FFPE DNA	Gastric cancer	51	92	26.93	862.1	0.991	22.7
839	CS11_14_02849	33	1.245	FFPE DNA	Gastric cancer	50	92.3	26.47	982	0.991	27.3
840	CS11_14_02850	47	1.288	FFPE DNA	Gastric cancer	52	90.2	53.6	362.3	0.978	94.1
841	CS11_14_02851	112	1.382	FFPE DNA	Gastric cancer	49.5	92.2	26.72	752.2	0.989	70.3
842	CS11_14_02853	21	1.190	FFPE DNA	Gastric cancer	50.5	91.8	42.96	556.5	0.988	NA
843	CS11_14_02854	36	1.135	FFPE DNA	Gastric cancer	49	92.3	35.44	698.9	0.990	NA
844	CS11_14_02855	35	1.473	FFPE DNA	Gastric cancer	50	91.6	51.29	436.1	0.990	NA
845	CS11_14_02856	34	1.418	FFPE DNA	Gastric cancer	49	93	33.82	653.4	0.987	94.1
846	CS11_14_02858	99	1.338	FFPE DNA	Gastric cancer	51	91.9	23.52	943.8	0.990	43.9
847	CS11_14_02859	30	1.254	FFPE DNA	Gastric cancer	50	92.4	56.88	399	0.988	21.8
848	CS11_14_02860	167	1.765	FFPE DNA	Gastric cancer	50	92	31.16	880.4	0.990	71.8
849	CS11_14_02861	168	0.966	FFPE DNA	Gastric cancer	50	92	32.19	739.7	0.988	82.0
850	CS11_14_02865	53	1.483	FFPE DNA	Gastric cancer	49	93	51.01	400.2	0.981	NA
851	CS11_14_02867	74	1.481	FFPE DNA	Gastric cancer	50	92.6	44.68	659.3	0.989	100.0
852	CS11_14_02868	92	1.556	FFPE DNA	Gastric cancer	50	92.5	57.12	433.1	0.983	100.0
853	CS11_14_02869	15	1.395	FFPE DNA	Gastric cancer	48	93.1	41.95	550.4	0.988	NA
854	CS11_14_02870	160	1.628	FFPE DNA	Gastric cancer	51.5	92	45.43	613.3	0.988	100.0
855	CS11_14_02871	43	1.335	FFPE DNA	Gastric cancer	49	92.1	26.04	849.9	0.991	27.6
856	CS11_14_02872	46	1.360	FFPE DNA	Gastric cancer	50.5	92.5	30.1	853.1	0.990	100.0
857	CS11_14_02873	34	1.363	FFPE DNA	Gastric cancer	50	92.2	49.7	737	0.991	100.0
858	CS11_14_02874	75	1.382	FFPE DNA	Gastric cancer	50	92.1	51.59	470.9	0.987	85.7
859	CS11_14_02875	69	1.404	FFPE DNA	Gastric cancer	50	92.4	27.77	968.7	0.991	32.6
860	CS11_14_02974	48	1.606	FFPE DNA	Gastric cancer	48	93.2	61.3	332.5	0.987	100.0
861	CS11_14_02975	18	1.349	FFPE DNA	Gastric cancer	50	91.3	50.24	475.8	0.990	22.2
862	CS11_14_02976	97	1.444	FFPE DNA	Gastric cancer	49	92.7	36.74	737.4	0.991	100.0
863	CS11_14_02979	31	1.508	FFPE DNA	Gastric cancer	49	92.3	48.75	524	0.989	100.0
864	CS11_14_02980	62	1.518	FFPE DNA	Gastric cancer	50	91.7	52.96	366.4	0.987	100.0
865	CS11_14_02982	25	1.398	FFPE DNA	Gastric cancer	51	91.3	41.71	571.3	0.990	100.0
866	CS11_14_02983	33	1.508	FFPE DNA	Gastric cancer	49	92.7	40.56	657.8	0.991	100.0
867	CS11_14_03013	104	1.109	FFPE DNA	Gastric cancer	50.5	93	58.41	315.7	0.983	100.0
868	CS11_14_03019	36	1.416	FFPE DNA	Gastric cancer	50	94	58.17	399.8	0.983	100.0
869	CS11_14_03033	48	1.596	FFPE DNA	Gastric cancer	49.5	93.8	44.91	531.6	0.991	100.0
870	CS11_14_03034	56	1.536	FFPE DNA	Gastric cancer	49	94	51.55	508.2	0.987	100.0
871	CS11_14_03035	99	1.438	FFPE DNA	Gastric cancer	50	93.2	53.46	421.1	0.990	100.0
872	CS11_14_03040	49	1.448	FFPE DNA	Gastric cancer	49	93.6	51.25	464.4	0.990	100.0
873	CS11_14_03046	100	1.640	FFPE DNA	Gastric cancer	49.5	93.8	37.34	646.8	0.990	100.0
874	CS11_14_03047	49	1.424	FFPE DNA	Gastric cancer	49.5	94.1	53.75	404.8	0.987	100.0
875	CS11_14_03049	98	1.700	FFPE DNA	Gastric cancer	51	93.3	39.5	699.9	0.991	100.0
876	CS11_14_03052	11	1.162	FFPE DNA	Gastric cancer	50	93.2	57.93	411.7	0.987	22.2
877	CS11_14_03078	33	1.673	FFPE DNA	Gastric cancer	50	91.6	41.79	653.8	0.991	70.3
878	CS11_14_03079	32	1.341	FFPE DNA	Gastric cancer	50	92.8	56.76	439.6	0.990	20.2
879	CS11_14_03081	77	1.251	FFPE DNA	Gastric cancer	50	91.4	48.43	589.4	0.991	NA
880	CS11_14_03083	44	1.304	FFPE DNA	Gastric cancer	50.5	92.2	48.68	498	0.989	38.1
881	CS11_14_03084	36	1.311	FFPE DNA	Gastric cancer	50	92.1	34.56	768.6	0.992	25.9
882	CS11_14_03165	32	1.415	FFPE DNA	Gastric cancer	50	92.1	54	464.9	0.991	NA
883	CS11_14_03167	16	1.180	FFPE DNA	Gastric cancer	50	93	60.63	322.9	0.984	27.3
884	CS11_14_03169	33	1.254	FFPE DNA	Gastric cancer	52	92.2	58.38	322.5	0.983	27.6
885	CS11_14_03170	30	1.565	FFPE DNA	Gastric cancer	49	92.3	39.89	494.4	0.989	32.6
886	CS11_14_03171	26	1.400	FFPE DNA	Gastric cancer	50	92.2	40	685.2	0.991	NA

887	CS11_14_03173	40	1.401	FFPE DNA	Gastric cancer	49	92.7	39.2	714.1	0.991	23.4
888	CS11_14_03178	88	1.485	FFPE DNA	Gastric cancer	50.5	93.5	50.69	446.1	0.988	100.0
889	CS11_14_03180	41	1.423	FFPE DNA	Gastric cancer	51	93.1	53.04	448.1	0.989	100.0
890	CS11_14_03182	33	1.242	FFPE DNA	Gastric cancer	49.5	94.1	54.98	405.3	0.988	100.0
891	CS11_14_03183	65	1.223	FFPE DNA	Gastric cancer	50	93.2	51.03	393.6	0.986	100.0
892	CS11_14_03185	68	1.318	FFPE DNA	Gastric cancer	48.5	93.8	61.98	392.2	0.984	100.0
893	CS11_14_03206	120	1.694	FFPE DNA	Gastric cancer	49	93.3	47.48	505.7	0.988	30.1
894	CS11_14_03207	42	1.221	FFPE DNA	Gastric cancer	50	92.8	42.49	685.5	0.991	NA
895	CS11_14_03208	75	1.244	FFPE DNA	Gastric cancer	51	92.5	51.6	485.8	0.988	20.9
896	CS11_14_03209	44	0.650	FFPE DNA	Gastric cancer	49	93.2	53.73	527	0.990	NA
897	CS11_14_03211	50	1.516	FFPE DNA	Gastric cancer	48	93	38.75	682.8	0.989	NA
898	CS11_14_03219	39	1.291	FFPE DNA	Gastric cancer	49	91.8	50.05	465.2	0.988	70.3
899	CS11_14_03220	123	1.280	FFPE DNA	Gastric cancer	49	91.8	50.07	467.4	0.988	94.1
900	CS11_14_03221	10	1.089	FFPE DNA	Gastric cancer	48	92.3	50.25	546.3	0.987	22.2
901	CS11_14_03222	96	1.324	FFPE DNA	Gastric cancer	49	92	54.23	380.5	0.985	70.3
902	CS11_14_03224	31	1.239	FFPE DNA	Gastric cancer	49	91.3	64.04	327.2	0.984	70.3
903	CS11_14_03226	44	1.401	FFPE DNA	Gastric cancer	50	91.2	59.22	447.8	0.989	32.6
904	CS11_14_03228	22	1.032	FFPE DNA	Gastric cancer	51	90.9	55.65	480.8	0.988	22.2
905	CS11_14_03246	37	1.250	FFPE DNA	Gastric cancer	51	91.2	59.64	380.8	0.988	70.3
906	CS11_14_03248	18	1.245	FFPE DNA	Gastric cancer	50.5	91.3	60.74	348.2	0.985	NA
907	CS11_14_03249	16	0.662	FFPE DNA	Gastric cancer	50	91.9	60.21	365.2	0.988	32.6
908	CS11_14_03251	26	0.990	FFPE DNA	Gastric cancer	50	91.5	65.19	314.4	0.987	21.8
909	CS11_14_03252	12	1.517	FFPE DNA	Gastric cancer	49.5	90.6	60.52	442.1	0.989	21.8
910	CS11_14_03253	46	0.784	FFPE DNA	Gastric cancer	50	91.8	51.74	504.8	0.989	70.3
911	CS11_14_03254	123	1.557	FFPE DNA	Gastric cancer	50	91	59.87	373.9	0.985	56.4
912	CS11_14_03255	55	1.345	FFPE DNA	Gastric cancer	52	90.9	59.92	406.7	0.987	100.0
913	CS11_14_03256	81	1.240	FFPE DNA	Gastric cancer	50	92	49.99	556.8	0.989	100.0
914	CS11_14_03257	109	1.186	FFPE DNA	Gastric cancer	50	91.6	41.75	630.1	0.989	85.7
915	CS11_14_03259	129	1.451	FFPE DNA	Gastric cancer	50	92	39.47	694.1	0.990	94.1
916	CS11_14_03262	121	1.850	FFPE DNA	Gastric cancer	50.5	91.8	50.42	493.9	0.989	80.8
917	CS11_14_03263	16	0.865	FFPE DNA	Gastric cancer	50	92.3	43.77	620.5	0.990	22.2
918	CS11_14_03265	16	1.392	FFPE DNA	Gastric cancer	51	91.1	56.71	376.5	0.984	28.6
919	CS11_14_03266	21	1.036	FFPE DNA	Gastric cancer	50	91.8	53.49	479.1	0.988	NA
920	CS11_14_03268	103	1.728	FFPE DNA	Gastric cancer	49	92.4	35.53	737	0.990	73.5
921	CS11_14_03269	16	1.143	FFPE DNA	Gastric cancer	50	91.8	56.82	427.3	0.988	NA
922	CS11_14_03270	33	1.203	FFPE DNA	Gastric cancer	49	92.5	38.77	767.6	0.990	38.1
923	CS11_14_03271	19	1.016	FFPE DNA	Gastric cancer	48.5	92.8	36.23	790.8	0.991	NA
924	CS11_14_03273	13	0.984	FFPE DNA	Gastric cancer	50.5	91.6	51.02	537.7	0.990	27.3
925	CS11_14_03274	28	1.352	FFPE DNA	Gastric cancer	51	91	59.88	336.3	0.984	32.6
926	CS11_14_03275	154	1.783	FFPE DNA	Gastric cancer	51	91.4	50.66	497.7	0.987	61.1
927	CS11_14_03279	32	1.138	FFPE DNA	Gastric cancer	49	91.9	36.22	835.4	0.991	85.7
928	CS11_14_03282	20	1.206	FFPE DNA	Gastric cancer	49	92.2	41.59	617.5	0.990	27.3
929	CS11_14_03283	26	1.378	FFPE DNA	Gastric cancer	51	91.3	42.32	649.7	0.990	20.7
930	CS11_14_03284	34	1.229	FFPE DNA	Gastric cancer	48	92.1	36.1	707.3	0.990	22.2
931	CS11_14_03286	14	1.035	FFPE DNA	Gastric cancer	51	91.5	43.26	691.3	0.991	NA
932	CS11_14_03287	19	1.265	FFPE DNA	Gastric cancer	49	92.3	45.81	525	0.987	32.6
933	CS11_14_03288	20	0.708	FFPE DNA	Gastric cancer	51	90.2	52.15	497.6	0.990	NA
934	CS11_14_03290	68	1.712	FFPE DNA	Gastric cancer	49	92.1	51.53	563	0.986	57.1
935	CS11_14_03291	69	1.430	FFPE DNA	Gastric cancer	50	92.1	29.92	921.2	0.991	56.4
936	CS11_14_03292	138	1.542	FFPE DNA	Gastric cancer	49	91.8	45.84	605.2	0.989	94.1
937	CS11_14_03293	75	1.172	FFPE DNA	Gastric cancer	52	91.3	32.3	658.2	0.989	32.6
938	CS11_14_03294	132	1.311	FFPE DNA	Gastric cancer	50	92	28.77	858.7	0.991	63.2
939	CS11_14_03297	13	1.157	FFPE DNA	Gastric cancer	47	93.1	30.32	787	0.990	21.4
940	CS11_14_03298	29	1.357	FFPE DNA	Gastric cancer	49	92.3	48.93	528.2	0.990	27.3
941	CS11_14_03299	83	1.243	FFPE DNA	Gastric cancer	50.5	91.7	22.72	1024.9	0.990	85.7
942	CS11_14_03300	23	1.500	FFPE DNA	Gastric cancer	51	91.8	28.77	863	0.991	32.6
943	CS11_14_03301	168	0.360	FFPE DNA	Gastric cancer	49	91.8	41.6	634	0.990	NA
944	CS11_14_03302	20	0.746	FFPE DNA	Gastric cancer	51	91.7	30.13	833.7	0.991	22.7
945	CS11_14_03303	25	1.064	FFPE DNA	Gastric cancer	50	92.2	33.48	808.9	0.991	22.2
946	CS11_14_03305	22	1.197	FFPE DNA	Gastric cancer	49	90.2	33.48	744.6	0.991	22.7
947	CS11_14_03307	23	1.008	FFPE DNA	Gastric cancer	51	90	25.84	858.8	0.991	NA
948	CS11_14_03308	115	1.095	FFPE DNA	Gastric cancer	52	89.5	26.38	843	0.991	100.0
949	CS11_14_03328	12	0.918	FFPE DNA	Gastric cancer	50	90.8	53.95	512	0.991	27.3
950	CS11_14_03329	32	0.657	FFPE DNA	Gastric cancer	47.5	91.9	53.05	493.9	0.989	22.2

951	CS11_14_03330	34	1.212	FFPE DNA	Gastric cancer	49	90.9	65.58	316.8	0.982	24.9
952	CS11_14_03331	160	1.804	FFPE DNA	Gastric cancer	50	90	45.53	640.9	0.989	69.1
953	CS11_14_03332	22	1.379	FFPE DNA	Gastric cancer	48	91.7	46.37	597	0.986	22.2
954	CS11_14_03333	39	1.055	FFPE DNA	Gastric cancer	49	91	35.85	713	0.990	63.2
955	CS11_14_03334	44	0.961	FFPE DNA	Gastric cancer	48	91.2	44.34	680.6	0.991	NA
956	CS11_14_03335	20	1.094	FFPE DNA	Gastric cancer	50	90.4	55.63	476.9	0.989	22.2
957	CS11_14_03336	21	1.035	FFPE DNA	Gastric cancer	49	91	41.09	775.9	0.991	22.2
958	CS11_14_03337	40	1.126	FFPE DNA	Gastric cancer	47	91.9	35.63	817.2	0.989	27.3
959	CS11_14_03338	69	1.189	FFPE DNA	Gastric cancer	48	91.5	42.47	722.1	0.990	38.1
960	CS11_14_03339	31	1.310	FFPE DNA	Gastric cancer	47.5	91.8	43.29	686.9	0.988	NA
961	CS11_14_03340	21	1.472	FFPE DNA	Gastric cancer	50	91.2	38.98	714.9	0.991	NA
962	CS11_14_03341	143	1.389	FFPE DNA	Gastric cancer	47.5	91.8	63.38	434.6	0.984	70.3
963	CS11_14_03342	34	1.162	FFPE DNA	Gastric cancer	47	91.4	43.65	821.7	0.988	NA
964	CS11_14_03343	62	1.164	FFPE DNA	Gastric cancer	49.5	91.1	60.02	453.6	0.987	77.8
965	CS11_14_03344	18	0.684	FFPE DNA	Gastric cancer	55	87.6	49.54	478.6	0.982	NA
966	CS11_14_03345	120	1.583	FFPE DNA	Gastric cancer	51	91.4	42.09	799.8	0.989	69.3
967	CS11_14_03346	88	1.500	FFPE DNA	Gastric cancer	49	91.5	65.86	307.5	0.981	100.0
968	CS11_14_03347	44	1.163	FFPE DNA	Gastric cancer	51	90.4	62.13	423.8	0.988	85.7
969	CS11_14_03348	19	0.951	FFPE DNA	Gastric cancer	50	90.9	39.9	719.2	0.990	38.1
970	CS11_14_03349	44	0.886	FFPE DNA	Gastric cancer	49	91.2	39.79	615.5	0.991	63.2
971	CS11_14_03350	15	1.194	FFPE DNA	Gastric cancer	49	91.1	31.5	699.1	0.991	38.1
972	CS11_14_03351	75	1.533	FFPE DNA	Gastric cancer	53	90.2	41.59	572	0.986	28.7
973	CS11_14_03352	20	1.161	FFPE DNA	Gastric cancer	51	90.6	32.09	803.7	0.991	NA
974	CS11_14_03353	76	1.354	FFPE DNA	Gastric cancer	50	90.8	44.84	554.7	0.990	85.7
975	CS11_14_03354	82	1.524	FFPE DNA	Gastric cancer	50	91.1	39.69	670.3	0.991	40.0
976	CS11_14_03355	76	1.234	FFPE DNA	Gastric cancer	51	90.8	33.5	727.5	0.990	100.0
977	CS11_14_03356	96	1.441	FFPE DNA	Gastric cancer	49	91.5	31.9	925.7	0.991	100.0
978	CS11_14_03357	119	1.415	FFPE DNA	Gastric cancer	50	91.4	52.01	573.8	0.991	94.1
979	CS11_14_03373	132	1.547	FFPE DNA	Gastric cancer	48	91.9	49.17	657.6	0.990	38.3
980	CS11_14_03374	9	0.637	FFPE DNA	Gastric cancer	50	91.3	45.91	821	0.990	NA
981	CS11_14_03375	37	1.272	FFPE DNA	Gastric cancer	50	90.9	33.47	927.9	0.991	24.9
982	CS11_14_03377	51	1.202	FFPE DNA	Gastric cancer	48	91.8	45.14	657.3	0.990	22.2
983	CS11_14_03379	29	1.334	FFPE DNA	Gastric cancer	51	90.2	46.6	516	0.989	21.8
984	CS11_14_03380	59	1.078	FFPE DNA	Gastric cancer	50	91.3	38.11	687.8	0.991	70.3
985	CS11_14_03382	119	1.335	FFPE DNA	Gastric cancer	49.5	91.7	56.84	435	0.983	77.8
986	CS11_14_03383	27	1.293	FFPE DNA	Gastric cancer	51	90.8	38.74	763.1	0.991	38.1
987	CS11_14_03384	147	1.513	FFPE DNA	Gastric cancer	49	91.2	37.01	762.2	0.987	100.0
988	CS11_14_03385	145	1.470	FFPE DNA	Gastric cancer	49	90.8	29.31	806.5	0.990	63.2
989	CS11_14_03386	132	0.955	FFPE DNA	Gastric cancer	48	91.6	25.55	969.9	0.990	52.4
990	CS11_14_03387	53	1.552	FFPE DNA	Gastric cancer	50	90.8	32.31	836.9	0.991	85.7
991	CS11_14_03389	60	0.967	FFPE DNA	Gastric cancer	49	91	31.32	779.3	0.991	77.8
992	CS11_14_03390	85	1.367	FFPE DNA	Gastric cancer	48	91	24.67	872.3	0.990	40.0
993	CS11_14_03391	19	0.951	FFPE DNA	Gastric cancer	48	91.8	44.14	661.7	0.989	NA
994	CS11_14_03392	116	1.266	FFPE DNA	Gastric cancer	50	90.5	29.56	801.5	0.990	70.3
995	CS11_14_03393	8	1.494	FFPE DNA	Gastric cancer	48.5	91.3	30.67	960.4	0.991	NA
996	CS11_14_03394	13	1.157	FFPE DNA	Gastric cancer	50	90.7	32.36	862.2	0.991	22.2
997	CS11_14_03395	41	1.065	FFPE DNA	Gastric cancer	50	90.9	30.31	784	0.990	25.9
998	CS11_14_03396	12	1.075	FFPE DNA	Gastric cancer	49.5	90.9	21.47	977.7	0.990	21.4
999	CS11_14_03397	140	1.671	FFPE DNA	Gastric cancer	50	91	27.38	893	0.990	64.9
1000	CS11_14_03398	41	0.868	FFPE DNA	Gastric cancer	48	91	34.59	715.2	0.989	25.9
1001	CS11_14_03399	12	1.265	FFPE DNA	Gastric cancer	49	90.8	33.64	701.2	0.990	22.2
1002	CS11_14_03400	18	1.259	FFPE DNA	Gastric cancer	48	89.8	23.73	867.3	0.989	22.2
1003	CS11_14_03401	23	1.485	FFPE DNA	Gastric cancer	49	91.1	28.06	835.7	0.989	33.3
1004	CS11_14_03402	23	1.318	FFPE DNA	Gastric cancer	48.5	91.2	23.58	1119.9	0.990	24.4
1005	CS11_14_03403	85	1.133	FFPE DNA	Gastric cancer	50	93.4	44.81	450.4	0.986	85.7
1006	CS11_14_03404	13	1.264	FFPE DNA	Gastric cancer	50	89.7	25.42	904.6	0.990	NA
1007	CS11_14_03405	32	0.999	FFPE DNA	Gastric cancer	49	90.2	25.48	995	0.989	22.7
1008	CS11_14_03406	8	1.213	FFPE DNA	Gastric cancer	49	89.8	24.85	883	0.990	22.2
1009	CS11_14_03407	10	1.280	FFPE DNA	Gastric cancer	51	89.4	39.07	621.5	0.990	NA
1010	CS11_14_03408	58	1.054	FFPE DNA	Gastric cancer	49	90.1	28.5	804.5	0.990	43.9
1011	CS11_14_03409	10	1.366	FFPE DNA	Gastric cancer	49	89.9	41.15	473.3	0.987	NA
1012	CS11_14_03410	98	1.337	FFPE DNA	Gastric cancer	50	90.1	31.24	847.9	0.990	94.1
1013	CS11_14_03412	20	1.392	FFPE DNA	Gastric cancer	50	89.3	36.98	792.6	0.991	22.2
1014	CS11_14_03413	20	1.051	FFPE DNA	Gastric cancer	50	89.8	32.27	799.9	0.991	22.2

1015	CS11_14_03414	17	1.141	FFPE DNA	Gastric cancer	49	90.8	26.95	876	0.990	NA
1016	CS11_14_03416	24	1.280	FFPE DNA	Gastric cancer	50	89.4	33.84	695.6	0.990	27.3
1017	CS11_14_03418	119	1.446	FFPE DNA	Gastric cancer	48	90.5	25.63	940	0.990	94.1
1018	CS11_14_03419	9	1.273	FFPE DNA	Gastric cancer	48.5	91	26.35	895	0.991	22.2
1019	CS11_14_03420	28	1.351	FFPE DNA	Gastric cancer	49	90	27.34	869.2	0.990	25.9
1020	CS11_14_03421	10	0.802	FFPE DNA	Gastric cancer	50	89.4	33.09	832.2	0.991	NA
1021	CS11_14_03422	47	1.006	FFPE DNA	Gastric cancer	48	90.4	33.87	733.5	0.990	32.6
1022	CS11_14_03423	178	1.234	FFPE DNA	Gastric cancer	49	89.7	28.31	947.3	0.990	100.0
1023	CS11_14_03424	54	1.169	FFPE DNA	Gastric cancer	49	90.2	37.58	704.4	0.989	100.0
1024	CS11_14_03425	39	1.239	FFPE DNA	Gastric cancer	49.5	92.2	32.7	746.5	0.991	50.0
1025	CS11_14_03426	26	0.810	FFPE DNA	Gastric cancer	50	92.3	28.97	779.6	0.991	32.6
1026	CS11_14_03427	15	1.083	FFPE DNA	Gastric cancer	49	92.5	31.1	792.7	0.991	56.4
1027	CS11_14_03428	63	0.826	FFPE DNA	Gastric cancer	48	93.2	29.11	844.3	0.990	70.3
1028	CS11_14_03429	36	1.230	FFPE DNA	Gastric cancer	48	91.2	38.44	648.5	0.990	25.9
1029	CS11_14_03430	17	1.518	FFPE DNA	Gastric cancer	50	89.4	31.07	906.7	0.992	27.3
1030	CS11_14_03431	62	1.139	FFPE DNA	Gastric cancer	49	90.6	33.34	897.5	0.991	21.0
1031	CS11_14_03433	121	1.550	FFPE DNA	Gastric cancer	48	91.7	35.5	867.3	0.989	59.5
1032	CS11_14_03434	82	0.909	FFPE DNA	Gastric cancer	48	91	44.51	688.9	0.990	38.1
1033	CS11_14_03436	40	1.309	FFPE DNA	Gastric cancer	50	90.8	21.95	907.3	0.990	32.6
1034	CS11_14_03437	13	1.119	FFPE DNA	Gastric cancer	49	91.5	30.59	834.9	0.991	25.9
1035	CS11_14_03447	17	1.181	FFPE DNA	Gastric cancer	49	91.2	30.88	765.2	0.990	27.3
1036	CS11_14_03448	103	1.175	FFPE DNA	Gastric cancer	49.5	91.2	23.04	1023.5	0.990	70.3
1037	CS11_14_03449	89	1.348	FFPE DNA	Gastric cancer	50	90.9	30.25	837.7	0.990	63.2
1038	CS11_14_03451	28	1.213	FFPE DNA	Gastric cancer	49	91.6	27.06	674.9	0.988	23.4
1039	CS11_14_03452	133	1.676	FFPE DNA	Gastric cancer	48	91.8	29.61	1058.5	0.991	55.1
1040	CS11_14_03453	29	1.041	FFPE DNA	Gastric cancer	48	92.1	28.28	873.2	0.990	NA
1041	CS11_14_03454	35	1.387	FFPE DNA	Gastric cancer	52	90.4	40.83	584.9	0.990	27.3
1042	CS11_14_03455	22	1.169	FFPE DNA	Gastric cancer	49	91.2	22.83	879.8	0.989	NA
1043	CS11_14_03456	30	0.900	FFPE DNA	Gastric cancer	48	91.6	28.67	810.1	0.989	22.7
1044	CS11_14_03457	49	1.266	FFPE DNA	Gastric cancer	49	91.2	22.18	876.1	0.990	32.6
1045	CS11_14_03458	108	1.497	FFPE DNA	Gastric cancer	48	92	25.56	904.5	0.989	35.7
1046	CS11_14_03459	107	1.819	FFPE DNA	Gastric cancer	51	91	19.58	983	0.989	60.0
1047	CS11_14_03460	59	0.690	FFPE DNA	Gastric cancer	50	91	29.69	844.3	0.990	56.4
1048	CS11_14_03461	93	1.439	FFPE DNA	Gastric cancer	49	91.3	33.68	729.5	0.990	94.1
1049	CS11_14_03462	140	1.268	FFPE DNA	Gastric cancer	49	91.7	39.93	756.1	0.990	100.0
1050	CS11_14_03463	76	1.169	FFPE DNA	Gastric cancer	47.5	92.2	40.36	650.7	0.988	70.3
1051	CS11_14_03464	64	1.086	FFPE DNA	Gastric cancer	48.5	91.5	23.69	927.6	0.989	NA
1052	CS11_14_03465	22	1.053	FFPE DNA	Gastric cancer	48	92	29.71	875.3	0.990	NA
1053	CS11_14_03466	36	0.878	FFPE DNA	Gastric cancer	49	89.6	35.06	985.2	0.990	NA
1054	CS11_14_03467	18	0.934	FFPE DNA	Gastric cancer	49	90.2	26.08	989.7	0.990	NA
1055	CS11_14_03468	13	1.091	FFPE DNA	Gastric cancer	48	90.4	45.1	710.3	0.989	22.2
1056	CS11_14_03469	26	1.091	FFPE DNA	Gastric cancer	49	90.5	46.85	753.6	0.989	NA
1057	CS11_14_03470	19	1.279	FFPE DNA	Gastric cancer	47.5	91	28.21	902.4	0.990	32.6
1058	CS11_14_03471	21	0.910	FFPE DNA	Gastric cancer	49	90.7	41.46	801.8	0.988	21.8
1059	CS11_14_03472	11	1.121	FFPE DNA	Gastric cancer	49	89.4	42.57	846.8	0.991	27.3
1060	CS11_14_03473	18	0.958	FFPE DNA	Gastric cancer	50	89.2	36.26	674.7	0.990	22.2
1061	CS11_14_03474	42	1.347	FFPE DNA	Gastric cancer	49	89.7	34.9	867.9	0.991	77.8
1062	CS11_14_03475	13	0.859	FFPE DNA	Gastric cancer	48	91.3	20.36	1067.1	0.989	NA
1063	CS11_14_03476	107	1.034	FFPE DNA	Gastric cancer	49	90.4	28.9	938.8	0.991	22.7
1064	D_15_00765_DP_CS	12	0.888	FFPE DNA	Gastric cancer	50	92.2	17.82	1005.7	0.992	27.3
1065	D_15_00766_DP_CS	5	1.332	FFPE DNA	Gastric cancer	49	92.6	18.19	929.6	0.992	22.2
1066	D_15_00767_DP_CS	114	1.505	FFPE DNA	Gastric cancer	49	92.2	17.56	990	0.992	55.2
1067	D_15_00799_DP_CS	217	1.702	FFPE DNA	Gastric cancer	51.5	90.8	48.41	457.3	0.988	70.3
1068	D_15_00800_DP_CS	33	1.356	FFPE DNA	Gastric cancer	47	93.2	26.28	1028	0.990	32.6
1069	D_15_00810_DP_CS	101	1.544	FFPE DNA	Gastric cancer	49	91.8	44.9	532.3	0.987	72.0
1070	D_15_00813_DP_CS	56	1.306	FFPE DNA	Gastric cancer	47	92.5	10.82	962.9	0.987	27.3
1071	D_15_00814_DP_CS	71	1.162	FFPE DNA	Gastric cancer	48	92.2	13.28	971	0.989	85.7
1072	D_15_00852_DP_CS	106	1.479	FFPE DNA	Gastric cancer	45	92.8	30.35	705	0.984	100.0
1073	D_15_00866_DP_CS	64	1.525	FFPE DNA	Gastric cancer	49	91.3	21.42	978.1	0.990	28.6
1074	D_15_00867_DP_CS	37	1.351	FFPE DNA	Gastric cancer	50	90.6	26.9	772	0.992	27.3
1075	D_15_00868_DP_CS	91	1.441	FFPE DNA	Gastric cancer	49	91.8	27.45	822.4	0.991	38.1
1076	D_15_00872_DT_CS	83	1.429	Tissue DNA	Gastric cancer	45.5	92.3	21.82	850.6	0.987	38.1
1077	D_15_00916_TS_CS	130	1.250	Frozen Tissue	Gastric cancer	45	91.1	15.14	1001.4	0.985	76.5
1078	D_15_00917_TS_CS	167	1.818	Frozen Tissue	Gastric cancer	45	91.5	16.57	923.6	0.982	100.0

1079	D_15_00918_TS_CS	97	1.535	Frozen Tissue	Gastric cancer	45	91.1	21.97	950.2	0.985	64.2
1080	D_15_00919_TS_CS	196	1.789	Frozen Tissue	Gastric cancer	45.5	90.8	15.97	997.5	0.985	100.0
1081	D_15_00920_TS_CS	38	1.069	Frozen Tissue	Gastric cancer	45	91.4	17.94	1037.1	0.984	88.7
1082	D_15_00921_TS_CS	135	1.621	Frozen Tissue	Gastric cancer	45.5	90.6	16.1	1037.3	0.986	64.9
1083	D_15_01008_DP_CS	24	1.524	FFPE DNA	Gastric cancer	48	92.1	43.08	619	0.990	24.4
1084	D_15_01019_DT_CS	32	1.330	Tissue DNA	Gastric cancer	46	93.2	31.85	794.4	0.989	NA
1085	D_15_01020_DT_CS	146	1.268	Tissue DNA	Gastric cancer	46	93.9	32.21	774	0.989	77.8
1086	D_15_01022_DT_CS	119	1.564	Tissue DNA	Gastric cancer	45.5	93	17.78	929	0.988	77.8
1087	D_15_01025_DT_CS	33	1.177	Tissue DNA	Gastric cancer	46	94	35.19	702.4	0.987	NA
1088	D_15_01086_DP_CS	36	1.433	FFPE DNA	Gastric cancer	51	91.1	16.04	921.1	0.992	27.3
1089	D_15_01087_DP_CS	19	1.430	FFPE DNA	Gastric cancer	46	93.3	34.25	794.4	0.986	22.2
1090	D_15_01088_DP_CS	40	1.535	FFPE DNA	Gastric cancer	48.5	92	22.87	849.3	0.991	NA
1091	D_15_01092_DT_CS	248	1.695	Tissue DNA	Gastric cancer	45.5	92.5	25.03	840.4	0.985	46.1
1092	D_15_01109_DT_CS	53	1.475	Tissue DNA	Gastric cancer	45	92.9	14.26	988.8	0.985	85.7
1093	D_15_01110_DT_CS	73	1.567	Tissue DNA	Gastric cancer	45	92.7	12.49	1083.9	0.985	42.1
1094	D_15_01148_DP_CS	27	1.133	FFPE DNA	Gastric cancer	48	92.1	11.18	916	0.989	85.7
1095	D_15_01149_DP_CS	30	1.063	FFPE DNA	Gastric cancer	48.5	92.3	15.36	1039.4	0.991	35.7
1096	D_15_01150_DP_CS	157	0.847	FFPE DNA	Gastric cancer	48	92	17.23	803.5	0.995	85.8
1097	D_15_01151_DP_CS	21	0.953	FFPE DNA	Gastric cancer	48	92	12.03	989.1	0.990	NA
1098	D_15_01159_DP_CS	43	1.426	FFPE DNA	Gastric cancer	50.5	92	29.65	817.6	0.991	NA
1099	D_15_01166_DP_CS	146	1.584	FFPE DNA	Gastric cancer	50	92.2	25.67	874.5	0.991	100.0
1100	D_15_01216_DT_CS	74	1.826	Tissue DNA	Gastric cancer	45	92.6	16.1	960.8	0.987	54.0
1101	D_15_01217_DT_CS	31	1.419	Tissue DNA	Gastric cancer	46	92.4	18.79	969.8	0.988	27.3
1102	D_15_01218_DT_CS	10	0.950	Tissue DNA	Gastric cancer	45.5	92.3	15.85	977.7	0.989	32.6
1103	D_15_01232_DT_CS	38	1.355	Tissue DNA	Gastric cancer	45	92.8	16.17	970.3	0.988	32.6
1104	D_15_01273_DP_CS	144	1.338	FFPE DNA	Gastric cancer	48	91.6	23.34	784.3	0.990	70.3
1105	D_15_01431_DP_CS	27	1.195	FFPE DNA	Gastric cancer	47	91.5	23.61	1156.3	0.991	43.9
1106	D_15_01432_DP_CS	24	1.471	FFPE DNA	Gastric cancer	49	91	39.73	645.1	0.988	NA
1107	D_15_01437_DT_CS	87	1.499	Tissue DNA	Gastric cancer	45	93.2	14.14	748.9	0.980	33.3
1108	D_15_01440_DT_CS	55	1.512	Tissue DNA	Gastric cancer	45	93.8	14.21	879.2	0.985	NA
1109	D_15_01448_DP_CS	95	1.787	FFPE DNA	Gastric cancer	50	91	21.3	990.5	0.991	59.2
1110	D_15_01450_DP_CS	25	1.124	FFPE DNA	Gastric cancer	49.5	91.5	18.73	1147.4	0.992	22.2
1111	D_15_01451_DP_CS	74	1.586	FFPE DNA	Gastric cancer	48	92.2	19.02	865.9	0.990	32.6
1112	D_15_01460_DT_CS	70	1.335	Tissue DNA	Gastric cancer	45.5	92.1	32.61	865.1	0.988	94.1
1113	D_15_01464_DP_CS	116	1.488	FFPE DNA	Gastric cancer	53	91.6	46.03	487.8	0.981	94.1
1114	D_15_01465_DP_CS	20	1.470	FFPE DNA	Gastric cancer	47	92.5	22.6	984.9	0.991	NA
1115	D_15_01467_DP_CS	23	1.423	FFPE DNA	Gastric cancer	47	92.4	21.75	971	0.992	NA
1116	D_15_01470_DP_CS	19	1.608	FFPE DNA	Gastric cancer	47	92.7	25.49	1004.2	0.992	32.6
1117	D_15_01474_DP_CS	28	1.304	FFPE DNA	Gastric cancer	47.5	92.2	22.14	935.6	0.991	NA
1118	D_15_01497_DP_CS	23	1.337	FFPE DNA	Gastric cancer	NA	NA	66.88	328.9	0.987	32.6
1119	D_15_01498_DP_CS	67	1.497	FFPE DNA	Gastric cancer	NA	NA	62.71	419.8	0.988	100.0
1120	D_15_01499_DP_CS	41	1.006	FFPE DNA	Gastric cancer	NA	NA	47.57	643.2	0.991	27.3
1121	D_15_01501_DP_CS	44	1.405	FFPE DNA	Gastric cancer	NA	NA	50.66	555.5	0.985	94.1
1122	D_15_01502_DP_CS	74	1.499	FFPE DNA	Gastric cancer	NA	NA	44.94	680.4	0.991	42.1
1123	D_15_01503_DP_CS	29	1.285	FFPE DNA	Gastric cancer	NA	NA	42.87	618.3	0.991	27.3
1124	D_15_01504_DP_CS	169	1.357	FFPE DNA	Gastric cancer	48.5	92.7	48.18	588.4	0.991	70.3
1125	D_15_01505_DP_CS	19	0.610	FFPE DNA	Gastric cancer	52	91.6	43.97	516.9	0.991	25.9
1126	D_15_01506_DP_CS	26	1.172	FFPE DNA	Gastric cancer	48	93.5	48.82	575.2	0.991	22.2
1127	D_15_01507_DP_CS	89	1.512	FFPE DNA	Gastric cancer	47	92.9	42	413.4	0.982	38.1
1128	D_15_01508_DP_CS	30	1.043	FFPE DNA	Gastric cancer	48	93	36.87	817.9	0.992	NA
1129	D_15_01509_DP_CS	94	0.965	FFPE DNA	Gastric cancer	47	92.8	29.04	756.9	0.991	25.9
1130	D_15_01510_DP_CS	61	1.489	FFPE DNA	Gastric cancer	47	92.7	40.2	549.9	0.988	41.3
1131	D_15_01511_DP_CS	118	1.300	FFPE DNA	Gastric cancer	48	92.5	30.1	944.5	0.992	32.6
1132	D_15_01512_DP_CS	165	1.755	FFPE DNA	Gastric cancer	51	90.4	50.36	414.7	0.987	91.3
1133	D_15_01513_DP_CS	94	1.529	FFPE DNA	Gastric cancer	49	91.4	36.38	522	0.989	43.9
1134	D_15_01574_DP_CS	43	1.469	FFPE DNA	Gastric cancer	49	91.9	12.6	1114.7	0.988	NA
1135	D_15_01575_DP_CS	34	1.353	FFPE DNA	Gastric cancer	50.5	91.1	14.7	1120.9	0.988	22.7
1136	D_15_01576_DP_CS	22	1.213	FFPE DNA	Gastric cancer	48	91.5	14.3	994.1	0.987	32.6
1137	D_15_01578_DP_CS	50	1.783	FFPE DNA	Gastric cancer	49	91.2	13.89	888.1	0.986	22.2
1138	D_15_01580_DP_CS	45	1.293	FFPE DNA	Gastric cancer	48.5	91.7	17.28	877.9	0.986	32.6
1139	D_15_01581_DP_CS	29	1.393	FFPE DNA	Gastric cancer	50	91.7	15.05	897.9	0.984	27.3
1140	D_15_01582_DP_CS	74	1.834	FFPE DNA	Gastric cancer	47.5	92.3	11.45	1017.1	0.987	27.3
1141	D_15_01583_DP_CS	31	1.305	FFPE DNA	Gastric cancer	48.5	91.5	14.01	1202.2	0.989	NA
1142	D_15_01584_DP_CS	63	1.481	FFPE DNA	Gastric cancer	49.5	91.2	14.5	1086.1	0.990	32.6

1143	D_15_01585_DP_CS	44	1.231	FFPE DNA	Gastric cancer	52	90.4	29.95	930	0.990	38.1
1144	D_15_01586_DP_CS	62	0.918	FFPE DNA	Gastric cancer	51	90.8	30.97	860.1	0.990	32.6
1145	D_15_01587_DP_CS	35	1.358	FFPE DNA	Gastric cancer	51	90.2	30.9	864.7	0.992	27.3
1146	D_15_01588_DP_CS	33	1.380	FFPE DNA	Gastric cancer	51.5	90.2	25.53	1034.2	0.991	NA
1147	D_15_01589_DP_CS	69	0.722	FFPE DNA	Gastric cancer	51	90.4	30.1	923.2	0.992	32.6
1148	D_15_01590_DP_CS	23	1.444	FFPE DNA	Gastric cancer	51	89.9	25.58	992.3	0.992	NA
1149	D_15_01591_DP_CS	90	1.038	FFPE DNA	Gastric cancer	50	90.8	28.01	997.4	0.992	NA
1150	D_15_01592_DP_CS	114	0.529	FFPE DNA	Gastric cancer	50	90.8	29.87	923.8	0.991	NA
1151	D_15_01594_DP_CS	30	1.399	FFPE DNA	Gastric cancer	50.5	90.4	25.69	906	0.992	100.0
1152	D_15_01595_DP_CS	73	1.660	FFPE DNA	Gastric cancer	52	90.6	21.95	1065.2	0.991	43.9
1153	D_15_01596_DP_CS	32	1.425	FFPE DNA	Gastric cancer	52	90.3	21.55	1034.1	0.990	NA
1154	D_15_01597_DP_CS	61	1.453	FFPE DNA	Gastric cancer	53	89.8	26.66	941.9	0.991	NA
1155	D_15_01598_DP_CS	201	1.806	FFPE DNA	Gastric cancer	51	89.4	15.53	1160.7	0.989	85.7
1156	D_15_01599_DP_CS	43	1.672	FFPE DNA	Gastric cancer	50.5	91	20.63	1047.2	0.990	22.2
1157	D_15_01600_DP_CS	60	1.300	FFPE DNA	Gastric cancer	52	90.2	16.84	1032.1	0.990	27.3
1158	D_15_01601_DP_CS	26	1.306	FFPE DNA	Gastric cancer	52	92.3	18.19	1029.3	0.988	22.2
1159	D_15_01602_DP_CS	30	1.370	FFPE DNA	Gastric cancer	51	92.2	18.71	1077	0.990	22.2
1160	D_15_01603_DP_CS	41	1.278	FFPE DNA	Gastric cancer	52	92.2	22.24	1025.9	0.989	NA
1161	D_15_01604_DP_CS	28	1.281	FFPE DNA	Gastric cancer	52	92	17.32	973.8	0.991	22.2
1162	D_15_01605_DP_CS	102	1.389	FFPE DNA	Gastric cancer	52	92.3	18.33	1036.6	0.989	32.6
1163	D_15_01606_DP_CS	34	1.149	FFPE DNA	Gastric cancer	49.5	92.6	24.46	1030	0.990	22.2
1164	D_15_01607_DP_CS	31	1.375	FFPE DNA	Gastric cancer	52	92.3	27.72	889.6	0.989	27.3
1165	D_15_01608_DP_CS	65	1.277	FFPE DNA	Gastric cancer	50	92.8	19.09	1017.2	0.988	32.6
1166	D_15_01611_DP_CS	49	1.455	FFPE DNA	Gastric cancer	50	92.5	27.36	900.3	0.988	22.2
1167	D_15_01612_DP_CS	39	1.341	FFPE DNA	Gastric cancer	52	92.1	18.98	925.2	0.987	27.3
1168	D_15_01613_DP_CS	25	1.361	FFPE DNA	Gastric cancer	52	91.3	28.32	1109.5	0.992	100.0
1169	D_15_01614_DP_CS	124	1.437	FFPE DNA	Gastric cancer	52	91.8	18.89	915.1	0.991	77.8
1170	D_15_01615_DP_CS	28	1.468	FFPE DNA	Gastric cancer	53	91.9	22.11	922.9	0.989	27.3
1171	D_15_01616_DP_CS	41	1.325	FFPE DNA	Gastric cancer	52	92.2	17.53	944.9	0.989	22.2
1172	D_15_01617_DP_CS	44	1.371	FFPE DNA	Gastric cancer	52	91.8	18.97	969	0.989	20.2
1173	D_15_01618_DP_CS	32	1.451	FFPE DNA	Gastric cancer	51	92.3	15.15	1171.3	0.991	27.3
1174	D_15_01619_DP_CS	68	1.229	FFPE DNA	Gastric cancer	54	90.8	29.7	778	0.991	27.3
1175	D_15_01620_DP_CS	21	1.463	FFPE DNA	Gastric cancer	53	91.2	25.59	1050.2	0.990	22.2
1176	D_15_01621_DP_CS	161	1.734	FFPE DNA	Gastric cancer	55	89.8	25.44	1014.5	0.987	65.7
1177	D_15_01622_DP_CS	38	1.501	FFPE DNA	Gastric cancer	51	92.2	16.72	940.6	0.989	22.2
1178	D_15_01623_DP_CS	216	0.508	FFPE DNA	Gastric cancer	51	91.7	26.2	837.8	0.989	27.3
1179	D_15_01624_DP_CS	21	1.609	FFPE DNA	Gastric cancer	51	90.1	20.47	1280.4	0.992	22.2
1180	D_15_01625_DP_CS	34	1.367	FFPE DNA	Gastric cancer	50.5	90.5	23	1305.9	0.992	NA
1181	D_15_01626_DP_CS	104	1.683	FFPE DNA	Gastric cancer	51	90.3	25.09	1227.8	0.991	100.0
1182	D_15_01627_DP_CS	92	1.659	FFPE DNA	Gastric cancer	48	92.1	18.8	903.6	0.987	85.7
1183	D_15_01628_DP_CS	37	1.321	FFPE DNA	Gastric cancer	49	91.2	18.7	874.9	0.989	32.6
1184	D_15_01629_DP_CS	48	1.589	FFPE DNA	Gastric cancer	48	92	18.71	868.5	0.986	32.6
1185	D_15_01630_DP_CS	35	1.322	FFPE DNA	Gastric cancer	46	92.5	18.08	889.9	0.981	43.9
1186	D_15_01631_DP_CS	44	1.488	FFPE DNA	Gastric cancer	50	91.5	22.27	816.8	0.984	21.0
1187	D_15_01666_TS_CS	86	1.172	Frozen Tissue	Gastric cancer	45.5	92.9	10.74	1115.1	0.985	31.7
1188	D_15_01667_TS_CS	156	1.419	Frozen Tissue	Gastric cancer	46	92.4	18.44	921	0.985	57.8
1189	D_15_01679_DT_CS	140	1.874	Tissue DNA	Gastric cancer	46	93.7	10.9	1063.7	0.986	66.7
1190	D_15_01753_DP_CS	230	0.803	FFPE DNA	Gastric cancer	47	93.4	27.33	802.5	0.988	88.1
1191	D_15_01755_DP_CS	118	1.308	FFPE DNA	Gastric cancer	51	90.5	13	835.6	0.990	94.1
1192	D_15_01758_DP_CS	29	1.190	FFPE DNA	Gastric cancer	50	90.7	12.43	1087.2	0.991	27.3
1193	D_15_01789_DP_CS	38	0.654	FFPE DNA	Gastric cancer	49	92.2	17.89	1007	0.990	NA
1194	D_15_01790_DP_CS	23	1.069	FFPE DNA	Gastric cancer	48	92.2	15.61	937.7	0.989	22.2
1195	D_15_01796_DP_CS	28	1.197	FFPE DNA	Gastric cancer	51	91.1	27.79	795.1	0.991	32.6
1196	D_15_01803_DT_CS	60	1.586	Tissue DNA	Gastric cancer	45	92.3	17.32	1053.4	0.987	63.2
1197	D_15_01804_DT_CS	71	1.642	Tissue DNA	Gastric cancer	45	92.1	13.72	1090.8	0.988	50.7
1198	D_15_01805_DT_CS	99	1.825	Tissue DNA	Gastric cancer	44.5	92.3	15.26	1229.2	0.988	94.1
1199	D_15_01806_DT_CS	105	1.496	Tissue DNA	Gastric cancer	45	92	13.65	1192.2	0.988	43.8
1200	D_15_01807_DT_CS	118	1.638	Tissue DNA	Gastric cancer	45	92.2	29.81	749.3	0.984	40.7
1201	D_15_01808_DT_CS	95	1.830	Tissue DNA	Gastric cancer	45	92.2	17.74	1297.2	0.988	75.0
1202	D_15_01809_DT_CS	76	1.468	Tissue DNA	Gastric cancer	45	92	14.97	1202.8	0.988	NA
1203	D_15_01810_DT_CS	131	1.634	Tissue DNA	Gastric cancer	45	91.6	15.53	1100.3	0.988	32.6
1204	D_15_01811_DT_CS	147	1.484	Tissue DNA	Gastric cancer	44.5	92	11.48	1043.7	0.985	38.7
1205	D_15_01812_DT_CS	139	1.659	Tissue DNA	Gastric cancer	45	91.8	12.9	922.5	0.987	66.7
1206	D_15_01813_DT_CS	63	1.799	Tissue DNA	Gastric cancer	45	92.3	16.27	948.2	0.986	50.0

1207	D_15_01814_DT_CS	51	1.524	Tissue DNA	Gastric cancer	45	92.6	55.79	451.3	0.986	32.6
1208	D_15_01815_DT_CS	72	1.468	Tissue DNA	Gastric cancer	45	92.6	13.3	986.2	0.985	27.3
1209	D_15_01816_DT_CS	129	1.550	Tissue DNA	Gastric cancer	45	92.2	18.59	937.5	0.985	85.1
1210	D_15_01817_DT_CS	148	1.697	Tissue DNA	Gastric cancer	45	93.2	49.53	595.1	0.986	50.0
1211	D_15_01818_DT_CS	61	1.535	Tissue DNA	Gastric cancer	45	92.2	49.29	588.6	0.988	27.3
1212	D_15_01819_DT_CS	91	1.598	Tissue DNA	Gastric cancer	45	92.1	56.26	451.7	0.986	85.7
1213	D_15_01916_DT_CS	77	1.356	Tissue DNA	Gastric cancer	44	93	25.16	821.1	0.975	30.7
1214	D_15_01917_DT_CS	33	0.993	Tissue DNA	Gastric cancer	44.5	92	17.5	993.7	0.982	20.2
1215	D_15_01931_DP_CS	108	1.557	FFPE DNA	Gastric cancer	49	92	19.07	1062.9	0.989	50.0
1216	D_15_01954_DT_CS	117	1.357	Tissue DNA	Gastric cancer	45.5	92.8	8.42	1021	0.982	27.3
1217	D_15_01955_DT_CS	14	1.376	Tissue DNA	Gastric cancer	45.5	93.4	25.39	798.7	0.985	24.4
1218	D_15_01961_DT_CS	53	1.144	Tissue DNA	Gastric cancer	46	93.2	21.13	787.9	0.986	85.7
1219	D_15_01976_DT_CS	10	1.418	Tissue DNA	Gastric cancer	45	90.4	18.92	898.9	0.986	22.2
1220	D_15_01977_DT_CS	12	1.234	Tissue DNA	Gastric cancer	45	89.5	20.01	971.7	0.985	NA
1221	D_15_01978_DT_CS	118	1.424	Tissue DNA	Gastric cancer	46	93	29.84	808.5	0.974	63.2
1222	D_15_01979_DT_CS	10	1.280	Tissue DNA	Gastric cancer	45	91.1	21.1	1017.3	0.986	NA
1223	D_15_01983_DT_CS	99	1.493	Tissue DNA	Gastric cancer	46	93.1	26.18	792.7	0.986	32.6
1224	D_15_02009_DP_CS	14	1.171	FFPE DNA	Gastric cancer	49	93.6	26.73	754.3	0.989	22.2
1225	D_15_02016_DP_CS	122	1.265	FFPE DNA	Gastric cancer	50	91	11.04	903.1	0.990	70.3
1226	D_15_02098_DP_CS	43	1.126	FFPE DNA	Gastric cancer	51	90.6	12	924	0.989	85.7
1227	D_15_02129_DP_CS	27	1.009	FFPE DNA	Gastric cancer	50	92.7	40.93	578.3	0.988	22.2
1228	D_15_02136_DP_CS	57	1.114	FFPE DNA	Gastric cancer	49	92.2	43.57	606.7	0.990	43.9
1229	D_15_02138_DP_CS	218	1.608	FFPE DNA	Gastric cancer	50.5	92	29.48	794.2	0.989	70.3
1230	D_15_02263_DP_CS	34	1.432	FFPE DNA	Gastric cancer	50	91.9	43.64	557.9	0.990	NA
1231	D_15_02264_DP_CS	109	0.967	FFPE DNA	Gastric cancer	50	92	59.64	360.5	0.987	85.7
1232	D_15_02366_DT_CS	168	1.586	Tissue DNA	Gastric cancer	46.5	92.7	32.52	752.4	0.987	60.6
1233	D_15_02370_DT_CS	51	1.264	Tissue DNA	Gastric cancer	46	92.5	30.88	790.5	0.987	85.7
1234	D_15_02371_DT_CS	206	1.634	Tissue DNA	Gastric cancer	46	92	32.66	643.1	0.983	94.1
1235	D_15_02397_DP_CS	88	1.332	FFPE DNA	Gastric cancer	50	90	36.9	643.6	0.989	43.9
1236	D_15_02398_DP_CS	118	1.561	FFPE DNA	Gastric cancer	52	90	24.68	735.2	0.978	100.0
1237	D_15_02407_DT_CS	9	0.849	Tissue DNA	Gastric cancer	45.5	90.7	39.87	579.4	0.985	32.6
1238	D_15_02409_DT_CS	97	1.815	Tissue DNA	Gastric cancer	46	91.2	36.43	573.4	0.983	70.1
1239	D_15_02592_DP_CS	25	1.064	FFPE DNA	Gastric cancer	48	93.1	19.74	853.2	0.986	27.3
1240	D_15_02593_DP_CS	33	1.151	FFPE DNA	Gastric cancer	49	92	28.74	722.2	0.987	43.9
1241	D_15_02596_DP_CS	105	1.226	FFPE DNA	Gastric cancer	50	89.5	31.22	743.4	0.990	63.2
1242	D_15_02603_DP_CS	14	1.197	FFPE DNA	Gastric cancer	47.5	92.3	11.16	1114.4	0.986	27.3
1243	D_15_02604_DP_CS	17	1.006	FFPE DNA	Gastric cancer	48.5	92.2	9.86	1104.4	0.988	27.3
1244	D_15_02896_DT_CS	68	1.009	Tissue DNA	Gastric cancer	46	91.8	29.28	719.6	0.986	43.9
1245	D_15_04030_DT_CS	15	1.171	Tissue DNA	Gastric cancer	46	94.8	34.35	549.3	0.983	22.2
1246	D_15_04033_DT_CS	11	0.860	Tissue DNA	Gastric cancer	44.5	95.2	34.12	544.8	0.979	24.4
1247	D_15_04037_DP_CS	43	1.273	FFPE DNA	Gastric cancer	49	91.1	34.4	730.4	0.991	NA
1248	D_15_04038_DP_CS	122	1.561	FFPE DNA	Gastric cancer	50	92.4	37.68	562.5	0.984	100.0
1249	D_15_04039_DP_CS	10	0.802	FFPE DNA	Gastric cancer	48.5	92.8	40.66	560.5	0.990	NA
1250	D_15_04040_DP_CS	14	1.091	FFPE DNA	Gastric cancer	50	94	46.01	439.4	0.986	27.3
1251	D_15_04041_DP_CS	27	1.501	FFPE DNA	Gastric cancer	49	91.2	26.21	800.7	0.990	27.3
1252	D_15_04042_DP_CS	52	1.454	FFPE DNA	Gastric cancer	50	93.8	45.86	414.8	0.985	22.2
1253	D_15_04043_DP_CS	51	1.529	FFPE DNA	Gastric cancer	50	91.2	36.96	723	0.990	38.1
1254	D_15_04093_DT_CS	129	1.663	Tissue DNA	Gastric cancer	46	94.7	29.39	640.4	0.981	79.9
1255	D_15_04130_DT_CS	127	1.518	Tissue DNA	Gastric cancer	46	94.8	30.31	624.5	0.984	70.1
1256	D_15_04131_DT_CS	14	1.332	Tissue DNA	Gastric cancer	46	94.9	29.74	680.7	0.985	32.6
1257	D_15_04145_DT_CS	107	1.601	Tissue DNA	Gastric cancer	46	91.5	40.43	676.8	0.987	55.1
1258	D_15_04147_DT_CS	84	1.261	Tissue DNA	Gastric cancer	46	91.7	33.84	663.7	0.986	85.7
1259	D_15_04150_DP_CS	0	0.000	FFPE DNA	Gastric cancer	50	92.6	40.23	604.4	0.989	27.3
1260	D_15_04151_DP_CS	52	1.768	FFPE DNA	Gastric cancer	50	90.8	29.91	530.1	0.984	27.3
1261	D_15_04223_DT_CS	130	1.502	Tissue DNA	Gastric cancer	45	92.2	28.77	610	0.980	48.0
1262	D_15_04224_DT_CS	65	1.335	Tissue DNA	Gastric cancer	45.5	91.5	31.05	616.1	0.982	60.2
1263	D_15_04225_DT_CS	106	1.455	Tissue DNA	Gastric cancer	46	92.3	33.14	596	0.984	77.8
1264	D_15_04256_DT_CS	159	1.665	Tissue DNA	Gastric cancer	45	92.2	38.38	604.5	0.984	75.0
1265	D_15_04469_DT_CS	112	1.746	Tissue DNA	Gastric cancer	NA	NA	35.71	805.7	0.986	76.5
1266	D_15_04475_DP_CS	41	1.298	FFPE DNA	Gastric cancer	50	92.8	52.53	374.2	0.987	23.4
1267	D_15_04476_DP_CS	26	1.139	FFPE DNA	Gastric cancer	47	92.8	23.73	851	0.988	NA
1268	D_15_04477_DP_CS	13	1.458	FFPE DNA	Gastric cancer	49	92.5	18.81	924.1	0.990	32.6
1269	D_15_04483_DP_CS	14	1.061	FFPE DNA	Gastric cancer	49	92.8	22.73	910.3	0.991	NA
1270	D_15_04487_DP_CS	70	1.039	FFPE DNA	Gastric cancer	51	91.5	25.06	806.5	0.990	43.9

1271	D_15_04505_DP_CS	20	1.350	FFPE DNA	Gastric cancer	NA	NA	19	869.7	0.989	24.9
1272	D_15_04527_DT_CS	11	0.655	Tissue DNA	Gastric cancer	45.5	92.4	17.57	948.2	0.986	27.3
1273	D_15_04528_DT_CS	167	1.554	Tissue DNA	Gastric cancer	45	92.2	19.04	1013.6	0.985	50.5
1274	D_15_04795_DP_CS	29	1.086	FFPE DNA	Gastric cancer	48	92	17.89	966.3	0.988	38.1
1275	D_15_04796_DP_CS	17	1.203	FFPE DNA	Gastric cancer	49	91.2	18.27	817.3	0.989	32.6
1276	D_15_04798_DP_CS	40	1.094	FFPE DNA	Gastric cancer	50	90.8	17.7	800.9	0.988	32.6
1277	D_15_04799_DP_CS	151	1.528	FFPE DNA	Gastric cancer	50	90.9	22.23	742.4	0.987	85.7
1278	D_15_04800_DP_CS	15	1.338	FFPE DNA	Gastric cancer	50	90.6	20.35	761.8	0.989	NA
1279	D_15_04809_TS_CS	27	1.288	Frozen Tissue	Gastric cancer	46	91.6	12.99	1121.7	0.985	21.4
1280	D_15_04810_TS_CS	139	1.676	Frozen Tissue	Gastric cancer	46	91.4	14.05	1090.2	0.987	70.3
1281	D_15_04830_DP_CS	168	1.840	FFPE DNA	Gastric cancer	48	90.3	24.37	897.9	0.989	100.0
1282	D_15_04831_DP_CS	8	1.074	FFPE DNA	Gastric cancer	50	89.5	18.09	911.5	0.990	NA
1283	D_15_04837_DT_CS	38	0.884	Tissue DNA	Gastric cancer	46	92.7	12.14	1169.5	0.987	21.1
1284	D_15_04847_DP_CS	51	1.216	FFPE DNA	Gastric cancer	51	90	21.69	1033.3	0.992	24.6
1285	D_15_04848_DP_CS	162	1.570	FFPE DNA	Gastric cancer	49.5	91.1	12.14	1096.2	0.988	44.0
1286	D_15_04849_DP_CS	45	1.181	FFPE DNA	Gastric cancer	49	91.1	14.04	1066.9	0.988	32.6
1287	D_15_04852_DP_CS	56	1.248	FFPE DNA	Gastric cancer	47.5	91.8	13.23	1267.7	0.990	50.0
1288	D_15_04853_DP_CS	23	1.182	FFPE DNA	Gastric cancer	47.5	90.5	14.26	1132.9	0.991	20.2
1289	D_15_04888_DT_CS	78	1.166	Tissue DNA	Gastric cancer	45	93.4	16.51	1108	0.987	43.9
1290	D_15_04930_DT_CS	8	0.736	Tissue DNA	Gastric cancer	45.5	93.3	16.29	1068.8	0.987	22.2
1291	D_15_04931_DP_CS	88	0.472	FFPE DNA	Gastric cancer	49	92.8	18.35	1026.9	0.989	84.0
1292	D_15_04932_DP_CS	7	1.277	FFPE DNA	Gastric cancer	50	92.1	14.39	1035.7	0.990	22.2
1293	D_15_04933_DP_CS	19	1.129	FFPE DNA	Gastric cancer	51	91.1	28.61	745.2	0.989	NA
1294	D_15_04934_DP_CS	39	1.103	FFPE DNA	Gastric cancer	49	92.1	18.07	931.3	0.990	27.3
1295	D_15_04935_DT_CS	123	1.370	Tissue DNA	Gastric cancer	46	92.7	11.16	944	0.984	32.6
1296	D_15_04936_DT_CS	63	0.317	Tissue DNA	Gastric cancer	46	92.7	14.18	1156.1	0.988	22.2
1297	D_15_04937_DT_CS	14	1.061	Tissue DNA	Gastric cancer	45.5	92.2	13.05	1141.8	0.986	32.6
1298	D_15_05026_DP_CS	17	0.846	FFPE DNA	Gastric cancer	NA	NA	44.56	574.9	0.990	20.2
1299	D_15_05057_DP_CS	18	0.778	FFPE DNA	Gastric cancer	NA	NA	17.03	1181	0.992	38.1
1300	D_15_05076_TS_CS	48	1.309	Fresh Tissue	Gastric cancer	NA	NA	14.15	1121.5	0.987	28.6
1301	15_00061_DP_CS	104	1.154	FFPE DNA	Pancreatic cancer	48.5	92.7	14.53	1148.4	0.991	100.0
1302	15_00063_DP_CS	88	1.572	FFPE DNA	Pancreatic cancer	48.5	93.1	19.35	1047.5	0.991	100.0
1303	15_00284_DP_CS	16	1.251	FFPE DNA	Pancreatic cancer	50	92.8	23.98	771.6	0.991	32.6
1304	15_00404_DP_CS	7	0.410	FFPE DNA	Pancreatic cancer	48	93.8	23.2	808.5	0.989	27.3
1305	15_00406_DP_CS	28	1.544	FFPE DNA	Pancreatic cancer	50	92	29.39	764.3	0.991	100.0
1306	15_00407_DP_CS	19	1.490	FFPE DNA	Pancreatic cancer	49	92.4	24.55	790.4	0.991	100.0
1307	15_00429_DP_CS	33	1.195	FFPE DNA	Pancreatic cancer	50	92.3	27.37	863.3	0.991	32.6
1308	D_15_00806_DP_CS	27	0.923	FFPE DNA	Pancreatic cancer	48.5	92	13.16	958.9	0.990	32.6
1309	D_15_00926_TS_CS	124	1.606	Frozen Tissue	Pancreatic cancer	45.5	91.4	19.1	1120.6	0.988	100.0
1310	D_15_00927_TS_CS	32	0.986	Frozen Tissue	Pancreatic cancer	45.5	92.6	14.92	881.4	0.984	27.6
1311	D_15_00928_TS_CS	49	1.314	Frozen Tissue	Pancreatic cancer	46	92.5	13.48	907.3	0.985	100.0
1312	D_15_01005_DP_CS	56	1.324	FFPE DNA	Pancreatic cancer	50	91.3	27.43	939.2	0.991	22.2
1313	D_15_01009_DP_CS	43	1.576	FFPE DNA	Pancreatic cancer	49	91.2	38.78	741.2	0.991	43.9
1314	D_15_01173_DP_CS	28	1.515	FFPE DNA	Pancreatic cancer	49	91.4	22.25	912.4	0.991	NA
1315	D_15_01235_DP_CS	52	1.335	FFPE DNA	Pancreatic cancer	53	90.2	32.96	760.7	0.989	32.6
1316	D_15_01387_TS_CS	71	1.708	Frozen Tissue	Pancreatic cancer	45	93	13.54	968	0.984	71.8
1317	D_15_01388_TS_CS	58	1.272	Frozen Tissue	Pancreatic cancer	45	92.8	13.13	966.8	0.984	34.3
1318	D_15_01389_TS_CS	49	1.463	Frozen Tissue	Pancreatic cancer	45	93.2	11.32	786.5	0.982	NA
1319	D_15_01390_TS_CS	22	1.244	Frozen Tissue	Pancreatic cancer	45.5	93	10.4	944.4	0.984	24.6
1320	D_15_01391_TS_CS	17	1.236	Frozen Tissue	Pancreatic cancer	45	92.9	13.52	936.4	0.984	NA
1321	D_15_01393_TS_CS	57	1.263	Frozen Tissue	Pancreatic cancer	45	92.8	13.14	908.7	0.983	27.4
1322	D_15_01394_TS_CS	55	1.163	Frozen Tissue	Pancreatic cancer	45	93.3	10.81	980.8	0.982	26.4
1323	D_15_01396_TS_CS	15	1.252	Frozen Tissue	Pancreatic cancer	44.5	92.3	11.82	1026.6	0.980	32.6
1324	D_15_01443_DP_CS	55	1.719	FFPE DNA	Pancreatic cancer	49	90.5	21.49	1058.8	0.992	38.1
1325	D_15_01447_DP_CS	28	1.494	FFPE DNA	Pancreatic cancer	48.5	92.5	18.06	922.6	0.991	32.6
1326	D_15_01449_DP_CS	28	1.327	FFPE DNA	Pancreatic cancer	52	90	18.56	1108.6	0.992	28.9
1327	D_15_01769_DT_CS	50	1.187	Tissue DNA	Pancreatic cancer	45.5	93	22.57	1029.8	0.987	43.8
1328	D_15_01788_DP_CS	33	0.817	FFPE DNA	Pancreatic cancer	48.5	92.2	17.77	1027.2	0.989	35.6
1329	D_15_01798_DT_CS	131	1.510	Tissue DNA	Pancreatic cancer	45	93.1	15.97	881.6	0.984	63.0
1330	D_15_01924_DP_CS	23	1.438	FFPE DNA	Pancreatic cancer	49	92.1	12.61	1079.2	0.989	77.8
1331	D_15_01928_DP_CS	37	0.878	FFPE DNA	Pancreatic cancer	48.5	91.9	16.58	889.4	0.988	43.9
1332	D_15_01929_DP_CS	18	1.040	FFPE DNA	Pancreatic cancer	49	91.4	19.55	890.4	0.990	NA
1333	D_15_01997_DT_CS	64	1.050	Tissue DNA	Pancreatic cancer	45.5	94.9	24.68	705.3	0.983	32.6
1334	D_15_02097_DP_CS	25	1.141	FFPE DNA	Pancreatic cancer	49.5	91.2	23.79	876	0.991	27.3

1335	D_15_02369_DT_CS	37	1.676	Tissue DNA	Pancreatic cancer	46	92.3	36.75	704.7	0.987	27.3
1336	D_15_04152_FN_CS	12	1.265	Fine Niddle Biopsy	Pancreatic cancer	45	93.1	21.65	935.4	0.986	22.2
1337	D_15_04153_FN_CS	60	1.223	Fine Niddle Biopsy	Pancreatic cancer	46	91.8	20.91	931.8	0.986	43.8
1338	D_15_04154_FN_CS	15	1.252	Fine Niddle Biopsy	Pancreatic cancer	46	91.5	18.08	956.8	0.987	27.3
1339	D_15_04155_FN_CS	30	1.569	Fine Niddle Biopsy	Pancreatic cancer	46	91.8	20.15	985.8	0.988	38.1
1340	D_15_04156_FN_CS	9	1.273	Fine Niddle Biopsy	Pancreatic cancer	46	92.2	19.41	929	0.987	43.9
1341	D_15_04157_FN_CS	18	1.355	Fine Niddle Biopsy	Pancreatic cancer	46.5	91.9	18.43	965	0.987	32.6
1342	D_15_04158_FN_CS	49	1.356	Fine Niddle Biopsy	Pancreatic cancer	46	91.5	20.93	1152.8	0.988	38.7
1343	D_15_04159_FN_CS	83	1.231	Fine Niddle Biopsy	Pancreatic cancer	46	91.6	17.62	1028.3	0.987	34.3
1344	D_15_04160_FN_CS	78	1.120	Fine Niddle Biopsy	Pancreatic cancer	47	91.5	16.83	1090	0.988	36.1
1345	D_15_04161_FN_CS	79	1.179	Fine Niddle Biopsy	Pancreatic cancer	47	92.2	19.06	1012.8	0.988	94.1
1346	D_15_04162_FN_CS	132	1.632	Fine Niddle Biopsy	Pancreatic cancer	46	92	19.87	935.5	0.985	43.0
1347	D_15_04163_FN_CS	183	1.796	Fine Niddle Biopsy	Pancreatic cancer	46.5	91.7	19.47	953.1	0.987	54.7
1348	D_15_04164_FN_CS	13	1.231	Fine Niddle Biopsy	Pancreatic cancer	46	91.7	18.59	847.8	0.986	43.9
1349	D_15_04165_FN_CS	93	1.174	Fine Niddle Biopsy	Pancreatic cancer	46	91.2	18.93	902.7	0.984	38.4
1350	D_15_04166_FN_CS	74	1.269	Fine Niddle Biopsy	Pancreatic cancer	46.5	90.5	17.12	900.6	0.986	85.7
1351	D_15_04167_FN_CS	20	0.926	Fine Niddle Biopsy	Pancreatic cancer	46	91.6	15.3	988.4	0.986	NA
1352	D_15_04168_FN_CS	64	1.444	Fine Niddle Biopsy	Pancreatic cancer	46	91.4	18.53	1032.4	0.987	31.7

Table S2. List of target genes (n=50, 100, 200, 300 and 381)

Num	50 Genes	100 Genes	200 Genes	300 Genes	381 Genes
1	ABL1	MTOR	MTOR	MTOR	TNFRSF14
2	AKT1	ARID1A	ARID1A	ARID1A	MTOR
3	ALK	MPL	MPL	MPL	SPEN
4	APC	PTCH2	PTCH2	PTCH2	CDC42
5	ATM	JAK1	JAK1	JAK1	ARID1A
6	BRAF	NRAS	NRAS	NRAS	MYCL1
7	CDH1	NTRK1	NTRK1	NTRK1	MPL
8	CDKN2A	DDR2	DDR2	DDR2	PTCH2
9	CSF1R	AKT3	AKT3	AKT3	MUTYH
10	CTNNB1	ALK	ALK	ALK	RAD54L
11	DDR2	IDH1	IDH1	IDH1	CDKN2C
12	EGFR	ERBB4	ERBB4	ERBB4	JUN
13	ERBB2	VHL	VHL	VHL	JAK1
14	ERBB4	MLH1	MLH1	MLH1	FUBP1
15	EZH2	CTNNB1	CTNNB1	CTNNB1	NRAS
16	FBXW7	PIK3CA	PIK3CA	PIK3CA	FAM46C
17	FGFR1	FGFR3	FGFR3	FGFR3	NOTCH2
18	FGFR2	PDGFRA	PDGFRA	PDGFRA	MCL1
19	FGFR3	KIT	KIT	KIT	NTRK1
20	FLT3	KDR	KDR	KDR	DDR2
21	GNA11	FBXW7	FBXW7	FBXW7	ABL2
22	GNAQ	PIK3R1	PIK3R1	PIK3R1	CDC73
23	HNF1A	APC	APC	APC	MDM4
24	HRAS	CSF1R	CSF1R	CSF1R	IKBKE
25	IDH1	PDGFRB	PDGFRB	PDGFRB	PARP1
26	IDH2	ITK	ITK	ITK	H3F3A
27	JAK2	NPM1	NPM1	NPM1	AKT3
28	JAK3	ROS1	ROS1	ROS1	MYCN
29	KDR	ARID1B	ARID1B	ARID1B	DNMT3A
30	KIT	EGFR	EGFR	EGFR	ALK
31	KRAS	CDK6	CDK6	CDK6	SFRS7
32	MET	EPHB4	EPHB4	EPHB4	MSH2
33	MLH1	MET	MET	MET	MSH6
34	MPL	SMO	SMO	SMO	FANCL
35	NOTCH1	BRAF	BRAF	BRAF	REL
36	NRAS	EZH2	EZH2	EZH2	XPO1
37	PDGFRA	FGFR1	FGFR1	FGFR1	LRP1B
38	PIK3CA	JAK2	JAK2	JAK2	PDK1
39	PTEN	CDKN2A	CDKN2A	CDKN2A	NFE2L2
40	PTPN11	GNAQ	GNAQ	GNAQ	STAT4
41	RB1	SYK	SYK	SYK	SF3B1
42	RET	PTCH1	PTCH1	PTCH1	CASP8
43	SMAD4	ABL1	ABL1	ABL1	IDH1
44	SMARCB1	NOTCH1	NOTCH1	NOTCH1	ERBB4
45	SMO	RET	RET	RET	BARD1
46	SRC	PTEN	PTEN	PTEN	CRBN
47	STK11	FGFR2	FGFR2	FGFR2	FANCD2
48	TP53	HRAS	HRAS	HRAS	VHL
49	VHL	ATM	ATM	ATM	RAF1
50	ROS1	KRAS	KRAS	KRAS	TGFBR2
51		ARID2	ARID2	ARID2	MLH1
52		ERBB3	ERBB3	ERBB3	MYD88
53		CDK4	CDK4	CDK4	CTNNB1
54		MDM2	MDM2	MDM2	SETD2
55		PTPN11	PTPN11	PTPN11	PARP3
56		HNF1A	HNF1A	HNF1A	BAP1
57		FLT3	FLT3	FLT3	PBRM1
58		BRCA2	BRCA2	BRCA2	MITF
59		RB1	RB1	RB1	ROBO2
60		AKT1	AKT1	AKT1	ROBO1

61	IDH2	IDH2	IDH2	EPHA3
62	IGF1R	IGF1R	IGF1R	EPHA6
63	CDH1	CDH1	CDH1	GSK3B
64	TP53	TP53	TP53	GATA2
65	AURKB	AURKB	AURKB	EPHB1
66	NF1	NF1	NF1	FOXL2
67	ERBB2	ERBB2	ERBB2	ATR
68	BRCA1	BRCA1	BRCA1	TIPARP
69	SMAD4	SMAD4	SMAD4	PIK3CA
70	BCL2	BCL2	BCL2	SOX2
71	STK11	STK11	STK11	KLHL6
72	GNA11	GNA11	GNA11	MAP3K13
73	JAK3	JAK3	JAK3	ETV5
74	AKT2	AKT2	AKT2	BCL6
75	SRC	SRC	SRC	FGF12
76	TOP1	TOP1	TOP1	FGFR3
77	AURKA	AURKA	AURKA	SLIT2
78	GNAS	GNAS	GNAS	PDGFRA
79	SMARCB1	SMARCB1	SMARCB1	KIT
80	ATRX	ATRX	ATRX	KDR
81	TNFRSF14	TNFRSF14	TNFRSF14	EPHA5
82	SPEN	SPEN	SPEN	TET2
83	CDC42	CDC42	CDC42	INPP4B
84	MYCL1	MYCL1	MYCL1	FBXW7
85	MUTYH	MUTYH	MUTYH	TERT
86	RAD54L	RAD54L	RAD54L	IL7R
87	CDKN2C	CDKN2C	CDKN2C	SKP2
88	JUN	JUN	JUN	RICTOR
89	FUBP1	FUBP1	FUBP1	FGF10
90	FAM46C	FAM46C	FAM46C	MAP3K1
91	NOTCH2	NOTCH2	NOTCH2	PIK3R1
92	MCL1	MCL1	MCL1	CHD1
93	ABL2	ABL2	ABL2	APC
94	CDC73	CDC73	CDC73	RAD50
95	MDM4	MDM4	MDM4	CTNNA1
96	IKBKE	IKBKE	IKBKE	CSF1R
97	PARP1	PARP1	PARP1	PDGFRB
98	H3F3A	H3F3A	H3F3A	ITK
99	MYCN	MYCN	MYCN	DOCK2
100	DNMT3A	DNMT3A	DNMT3A	NPM1
101		SFRS7	SFRS7	FGFR4
102		MSH2	MSH2	NSD1
103		MSH6	MSH6	MAML1
104		FANCL	FANCL	FLT4
105		REL	REL	IRF4
106		XPO1	XPO1	HIST1H3B
107		LRP1B	LRP1B	HLA-A
108		PDK1	PDK1	NOTCH4
109		NFE2L2	NFE2L2	BRD2
110		STAT4	STAT4	DAXX
111		SF3B1	SF3B1	FANCE
112		CASP8	CASP8	CCND3
113		BARD1	BARD1	PKHD1
114		CRBN	CRBN	PNRC1
115		FANCD2	FANCD2	EPHA7
116		RAF1	RAF1	PRDM1
117		TGFBR2	TGFBR2	WISP3
118		MYD88	MYD88	ROS1
119		SETD2	SETD2	TNFAIP3
120		PARP3	PARP3	ESR1
121		BAP1	BAP1	ARID1B
122		PBRM1	PBRM1	IGF2R
123		MITF	MITF	CARD11
124		ROBO2	ROBO2	PMS2

125	ROBO1	ROBO1	RAC1
126	EPHA3	EPHA3	ETV1
127	EPHA6	EPHA6	HOXA3
128	GSK3B	GSK3B	ELMO1
129	GATA2	GATA2	INHBA
130	EPHB1	EPHB1	IKZF1
131	FOXL2	FOXL2	EGFR
132	ATR	ATR	HGF
133	TIPARP	TIPARP	SEMA3E
134	SOX2	SOX2	SEMA3A
135	KLHL6	KLHL6	CDK6
136	MAP3K13	MAP3K13	TRRAP
137	ETV5	ETV5	EPHB4
138	BCL6	BCL6	PIK3CG
139	FGF12	FGF12	MET
140	SLIT2	SLIT2	SMO
141	EPHA5	EPHA5	BRAF
142	TET2	TET2	EPHB6
143	INPP4B	INPP4B	EZH2
144	TERT	TERT	MLL3
145	IL7R	IL7R	TNKS
146	SKP2	SKP2	NKX3-1
147	RICTOR	RICTOR	ZNF703
148	FGF10	FGF10	GPR124
149	MAP3K1	MAP3K1	WHSC1L1
150	CHD1	CHD1	FGFR1
151	RAD50	RAD50	KAT6A
152	CTNNA1	CTNNA1	PRKDC
153	DOCK2	DOCK2	WWP1
154	FGFR4	FGFR4	RUNX1T1
155	NSD1	NSD1	MYC
156	MAML1	MAML1	JAK2
157	FLT4	FLT4	PTPRD
158	IRF4	IRF4	CDKN2A
159	HIST1H3B	HIST1H3B	CDKN2B
160	HLA-A	HLA-A	FANCG
161	NOTCH4	NOTCH4	PAX5
162	BRD2	BRD2	GNAQ
163	DAXX	DAXX	NTRK2
164	FANCE	FANCE	SYK
165	CCND3	CCND3	FANCC
166	PKHD1	PKHD1	PTCH1
167	PNRC1	PNRC1	KLF4
168	EPHA7	EPHA7	ABL1
169	PRDM1	PRDM1	TSC1
170	WISP3	WISP3	BRD3
171	TNFAIP3	TNFAIP3	NOTCH1
172	ESR1	ESR1	GATA3
173	IGF2R	IGF2R	RET
174	CARD11	CARD11	PTEN
175	PMS2	PMS2	TNKS2
176	RAC1	RAC1	CHUK
177	ETV1	ETV1	SUFU
178	HOXA3	HOXA3	CYP17A1
179	ELMO1	ELMO1	FGFR2
180	INHBA	INHBA	HRAS
181	IKZF1	IKZF1	LMO1
182	HGF	HGF	FANCF
183	SEMA3E	SEMA3E	WT1
184	SEMA3A	SEMA3A	MEN1
185	TRRAP	TRRAP	CCND1
186	PIK3CG	PIK3CG	FGF19
187	EPHB6	EPHB6	FGF4
188	MLL3	MLL3	FGF3

189	TNKS	TNKS	C11ORF30
190	NKX3-1	NKX3-1	FAT3
191	ZNF703	ZNF703	MRE11A
192	GPR124	GPR124	PGR
193	WHSC1L1	WHSC1L1	GUCY1A2
194	KAT6A	KAT6A	ATM
195	PRKDC	PRKDC	KMT2A
196	WWP1	WWP1	MLL
197	RUNX1T1	RUNX1T1	CBL
198	MYC	MYC	CHEK1
199	PTPRD	PTPRD	KDM5A
200	CDKN2B	CDKN2B	RAD52
201		FANCG	CCND2
202		PAX5	FGF23
203		NTRK2	FGF6
204		FANCC	CHD4
205		KLF4	ETV6
206		TSC1	LRP6
207		BRD3	CDKN1B
208		GATA3	KRAS
209		TNKS2	ARID2
210		CHUK	MLL2
211		SUFU	PRPF40B
212		CYP17A1	SMARCD1
213		LMO1	ACVR1B
214		FANCF	ERBB3
215		WT1	CDK4
216		MEN1	MDM2
217		CCND1	BTG1
218		FGF19	IGF1
219		FGF4	SH2B3
220		FGF3	PTPN11
221		C11ORF30	TBX3
222		FAT3	RAB35
223		MRE11A	HNF1A
224		PGR	PARP4
225		GUCY1A2	CDK8
226		KMT2A	CDX2
227		MLL	FLT3
228		CBL	FLT1
229		CHEK1	BRCA2
230		KDM5A	RB1
231		RAD52	DIS3
232		CCND2	FGF14
233		FGF23	IRS2
234		FGF6	CUL4A
235		CHD4	PARP2
236		ETV6	BCL2L2
237		LRP6	NFKBIA
238		CDKN1B	NKX2-1
239		MLL2	FOXA1
240		PRPF40B	FANCM
241		SMARCD1	RAD51B
242		ACVR1B	TSHR
243		BTG1	HSP90AA1
244		IGF1	XRCC3
245		SH2B3	AKT1
246		TBX3	C15ORF55
247		RAB35	RAD51
248		PARP4	LTK
249		CDK8	B2M
250		CDX2	FGF7
251		FLT1	MAP2K1
252		DIS3	SMAD3

253	FGF14	BCL2A1
254	IRS2	NTRK3
255	CUL4A	FANCI
256	PARP2	IDH2
257	BCL2L2	BLM
258	NFKBIA	CHD2
259	NKX2-1	IGF1R
260	FOXA1	AXIN1
261	FANCM	TSC2
262	RAD51B	TRAF7
263	TSHR	CREBBP
264	HSP90AA1	GRIN2A
265	XRCC3	SOCS1
266	C15ORF55	PALB2
267	RAD51	PRSS8
268	LTK	CYLD
269	B2M	NUP93
270	FGF7	CDH5
271	MAP2K1	CBFB
272	SMAD3	CTCF
273	BCL2A1	CDH1
274	NTRK3	PHLPP2
275	FANCI	FANCA
276	BLM	RPA1
277	CHD2	TP53
278	AXIN1	ALOX12B
279	TSC2	AURKB
280	TRAF7	MAP2K4
281	CREBBP	NCOR1
282	GRIN2A	GID4
283	SOCS1	NF1
284	PALB2	RAD51D
285	PRSS8	CDK12
286	CYLD	ERBB2
287	NUP93	RARA
288	CDH5	STAT3
289	CBFB	BRCA1
290	CTCF	ETV4
291	PHLPP2	SPOP
292	FANCA	SRSF1
293	RPA1	RNF43
294	ALOX12B	RAD51C
295	MAP2K4	BRIP1
296	NCOR1	CD79B
297	GID4	GNA13
298	RAD51D	PRKAR1A
299	CDK12	SOX9
300	RARA	SRSF2
301		RPTOR
302		APCDD1
303		CDH2
304		PIK3C3
305		SETBP1
306		SMAD2
307		SMAD4
308		CDH20
309		BCL2
310		STK11
311		DOT1L
312		GNA11
313		MAP2K2
314		INSR
315		DNMT1
316		KEAP1

317	SMARCA4
318	NOTCH3
319	BRD4
320	JAK3
321	PIK3R2
322	MEF2B
323	CCNE1
324	CEBPA
325	AKT2
326	AXL
327	CD79A
328	CIC
329	ERCC2
330	PPP2R1A
331	U2AF2
332	PAK7
333	BCL2L1
334	ASXL1
335	SRC
336	TOP1
337	PLCG1
338	EYA2
339	NCOA3
340	ZNF217
341	AURKA
342	GNAS
343	ARFRP1
344	BACH1
345	RUNX1
346	ERG
347	TMPRSS2
348	U2AF1
349	CRKL
350	BCR
351	SMARCB1
352	CHEK2
353	XBP1
354	EWSR1
355	NF2
356	SF3A1
357	RAC2
358	SOX10
359	APOBEC3A
360	APOBEC3B
361	EP300
362	CRLF2
363	ZRSR2
364	BCOR
365	USP9X
366	KDM6A
367	ARAF
368	GATA1
369	KDM5C
370	FAM123B
371	AR
372	MED12
373	ATRX
374	TBX22
375	BTK
376	PAK3
377	CUL4B
378	STAG2
379	SMARCA1
380	BCORL1

Table S3. List of 21 cancer types in The Cancer Genome Atlas (TCGA)

Num	Cohort	Disease Name	# Sample
1	UCS	Uterine Carcinosarcom	57
2	READ	Rectum adenocarcinoma	156
3	COAD	Colon adenocarcinoma	494
4	OV	Ovarian serous cystadenocarcinoma	438
5	LIHC	Liver hepatocellular carcinoma	375
6	ACC	Adrenocortical carcinoma	92
7	LUSC	Lung squamous cell carcinoma	491
8	SKCM	Skin Cutaneous Melanoma	470
9	BLCA	Bladder urothelial carcinoma	412
10	GBM	Glioblastoma multiforme	393
11	UCEC	Uterine Corpus Endometrial Carcinoma	544
12	KIRP	Kidney renal papillary cell carcinoma	288
13	CESC	Cervical and endocervical cancers	305
14	LGG	Brain Lower Grade Glioma	515
15	BRCA	Breast invasive carcinoma	1081
16	HNSC	Head and Neck squamous cell carcinoma	510
17	LUAD	Lung adenocarcinoma	517
18	KICH	Kidney Chromophobe	66
19	PRAD	Prostate adenocarcinoma	498
20	KIRC	Kidney renal clear cell carcinoma	384
21	THCA	Thyroid carcinoma	492

Table S4. Correlation between ITH and Purity (or Mutation Number)

ID	Median			Correlation	
	ITH	Purity	#Mutations	ITH&Purity	ITH&#Mutation
ACC	1.710	92.5	29	0.287	0.365
BLCA	1.666	74.6	155	0.551	0.031
BRCA	1.555	74.7	83	0.545	-0.128
CESC	1.623	78.1	94	0.444	0.175
COAD	1.733	78.3	260.5	0.471	-0.003
GBM	1.636	83.6	87.5	0.408	0.068
HNSC	1.472	68.8	52	0.633	0.266
KICH	1.440	88.8	118	0.081	-0.354
KIRC	1.410	68.4	218	0.411	0.200
KIRP	1.627	82.5	59	0.482	0.038
LGG	1.593	91.0	73	0.257	-0.057
LIHC	1.705	79.5	27	0.436	0.235
LUAD	1.456	63.9	73.5	0.647	-0.330
LUSC	1.672	68.6	29	0.725	0.250
OV	1.736	87.0	46	0.350	-0.098
PRAD	1.410	89.7	28	0.201	0.315
READ	1.801	78.9	81	0.447	0.101
SKCM	1.660	76.5	184	0.645	0.270
THCA	1.264	87.4	13	0.264	0.300
UCEC	1.631	84.5	43	0.426	0.142
UCS	2.007	83.6	61	0.380	0.082

Table S5. The list of heterogeneity index measured in CRC.

patients	TH index	SNV num	KRAS	APC	TP53	Age	CEA	Cell Type	Lymph Invasion	Vas Invasion	Per Invasion	Tumor Bud	Tumor Purity	event	time
D_15_02483_DP_CS	0.684	9	0	0	0	57	0	0	0	0	0	0	70	0	35.1
D_15_02549_DP_CS	1.595	29	0	0	1	53	0	0	0	0	0	1	70	0	24.4
D_15_02440_DP_CS	0.802	10	0	0	0	60	0	1	0	0	0	0	40	1	12.0
D_15_02466_DP_CS	1.067	52	0	0	0	61	0	0	0	0	1	0	60	0	36.0
D_15_02525_DP_CS	1.288	61	1	1	1	46	0	0	0	0	0	0	75	0	30.3
D_15_02493_DP_CS	1.206	19	0	0	0	57	0	0	0	0	0	0	60	0	31.6
D_15_02296_DP_CS	0.943	19	1	0	0	39	0	0	1	1	0	1	50	1	42.6
D_15_02510_DP_CS	0.908	35	0	0	0	68	0	1	0	0	0	0	70	0	32.8
D_15_02335_DP_CS	1.353	61	0	1	1	32	1	1	1	1	1	1	80	1	5.8
D_15_02275_DP_CS	0.959	42	0	0	1	62	0	0	0	0	0	0	60	1	15.1
D_15_02519_DP_CS	1.277	38	1	1	0	68	0	1	0	0	0	1	70	0	33.3
D_15_02541_DP_CS	1.409	70	1	1	1	51	1	0	1	1	0	1	80	0	29.0
D_15_02348_DP_CS	1.489	37	1	1	0	52	1	1	1	0	0	1	70	1	4.8
D_15_02337_DP_CS	0.824	12	0	0	0	31	2	1	1	0	1	1	35	0	55.2
D_15_02518_DP_CS	1.429	81	0	1	0	52	0	0	0	0	0	0	70	0	29.3
D_15_02548_DP_CS	1.360	22	1	1	0	56	0	0	0	0	1	1	80	0	29.2
D_15_02516_DP_CS	1.256	53	0	1	0	65	0	0	1	1	0	0	70	0	31.5
D_15_02308_DP_CS	1.251	45	0	0	1	58	2	0	0	0	0	0	70	1	49.5
D_15_02503_DP_CS	1.652	75	0	0	1	63	0	0	1	1	1	1	75	1	23.1
D_15_02553_DP_CS	1.624	71	0	0	0	73	1	1	1	1	0	1	70	1	0.6
D_15_02491_DP_CS	1.282	30	1	0	0	63	0	0	0	1	1	1	65	0	15.9
D_15_02288_DP_CS	1.189	36	1	0	1	47	0	0	0	0	0	0	75	1	15.5
D_15_02158_DP_CS	1.134	60	0	0	1	58	0	0	0	0	0	2	70	1	39.2
D_15_02557_DP_CS	1.112	75	1	1	0	25	0	1	0	0	0	0	80	0	24.8
D_15_02159_DP_CS	1.614	79	0	1	0	70	0	0	2	2	2	2	80	1	4.2
D_15_02324_DP_CS	1.787	89	1	0	1	65	0	0	0	0	0	1	70	1	13.1
D_15_02517_DP_CS	1.158	144	1	0	0	51	0	1	0	0	0	1	60	0	33.1
D_15_02346_DP_CS	1.134	46	1	0	0	38	1	0	1	1	0	1	75	1	9.6
D_15_02550_DP_CS	0.932	46	0	0	1	56	0	0	1	0	1	0	70	0	29.5
D_15_02479_DP_CS	0.666	37	0	0	0	48	0	0	0	0	0	0	70	0	35.9
D_15_02449_DP_CS	1.436	56	0	0	0	69	0	0	0	0	0	1	80	1	31.1
D_15_02364_DP_CS	0.677	17	1	1	1	52	0	0	0	0	1	0	50	1	17.3
D_15_02509_DP_CS	1.112	59	0	0	1	70	0	0	1	0	0	1	70	1	30.8
D_15_02330_DP_CS	0.956	7	0	0	0	50	0	0	0	1	0	0	50	0	56.9
D_15_02349_DP_CS	1.024	52	1	0	0	67	1	1	0	0	0	1	70	1	1.1
D_15_02529_DP_CS	1.047	34	1	0	1	60	0	0	0	1	1	1	75	1	12.4
D_15_02522_DP_CS	1.047	62	0	0	1	67	0	0	0	1	1	1	75	1	6.6
D_15_02294_DP_CS	1.175	104	0	1	0	78	0	0	0	0	0	1	70	1	0.8
D_15_02420_DP_CS	1.308	41	0	0	0	31	0	0	1	1	1	0	50	1	14.9
D_15_02338_DP_CS	1.303	51	1	1	0	62	2	0	0	0	0	1	75	0	57.7
D_15_02539_DP_CS	1.406	63	0	0	1	57	0	0	0	1	1	1	75	0	30.9
D_15_02482_DP_CS	1.189	116	0	0	0	41	0	0	1	1	0	1	60	0	11.2
D_15_02515_DP_CS	0.874	33	1	1	0	74	0	1	1	0	0	1	70	0	33.5
D_15_02154_DP_CS	1.475	75	1	0	1	52	0	0	0	0	2	2	70	1	12.9
D_15_02352_DP_CS	1.408	64	1	0	0	45	1	0	1	1	1	1	70	1	6.7
D_15_02498_DP_CS	1.306	63	1	1	0	51	0	0	1	0	0	0	75	0	34.7
D_15_02430_DP_CS	1.190	39	0	0	0	39	0	0	0	0	1	0	70	0	31.0
D_15_02212_DP_CS	1.068	31	0	0	1	58	0	0	1	0	0	1	70	1	13.7
D_15_02278_DP_CS	1.394	65	0	1	1	77	0	1	1	1	0	1	80	1	6.3
D_15_02556_DP_CS	1.604	104	0	1	1	67	0	0	1	1	0	0	70	0	24.1
D_15_02476_DP_CS	1.245	36	0	0	1	69	0	0	1	0	0	1	30	1	12.0
D_15_02443_DP_CS	1.312	81	1	0	0	72	0	0	1	1	1	1	70	1	0.5
D_15_02526_DP_CS	1.308	70	0	0	0	61	0	1	0	0	0	1	80	0	33.0
D_15_02534_DP_CS	1.250	28	1	1	1	51	1	0	0	0	1	1	70	0	31.6
D_15_02478_DP_CS	1.439	102	0	0	0	45	0	1	1	0	1	0	80	0	5.0
D_15_02528_DP_CS	1.281	74	0	1	1	45	0	1	1	0	1	1	80	0	31.7
D_15_02471_DP_CS	1.364	22	0	1	1	55	0	0	1	0	0	1	40	0	35.5
D_15_02484_DP_CS	1.149	30	1	0	0	58	0	0	0	0	0	0	60	0	36.6
D_15_02492_DP_CS	1.557	130	1	1	0	56	1	0	1	1	1	1	70	1	0.4

D_15_02457_DP_CS	1.268	67	1	1	0	54	0	0	1	0	0	1	75	1	14.6
D_15_02155_DP_CS	0.767	44	1	0	1	69	0	1	0	2	0	2	80	1	26.3
D_15_02507_DP_CS	1.194	75	0	0	0	57	0	0	1	1	1	1	80	1	21.1
D_15_02271_DP_CS	1.423	64	1	0	0	73	1	0	0	1	1	1	70	1	0.8
D_15_02500_DP_CS	1.469	118	0	0	0	70	1	0	1	1	1	1	75	1	15.6
D_15_02316_DP_CS	0.930	21	1	1	1	67	0	0	0	0	0	1	50	0	53.2
D_15_02470_DP_CS	1.115	95	1	1	1	50	0	0	0	1	1	1	50	1	7.2
D_15_02232_DP_CS	1.128	83	0	0	1	49	1	0	0	1	0	1	70	1	0.7
D_15_02323_DP_CS	1.233	45	1	1	1	49	1	0	0	1	1	1	70	1	15.8
D_15_02233_DP_CS	1.090	47	0	0	1	37	1	0	1	1	0	2	60	1	0.7
D_15_02502_DP_CS	1.234	44	1	0	0	50	0	1	0	0	0	0	85	0	29.0
D_15_02474_DP_CS	1.069	31	1	1	1	67	0	1	1	0	0	0	60	0	36.0
D_15_02448_DP_CS	1.588	119	0	1	0	52	1	0	1	1	0	1	80	1	0.6
D_15_02139_DP_CS	1.251	28	1	0	0	48	0	0	2	2	2	2	70	1	38.8
D_15_02282_DP_CS	1.225	32	0	0	0	63	1	1	1	1	1	1	50	1	16.2
D_15_02211_DP_CS	0.807	42	1	1	1	37	0	0	1	0	0	1	70	1	1.8
D_15_02514_DP_CS	0.942	65	1	0	1	53	1	0	0	0	1	1	70	1	0.5
D_15_02505_DP_CS	1.173	28	0	0	1	21	0	1	1	1	0	1	80	1	18.6
D_15_02453_DP_CS	0.720	38	0	1	1	39	2	0	0	1	0	0	60	1	5.6
D_15_02487_DP_CS	1.286	27	1	0	0	41	0	0	1	0	1	0	50	1	12.9
D_15_02512_DP_CS	1.662	83	0	0	1	55	0	0	1	1	0	1	70	0	30.1
D_15_02140_DP_CS	1.010	13	0	0	1	44	0	0	2	2	2	2	70	1	5.3
D_15_02202_DP_CS	1.143	49	0	1	1	27	1	0	1	1	1	1	70	1	10.0
D_15_02230_DP_CS	1.134	105	0	0	1	23	0	1	1	1	0	0	70	1	24.9
D_15_02295_DP_CS	1.473	109	0	0	1	62	1	0	1	1	0	1	60	1	0.7
D_15_02530_DP_CS	1.227	79	1	1	1	53	2	0	0	1	1	1	70	1	1.2
D_15_02334_DP_CS	1.762	98	0	1	1	66	1	0	0	1	0	1	70	1	1.0
D_15_02475_DP_CS	0.791	869	0	1	1	58	0	1	1	0	0	0	70	0	37.6
D_15_02360_DP_CS	1.614	134	0	1	1	38	0	0	1	1	0	0	70	1	7.5
D_15_02555_DP_CS	1.536	83	0	0	1	41	0	0	0	1	1	1	65	0	28.3
D_15_02431_DP_CS	1.010	67	0	1	1	68	1	0	1	0	1	1	70	1	3.1
D_15_02318_DP_CS	1.427	87	1	0	0	43	1	0	0	0	0	1	70	1	22.4
D_15_02312_DP_CS	1.112	32	1	1	1	54	0	0	1	0	0	1	50	1	20.6
D_15_02461_DP_CS	1.254	70	1	0	1	76	0	0	1	0	0	1	75	1	1.1
D_15_02189_DP_CS	1.446	94	0	1	1	41	0	0	0	0	0	1	70	1	19.3
D_15_02551_DP_CS	1.539	85	1	1	1	58	0	0	1	1	1	1	70	1	0.5
D_15_02536_DP_CS	1.089	51	1	0	1	58	0	0	0	0	0	1	65	1	11.8
D_15_02454_DP_CS	1.140	102	0	0	1	62	0	0	1	1	1	0	60	0	42.6
D_15_02320_DP_CS	1.000	61	1	0	0	72	1	0	0	1	0	1	70	1	20.9
D_15_02543_DP_CS	1.065	89	0	1	1	51	0	0	1	0	1	1	70	0	25.6
D_15_02495_DP_CS	1.190	55	1	1	0	66	0	1	0	0	0	1	80	0	29.8
D_15_02209_DP_CS	1.300	84	0	1	1	52	1	1	1	1	0	1	80	1	3.9
D_15_02291_DP_CS	1.349	29	1	1	1	52	0	0	1	1	1	1	80	1	14.8
D_15_02215_DP_CS	1.247	55	0	0	1	53	0	1	1	0	0	1	70	1	13.4
D_15_02287_DP_CS	1.592	82	1	0	1	62	1	1	1	1	0	1	80	1	0.6
D_15_02439_DP_CS	1.250	61	1	0	1	71	1	0	1	0	1	1	60	1	13.2
D_15_02183_DP_CS	0.982	69	0	1	1	48	0	0	1	0	1	1	70	1	46.9
D_15_02540_DP_CS	1.269	115	0	0	0	60	1	0	1	0	1	1	70	0	29.6
D_15_02299_DP_CS	1.535	121	1	1	0	60	1	0	1	1	1	1	75	1	0.5
D_15_02227_DP_CS	1.328	73	0	0	1	36	0	0	1	0	0	2	80	1	6.1
D_15_02219_DP_CS	1.028	91	0	0	1	48	0	0	1	1	0	1	60	1	13.9
D_15_02217_DP_CS	0.675	32	1	1	1	50	1	0	1	1	1	1	60	1	19.2
D_15_02297_DP_CS	1.156	59	0	0	1	55	1	0	1	1	1	1	70	1	0.8
D_15_02343_DP_CS	1.271	78	1	1	1	49	0	0	1	1	1	1	70	1	23.4
D_15_02452_DP_CS	1.180	91	0	1	1	67	1	0	1	0	1	1	75	1	0.9
D_15_02160_DP_CS	1.112	79	0	1	0	47	1	1	1	0	0	2	70	1	9.9
D_15_02326_DP_CS	1.302	118	0	0	1	49	0	0	0	1	0	1	70	1	29.5
D_15_02315_DP_CS	1.225	128	0	0	0	46	0	1	2	2	2	2	70	0	52.0
D_15_02451_DP_CS	1.104	29	1	0	0	63	0	1	1	1	1	1	60	0	42.2
D_15_02506_DP_CS	1.720	134	0	0	1	49	0	0	1	1	1	1	80	1	15.2
D_15_02272_DP_CS	1.151	93	0	0	1	34	1	0	0	1	1	1	70	1	54.9
D_15_02508_DP_CS	1.481	73	1	1	1	53	1	0	1	1	0	1	70	1	11.5
D_15_02494_DP_CS	1.153	107	0	1	1	61	1	0	1	0	0	1	70	0	34.7
D_15_02339_DP_CS	1.029	50	1	1	0	49	0	0	1	0	0	1	60	0	44.2

D_15_02341_DP_CS	1.274	89	1	0	1	57	1	0	1	1	1	1	70	1	1.4
D_15_02290_DP_CS	1.182	109	1	0	1	63	1	0	1	1	0	1	80	1	0.8
D_15_02344_DP_CS	1.000	42	1	0	1	53	1	0	0	0	0	1	70	1	8.4
D_15_02504_DP_CS	1.325	93	0	1	1	43	1	0	1	1	1	1	75	0	32.5
D_15_02345_DP_CS	1.522	100	1	0	0	51	1	0	0	1	0	1	70	1	17.9
D_15_02511_DP_CS	1.721	174	0	0	1	74	0	0	1	0	0	0	80	1	12.3
D_15_02547_DP_CS	1.495	155	1	0	1	46	0	1	0	1	0	1	70	0	20.8
D_15_02201_DP_CS	1.331	116	0	0	1	65	1	0	1	1	0	0	80	1	4.4
D_15_02298_DP_CS	1.519	69	1	1	0	58	1	0	0	1	0	1	75	1	16.3
D_15_02501_DP_CS	1.073	43	0	0	0	66	0	0	1	1	1	0	80	1	16.5
D_15_02355_DP_CS	1.092	81	1	1	1	61	1	0	1	1	1	1	65	1	5.7
D_15_02216_DP_CS	1.201	58	0	1	1	50	0	0	0	1	1	1	60	1	9.8
D_15_02532_DP_CS	1.478	73	0	1	1	64	0	1	0	0	0	0	80	0	32.2
D_15_02235_DP_CS	1.485	80	0	1	1	65	0	1	1	1	1	1	60	1	19.2
D_15_02273_DP_CS	1.139	73	0	0	1	55	0	0	1	1	0	0	80	1	0.7
D_15_02423_DP_CS	1.530	101	1	0	1	72	1	0	1	0	0	1	70	0	50.6
D_15_02340_DP_CS	1.525	86	0	0	1	60	1	0	0	0	0	1	70	1	0.9
D_15_02283_DP_CS	1.207	53	0	1	0	52	0	0	1	1	1	1	40	1	6.6
D_15_02477_DP_CS	1.166	61	1	1	1	37	1	0	0	0	0	0	70	1	12.2
D_15_02521_DP_CS	1.409	30	1	1	0	67	0	1	0	0	0	1	75	0	27.8
D_15_02447_DP_CS	1.435	98	0	1	0	52	1	0	1	1	1	1	75	1	17.6
D_15_02221_DP_CS	1.417	32	0	1	1	56	0	0	0	0	0	2	50	1	32.5
D_15_02274_DP_CS	1.381	79	0	0	1	59	1	0	1	0	1	1	70	1	11.7
D_15_02426_DP_CS	1.186	55	0	1	1	39	1	0	1	1	0	0	70	1	0.5
D_15_02416_DP_CS	1.691	63	0	0	1	57	0	0	1	0	1	1	70	1	37.6
D_15_02473_DP_CS	1.350	93	0	0	0	48	1	0	1	0	1	1	80	1	6.4
D_15_02222_DP_CS	1.320	122	0	0	1	60	0	0	1	0	0	1	70	0	60.0
D_15_02455_DP_CS	1.345	42	0	0	0	45	1	1	1	1	1	1	70	1	0.7
D_15_02436_DP_CS	1.180	58	1	0	1	46	0	0	1	0	0	0	70	1	5.1
D_15_02279_DP_CS	1.344	72	1	0	0	59	1	0	1	1	0	1	70	0	28.4
D_15_02358_DP_CS	1.041	28	0	0	0	56	1	0	1	0	1	1	60	1	0.7
D_15_02545_DP_CS	1.275	128	0	0	0	44	1	1	1	1	0	1	70	1	20.9
D_15_02146_DP_CS	1.825	116	0	1	0	54	2	1	1	0	0	2	80	1	24.9
D_15_02527_DP_CS	1.256	109	0	0	0	40	1	1	1	1	1	1	70	0	28.5
D_15_02480_DP_CS	1.616	116	0	1	0	48	1	0	0	0	0	0	80	0	36.2
D_15_02276_DP_CS	1.568	127	0	1	1	54	0	0	0	1	0	1	80	1	11.1
D_15_02445_DP_CS	1.461	96	0	0	1	37	1	0	1	1	1	0	70	1	7.2
D_15_02524_DP_CS	1.057	103	0	1	1	38	0	0	0	0	0	0	70	1	6.2
D_15_02537_DP_CS	1.441	52	1	1	1	52	0	1	0	0	0	0	80	0	30.8
D_15_02542_DP_CS	1.464	47	1	0	0	52	1	0	1	1	1	1	85	1	6.9
D_15_02281_DP_CS	1.620	94	1	1	1	57	1	0	1	1	0	1	70	1	0.7
D_15_02415_DP_CS	1.194	102	0	1	1	52	0	1	1	0	0	1	90	1	1.1
D_15_02468_DP_CS	1.057	78	0	1	1	70	1	1	1	0	0	1	70	1	6.3
D_15_02489_DP_CS	1.531	86	0	1	1	74	1	1	0	0	0	1	75	1	3.7
D_15_02331_DP_CS	1.097	248	1	1	1	38	0	0	0	0	1	1	70	1	0.5
D_15_02220_DP_CS	1.292	83	0	1	1	48	0	0	1	0	0	2	70	1	50.4
D_15_02304_DP_CS	1.745	109	1	0	1	44	1	0	0	1	1	0	90	1	17.2
D_15_02464_DP_CS	1.146	101	0	0	1	49	0	0	1	1	1	1	70	1	0.1
D_15_02205_DP_CS	1.359	44	1	1	0	37	0	0	0	0	0	1	70	1	19.6
D_15_02333_DP_CS	1.480	38	1	1	1	48	1	0	1	1	0	1	50	1	10.1
D_15_02157_DP_CS	1.421	74	0	1	1	52	0	0	1	1	1	2	75	1	0.9
D_15_02552_DP_CS	1.204	78	1	1	0	64	0	0	1	0	0	1	70	0	24.8
D_15_02361_DP_CS	1.453	119	1	0	1	66	1	0	1	1	1	1	70	0	6.0
D_15_02325_DP_CS	1.194	45	1	0	1	61	0	0	0	1	1	1	50	1	22.6
D_15_02185_DP_CS	1.404	97	0	1	1	74	0	0	0	0	0	0	75	1	25.9
D_15_02460_DP_CS	1.353	93	1	0	1	46	0	0	1	0	1	1	80	1	15.0
D_15_02362_DP_CS	1.316	164	0	0	1	63	1	0	1	1	1	1	70	1	3.3
D_15_02427_DP_CS	1.276	36	1	1	0	35	0	0	1	0	1	0	70	1	2.5
D_15_02293_DP_CS	1.057	48	0	0	0	68	1	1	1	0	0	0	70	1	25.8
D_15_02429_DP_CS	0.943	259	1	0	0	35	1	0	1	0	0	1	70	1	4.7
D_15_02153_DP_CS	1.128	58	0	1	1	55	0	0	1	1	1	2	80	1	20.8
D_15_02190_DP_CS	1.238	82	0	1	1	46	0	0	0	0	0	0	70	1	17.9
D_15_02538_DP_CS	1.378	91	0	0	1	61	1	0	1	0	1	0	85	0	31.0
D_15_02265_DP_CS	1.420	90	1	0	0	63	0	1	0	1	0	1	70	1	9.8

D_15_02292_DP_CS	1.349	47	1	1	0	64	1	1	1	0	0	1	65	1	0.5
D_15_02186_DP_CS	1.644	137	1	0	1	73	0	0	1	0	1	1	80	1	18.4
D_15_02170_DP_CS	1.107	103	0	0	1	54	0	0	0	0	0	2	70	0	60.0
D_15_02286_DP_CS	1.250	84	0	0	1	43	0	1	1	1	0	1	80	1	10.4
D_15_02321_DP_CS	1.045	32	1	1	1	34	0	0	1	1	1	1	70	1	12.2
D_15_02441_DP_CS	1.377	143	0	1	0	50	0	1	0	0	0	1	80	1	19.5
D_15_02433_DP_CS	1.681	62	0	0	1	49	0	0	0	1	0	0	60	0	48.6
D_15_02142_DP_CS	1.291	122	0	0	1	63	1	0	2	2	2	2	70	0	60.0
D_15_02488_DP_CS	1.249	104	0	1	1	57	0	1	1	1	0	0	75	1	16.0
D_15_02175_DP_CS	1.569	101	0	0	1	38	1	0	0	0	0	2	80	1	4.0
D_15_02459_DP_CS	1.590	155	0	0	1	64	1	0	0	1	0	0	75	1	11.9
D_15_02467_DP_CS	1.350	62	1	1	1	50	0	0	0	0	0	0	60	0	36.1
D_15_02533_DP_CS	1.526	129	1	1	0	72	1	0	1	1	1	1	75	1	0.7
D_15_02270_DP_CS	1.574	134	0	0	0	60	1	0	1	1	0	1	70	1	14.4
D_15_02277_DP_CS	1.180	72	1	1	0	49	1	0	1	1	1	0	70	1	7.8
D_15_02187_DP_CS	1.271	56	0	1	1	46	0	0	1	1	0	1	80	0	29.7
D_15_02268_DP_CS	1.431	45	0	0	1	60	0	0	1	0	1	1	80	1	24.4
D_15_02531_DP_CS	1.438	108	1	0	1	67	1	0	1	1	1	1	70	1	0.5
D_15_02523_DP_CS	1.358	70	1	1	0	57	1	0	0	1	0	1	70	1	0.7
D_15_02444_DP_CS	1.590	98	0	1	0	52	0	1	1	1	1	1	60	0	43.5
D_15_02481_DP_CS	1.684	147	0	0	0	43	0	1	1	1	1	0	60	1	3.1
D_15_02314_DP_CS	1.240	87	0	0	1	66	1	0	0	0	1	1	70	1	11.1
D_15_02472_DP_CS	1.233	62	1	0	1	62	0	0	0	0	1	1	70	1	3.7
D_15_02438_DP_CS	1.316	98	1	0	1	50	0	0	1	0	0	1	70	1	13.8
D_15_02363_DP_CS	1.502	172	0	0	1	30	1	0	1	1	1	1	70	1	0.6
D_15_02490_DP_CS	1.286	32	1	1	0	60	0	0	1	0	1	1	80	0	35.6
D_15_02432_DP_CS	1.305	165	0	0	1	69	1	1	1	1	0	1	70	1	4.2
D_15_02350_DP_CS	1.176	41	1	1	1	43	1	1	0	0	1	0	50	1	15.9
D_15_02164_DP_CS	1.271	63	1	1	0	53	0	1	0	0	0	2	80	1	18.2
D_15_02203_DP_CS	1.494	84	0	0	1	46	0	0	1	0	1	0	90	1	14.3
D_15_02181_DP_CS	1.452	87	0	0	1	61	1	1	1	1	1	2	70	1	14.3
D_15_02317_DP_CS	1.522	113	0	0	1	35	1	0	1	1	0	1	70	1	12.2
D_15_02218_DP_CS	1.640	105	0	0	1	65	1	1	2	2	2	2	70	1	12.0
D_15_02446_DP_CS	1.344	67	1	0	0	39	1	1	1	1	0	1	70	1	6.6
D_15_02168_DP_CS	1.538	109	1	0	1	68	1	1	0	0	0	2	75	1	45.2
D_15_02195_DP_CS	1.700	92	0	1	1	39	1	0	0	0	1	1	80	1	14.1
D_15_02513_DP_CS	1.576	145	1	1	1	61	0	0	1	1	1	1	75	1	23.3
D_15_02224_DP_CS	1.432	70	1	1	1	54	1	0	1	0	0	2	80	1	0.6
D_15_02496_DP_CS	1.756	153	0	0	1	47	0	0	1	0	0	1	80	0	31.7
D_15_02302_DP_CS	1.029	76	1	1	1	58	1	1	0	0	0	1	70	1	1.2
D_15_02356_DP_CS	1.333	62	0	0	0	69	0	1	1	1	0	1	70	1	18.7
D_15_02469_DP_CS	1.328	121	1	1	1	55	0	0	1	0	1	1	70	1	13.4
D_15_02306_DP_CS	1.390	115	1	1	1	60	1	0	0	1	0	1	70	1	2.0
D_15_02328_DP_CS	0.929	52	1	0	1	36	0	0	0	0	0	0	75	0	56.7
D_15_02463_DP_CS	1.658	92	0	0	0	54	0	0	0	0	1	1	70	1	3.7
D_15_02442_DP_CS	1.432	101	1	1	1	52	1	0	1	1	1	1	60	1	1.3
D_15_02336_DP_CS	1.342	29	1	0	1	67	1	0	1	1	0	1	50	1	4.7
D_15_02143_DP_CS	1.285	62	1	1	1	65	2	0	2	2	2	2	80	1	26.9
D_15_02422_DP_CS	1.407	62	1	0	1	57	1	0	0	0	0	1	70	1	6.4
D_15_02280_DP_CS	1.294	79	1	0	1	49	1	0	1	1	1	1	65	1	40.7
D_15_02329_DP_CS	1.329	65	1	1	0	62	0	0	1	1	1	1	75	1	16.0
D_15_02322_DP_CS	1.061	64	0	0	1	35	1	1	1	1	1	1	70	1	2.6
D_15_02354_DP_CS	1.309	94	0	0	1	51	0	1	0	1	0	1	75	1	25.3
D_15_02177_DP_CS	1.394	69	1	0	0	53	0	0	0	0	0	2	70	1	12.8
D_15_02301_DP_CS	1.389	52	1	1	1	75	0	0	1	1	1	1	70	1	3.6
D_15_02148_DP_CS	1.275	77	0	1	0	41	1	0	0	0	2	2	80	1	10.8
D_15_02359_DP_CS	1.541	131	0	1	1	58	0	1	0	0	0	0	70	1	6.6
D_15_02231_DP_CS	0.867	40	0	0	1	54	2	1	1	1	0	2	70	1	6.9
D_15_02191_DP_CS	1.680	134	0	0	1	50	1	0	1	1	0	0	70	1	7.1
D_15_02145_DP_CS	1.363	103	0	1	1	46	1	0	0	0	2	2	70	1	38.8
D_15_02554_DP_CS	1.522	49	1	0	0	65	1	0	1	1	1	1	60	1	2.5
D_15_02499_DP_CS	1.200	143	0	1	1	52	0	1	0	0	0	1	75	0	16.3
D_15_02497_DP_CS	1.220	29	1	0	0	56	0	0	0	0	1	1	60	0	33.2
D_15_02435_DP_CS	1.763	142	1	1	0	63	0	0	1	1	1	1	85	1	1.0

D_15_02174_DP_CS	1.231	54	0	0	0	63	1	0	0	1	0	2	70	1	29.3
D_15_02179_DP_CS	1.469	114	1	1	1	59	1	0	1	0	1	2	70	1	32.3
D_15_02194_DP_CS	0.815	42	0	0	1	33	0	1	1	0	0	0	60	1	29.2
D_15_02357_DP_CS	1.462	36	1	1	0	59	0	0	1	0	0	1	70	1	8.2
D_15_02225_DP_CS	1.444	91	0	1	0	53	0	0	0	0	0	2	70	1	7.7
D_15_02289_DP_CS	1.380	58	1	1	0	49	0	1	0	0	0	0	75	1	12.5
D_15_02200_DP_CS	1.447	142	1	1	0	57	0	0	1	0	1	1	80	1	38.2
D_15_02465_DP_CS	1.579	55	1	0	1	49	1	0	0	0	1	0	70	1	0.5
D_15_02208_DP_CS	1.036	75	0	1	1	65	0	0	1	0	1	1	70	1	6.6
D_15_02353_DP_CS	0.716	54	0	0	1	38	1	1	0	1	1	1	70	1	7.0
D_15_02149_DP_CS	1.593	94	1	1	0	45	1	0	0	0	2	2	70	1	40.6
D_15_02197_DP_CS	1.251	99	0	0	1	69	0	0	0	0	0	1	80	0	47.4
D_15_02266_DP_CS	1.450	94	1	0	1	48	1	0	1	0	0	1	80	1	24.9
D_15_02434_DP_CS	1.736	340	0	1	1	55	0	0	0	1	0	1	70	1	0.9
D_15_02184_DP_CS	1.433	89	0	0	1	31	2	1	1	0	0	1	65	1	33.0
D_15_02150_DP_CS	1.608	98	0	0	0	45	0	1	0	0	2	2	90	1	55.3
D_15_02437_DP_CS	1.544	82	1	1	1	47	1	0	1	1	1	1	80	1	0.7
D_15_02458_DP_CS	1.502	120	0	1	0	35	0	0	1	1	1	1	75	1	9.1
D_15_02544_DP_CS	1.339	291	1	1	1	44	0	0	0	0	0	0	70	0	25.3
D_15_02188_DP_CS	1.095	91	0	1	1	64	0	0	0	0	1	1	80	1	19.9
D_15_02269_DP_CS	1.859	160	0	1	1	59	1	0	1	0	1	1	80	1	0.6
D_15_02351_DP_CS	1.245	30	1	1	1	54	0	0	1	1	0	1	50	0	56.3
D_15_02199_DP_CS	1.065	57	1	1	1	41	1	0	1	1	0	1	70	1	0.5
D_15_02144_DP_CS	1.255	93	0	1	0	57	0	0	0	0	0	2	80	1	21.2
D_15_02450_DP_CS	1.312	98	1	0	1	57	1	0	0	0	1	1	70	1	3.0
D_15_02213_DP_CS	1.069	54	0	1	1	48	0	0	1	1	0	1	80	1	18.2
D_15_02234_DP_CS	1.404	88	0	1	1	70	1	0	1	0	0	2	70	1	24.3
D_15_02347_DP_CS	1.408	47	0	1	0	53	1	0	1	0	0	1	75	0	55.6
D_15_02311_DP_CS	1.542	77	1	0	1	48	1	0	1	0	0	1	70	1	0.7
D_15_02284_DP_CS	1.763	150	0	0	0	77	0	0	0	0	0	1	75	1	15.4
D_15_02141_DP_CS	1.204	66	0	0	1	54	0	1	1	1	2	2	80	1	48.2
D_15_02417_DP_CS	1.562	29	1	1	0	74	0	1	1	0	0	0	80	1	7.1
D_15_02313_DP_CS	0.888	24	1	1	1	63	1	1	2	2	2	2	70	1	12.0
D_15_02414_DP_CS	0.990	76	1	1	0	35	2	0	1	1	1	0	65	1	1.0
D_15_02223_DP_CS	1.201	45	0	1	1	70	0	0	0	0	1	2	70	0	22.4
D_15_02319_DP_CS	0.927	47	1	1	1	69	1	1	1	1	1	1	50	0	5.5
D_15_02206_DP_CS	1.254	103	1	1	1	60	0	0	0	0	0	2	60	1	17.8
D_15_02285_DP_CS	1.420	70	0	1	1	59	1	0	0	0	0	1	80	1	1.5
D_15_02424_DP_CS	1.196	184	1	0	1	40	1	0	2	2	2	2	70	0	7.0
D_15_02332_DP_CS	1.200	24	0	0	1	46	0	0	0	0	0	0	50	0	60.0
D_15_02428_DP_CS	1.548	105	0	0	1	73	0	0	1	0	1	0	75	1	4.2
D_15_02163_DP_CS	1.409	138	0	0	1	61	0	0	1	1	0	2	80	1	48.0
D_15_02412_DP_CS	1.052	64	0	0	1	58	1	0	1	1	0	0	70	1	11.6
D_15_02309_DP_CS	1.458	73	0	1	1	38	1	0	0	0	1	0	70	1	3.1
D_15_02413_DP_CS	1.821	130	0	0	1	48	0	0	1	0	0	0	70	1	36.5
D_15_02303_DP_CS	1.245	50	1	1	1	58	1	0	0	1	1	1	70	1	20.4
D_15_02267_DP_CS	1.417	76	0	1	0	38	0	0	1	1	1	1	60	1	5.9
D_15_02310_DP_CS	0.831	114	0	1	0	39	2	0	0	0	0	0	50	1	0.5
D_15_02307_DP_CS	1.178	91	1	0	1	55	1	0	0	0	0	1	70	1	2.7
D_15_02520_DP_CS	1.224	26	1	1	1	52	0	0	0	0	0	0	70	0	30.7
D_15_02305_DP_CS	1.435	104	0	1	1	73	0	0	0	1	0	0	80	1	21.2
D_15_02180_DP_CS	1.027	43	1	0	1	72	0	1	2	2	2	2	70	1	26.3
D_15_02193_DP_CS	1.301	137	0	1	1	40	0	0	0	1	0	1	70	1	6.9
